# A Kinetic Map of the Homomeric Voltage-Gated Potassium Channel (Kv) Family

**DOI:** 10.3389/fncel.2019.00358

**Published:** 2019-08-20

**Authors:** Rajnish Ranjan, Emmanuelle Logette, Michela Marani, Mirjia Herzog, Valérie Tâche, Enrico Scantamburlo, Valérie Buchillier, Henry Markram

**Affiliations:** ^1^Blue Brain Project, Ecole Polytechnique Fédérale de Lausanne, Geneva, Switzerland; ^2^Laboratory of Neural Microcircuitry, Brain Mind Institute, Ecole Polytechnique Fédérale de Lausanne, Lausanne, Switzerland

**Keywords:** Kv channel, electrophysiology, automated patch clamp, kinetics, temperature, Q_10_, modeling, database

## Abstract

The voltage-gated potassium (Kv) channels, encoded by 40 genes, repolarize all electrically excitable cells, including plant, cardiac, and neuronal cells. Although these genes were fully sequenced decades ago, a comprehensive kinetic characterization of all Kv channels is still missing, especially near physiological temperature. Here, we present a standardized kinetic map of the 40 homomeric Kv channels systematically characterized at 15, 25, and 35°C. Importantly, the Kv kinetics at 35°C differ significantly from commonly reported kinetics, usually performed at room temperature. We observed voltage-dependent Q_10_ for all active Kv channels and inherent heterogeneity in kinetics for some of them. Kinetic properties are consistent across different host cell lines and conserved across mouse, rat, and human. All electrophysiology data from all Kv channels are made available through a public website (Channelpedia). This dataset provides a solid foundation for exploring kinetics of heteromeric channels, roles of auxiliary subunits, kinetic modulation, and for building accurate Kv models.

## Highlights

- A reference map of kinetics of all 40 homomeric Kv channels at 15, 25, and 35°C.
- Kv kinetics are consistent across cell lines and conserved across species.
- Some Kv channels exhibit inherent heterogeneity in kinetics.
- A public resource of over a million current traces from Kv channel kinetics.

## Introduction

Ion channels (ICs) are proteins that selectively allow ions to diffuse through the cell membrane, creating an electrical potential across the membrane. They are classified in terms of which ion passes through the channel (sodium, potassium, chloride, calcium etc.) and by gating activity (voltage gating, ligand gating, other forms of gating; Hille, 2001; Alberts et al., 2008).

**Figure 1:**
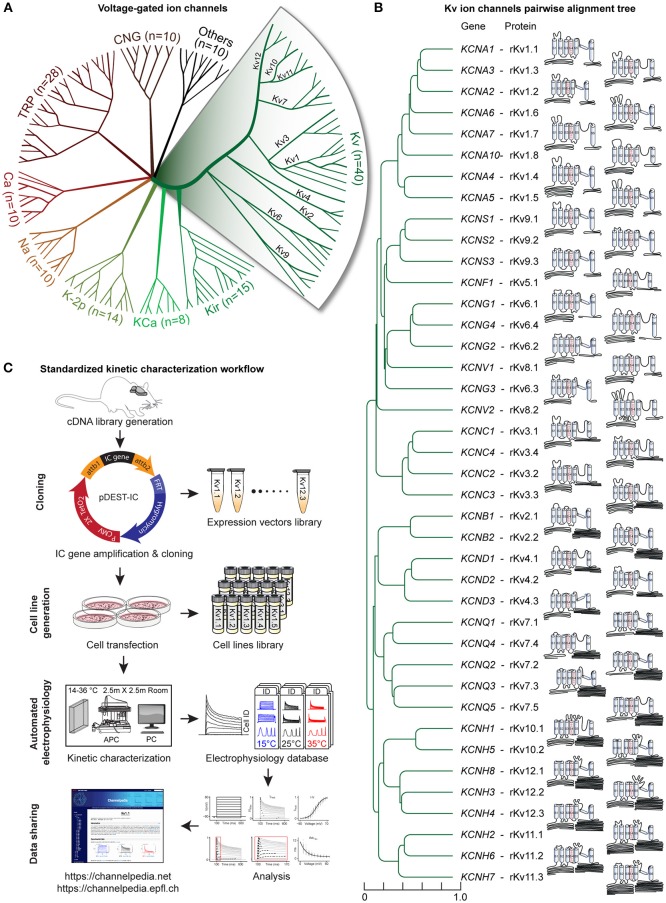
Voltage-gated potassium channels, kinetic characterization workflow. **(A)** Radial phylogenetic tree of the 145 known voltage-gated ion channel genes, where half are potassium channels (in green). Our study focuses on the 40 voltage-gated potassium (Kv) channels, divided into 12 subfamilies (expanded area). **(B)** Phylogenetic tree obtained from pairwise alignment of the coding sequences of isoform1 of all rat Kv channel genes; scale represents normalized phylogenetic distance; the corresponding Kv protein names are indicated (prefix r stands for rat). The schematic and scaled depiction of the protein structure of each Kv channel is based on the amino acid length of each domain. **(C)** Standardized workflow for Kv channels kinetic characterization. Each Kv gene is amplified from a rat brain cDNA library and cloned in a mammalian-expression vector (Table S6), building a library of expression vectors. A library of stable cell lines is generated after transfection of each expression vector in host cell lines (CHO, HEK, or CV1; See also Figures S1, S2 and Table S7). The kinetics of each cell line is characterized using an automated patch clamp setup (APC) in a dedicated temperature-controlled room (14–36°C; Table S8). Each recorded cell is assigned a unique ID (Cell ID). Electrophysiology data are analyzed, stored and shared through the “Channelpedia” website.

The **~350** IC types expressed in the mammalian brain include **145** voltage-gated channels, of which **40** are voltage-gated potassium (Kv) channels, divided into **12** sub-families, Kv1-Kv12 (Figure 1A). The Kv channels are expressed in many different tissues/organs, including muscle, heart, and brain (Post et al., 1992; Lai and Jan, 2006; Li and Dong, 2010; Southan et al., 2016), and also exhibit specific expression patterns at the subcellular level (Rasband, 2010; Jensen et al., 2011; Trimmer, 2015). In all these tissues, Kv channels are activated by variations in the voltage across the cell membrane. These changes regulate the return of the membrane to a resting state (hyperpolarization) after depolarization, thus controlling the excitability of different cell types (neurons, cardiomyocytes, skeletal muscle cells, etc.).

Functionally, Kv channels are known to regulate the threshold potential for firing, the duration of action potentials and, the firing rates (Storm, 1988; Gabel and Nisenbaum, 1998; Glazebrook et al., 2002; Begum et al., 2016). They are also involved in cell proliferation, and play a significant role in neurotoxicity, neuroprotection, and neuroregulation (Kaczmarek, 2006; Shah and Aizenman, 2014). Hence, malfunction in Kv channels are implicated in a range of neurological diseases known as channelopathies (Graves and Hanna, 2005; Kullmann and Waxman, 2010).

Structurally, mammalian Kv channels as homotetramers, consist of four identical alpha (α)-subunits arranged around a central axis that forms a pore (Coetzee et al., 1999). Each α-subunit is made up of six α-helical transmembrane spanning segments (S1-S6), five loops connecting successive segments, and cytoplasmic regions at the N and C terminal ends. The first four segments (S1–S4) form the voltage sensor domain (VSD). Within VSD, the fourth segment, S4, which contains a positively charged amino acid (arginine or lysine) at every third position (Bezanilla, 2000), is considered the main voltage sensor. The pore is formed by the S5–S6 segments and the corresponding connecting loop, which contains a conserved sequence (glycine-tyrosine-glycine or glycine-phenylalanine-glycine) that acts as a K^+^ ion selectivity filter (Heginbotham et al., 1994).

In mammalian genomes, the Kv channels are encoded by 40 genes. Each Kv gene encodes a corresponding protein (α-subunit) with a unique structure (Figure 1B). While all Kv channels share a similar core structure, differences in their connecting loops, and in the length and sequence of their N and C terminals, make each one of them unique. The genes coding for the Kv channels have been cloned and studied in cell lines for many decades. However, a comprehensive and standardized kinetic characterization of all homomeric Kv channels is still missing. The literature focuses on just a few Kv channels (e.g., Kv7.1, Kv11.1, Kv1.5, Kv1.1, Kv1.3, Kv1.2, Kv2.1, etc.), and neglects many others (e.g., Kv1.7, Kv1.8, Kv6.x, Kv9.x, Kv12.x; Table 1). Moreover, different studies have used different species, cloning procedures, host cell lines, cell culture conditions, intracellular and extracellular solutions, and stimulation protocols, making it difficult to compare and integrate their data and sometimes producing discrepant results. In addition, the raw electrophysiology data needed to build realistic IC models are not publicly available; available data are only in the form of figures or extracted features. Eventually, the majority of previous studies on Kv channels has been conducted at room temperature (RT), which can vary from 18°C (Heinemann et al., 1996) to 28°C (Hatton et al., 2001) that could produce inconsistencies in results.

**Table 1:** Number of publications on Kv channels in PubMed as of January 2019 (after search by gene names and/or protein names).

**S.No**	**Search on gene name**		**Search on protein name**		**Search on gene or protein name**
	***Gene***	**Count**	**Protein**	**Count**	**Total Count**
1	*KCNQ1*	1,978	Kv7.1	168	2,026
2	*KCNH2*	1,662	Kv11.1	169	1,745
3	*KCNA5*	322	Kv1.5	925	963
4	*KCNA1*	344	Kv1.1	899	967
5	*KCNA3*	201	Kv1.3	1,023	1,037
6	*KCNA2*	174	Kv1.2	855	878
7	*KCNB1*	265	Kv2.1	672	739
8	*KCNQ2*	692	Kv7.2	134	740
9	*KCND2*	166	Kv4.2	609	651
10	*KCND3*	166	Kv4.3	555	618
11	*KCNA4*	142	Kv1.4	561	577
12	*KCNH6*	546	Kv11.2	3	549
13	*KCNQ3*	407	Kv7.3	87	446
14	*KCNH1*	285	Kv10.1	52	309
15	*KCNC1*	79	Kv3.1	239	281
16	*KCNQ4*	247	Kv7.4	74	284
17	*KCNQ5*	141	Kv7.5	63	172
18	*KCNC4*	38	Kv3.4	138	150
19	*KCNA6*	30	Kv1.6	145	161
20	*KCNC3*	76	Kv3.3	89	118
21	*KCNB2*	28	Kv2.2	75	91
22	*KCNC2*	22	Kv3.2	74	85
23	*KCND1*	22	Kv4.1	67	72
24	*KCNV2*	56	Kv8.2	8	56
25	*KCNS3*	14	Kv9.3	38	49
26	*KCNH7*	40	Kv11.3	9	46
27	*KCNH5*	34	Kv10.2	5	37
28	*KCNA7*	12	Kv1.7	24	31
29	*KCNS1*	26	Kv9.1	7	28
30	*KCNF1*	13	Kv5.1	13	25
31	*KCNG3*	15	Kv6.3	9	19
32	*KCNH3*	17	Kv12.2	5	19
33	*KCNV1*	13	Kv8.1	8	16
34	*KCNH4*	16	Kv12.3	2	18
35	*KCNG1*	7	Kv6.1	10	17
36	*KCNG4*	6	Kv6.4	12	17
37	*KCNA10*	12	Kv1.8	3	15
38	*KCNH8*	10	Kv12.1	5	15
39	*KCNG2*	8	Kv6.2	4	12
40	*KCNS2*	3	Kv9.2	7	7

The thermal sensitivity of any biological process can be described by its temperature coefficient (Q_10_). Classically, it is defined as the ratio of a reaction rate (α) measured at two temperatures 10 degrees apart (Bělehrádek, 1935). In ion channel research, instead of reaction rates, current amplitudes or time constants are often used to calculate Q_10_ value. A single value of Q_10_ is typically reported to indicate the temperature dependence of a channel. However, it is important to note that the Q_10_ for an IC can be different for different temperature ranges (Beam and Donaldson, 1983). Moreover, the kinetic properties (activation, inactivation, deactivation, recovery from inactivation) of an IC can have different temperature dependence (Q_10_ value; Lee and Deutsch, 1990). These additional complexities have often been overlooked especially in IC modeling, where a single approximate Q_10_ value between 1 and 5 is used to account for temperature dependence. This approximation is used mainly because of the limited number of studies conducted near physiological temperature (last column of Table 2).

**Table 2:** Summary of reported inactivation patterns at room temperature or at higher temperature for each Kv channel, with references (detailed references are listed in supplementary document).

**IC**	**RT (18–28**^****°****^**C)**		**High T^**°**^ (30–39^**°**^C)**
Kv1.1	Non-inactivating (18–22°C, 24–28°C)	Mourre et al., 1999; Hatton et al., 2001; Sankaranarayanan et al., 2005; Al-Sabi et al., 2013; D'Adamo et al., 2014; García-Fernández et al., 2016; Mestre et al., 2016	**No data**
	Slow inactivating/inactivating (19–22°C)	Stühmer et al., 1989; Bertoli et al., 1994; Elinder et al., 1996; Gòmez-Hernandez et al., 1997	
	Fast inactivating when co-expressed with Kvβ1.1 or with Kvβ3 (RT, 17–18°C)	Heinemann et al., 1996; Bähring et al., 2004	
Kv1.2	Non-inactivating (RT, 20°C)	Heinemann et al., 1996; Sprunger et al., 1996; Mourre et al., 1999; Akhtar et al., 2002; Ferber et al., 2004	Activation and Inactivation time constants decrease with increase in temperature **(23 vs. 32°C;** Russell et al., 1994
	Slow inactivating (RT)	Po et al., 1993; Xie et al., 2010; Lee et al., 2011; Syrbe et al., 2015	
	Variable (22–23°C)	Rezazadeh et al., 2007	
	Fast inactivating when co-expressed with Kvβ1.1 or with Kvβ3 (RT, 17–18°C)	Heinemann et al., 1996; Bähring et al., 2004	
Kv1.3	Slow inactivating (RT)	Mourre et al., 1999; Rangaraju et al., 2010; Lee et al., 2011; Zhu et al., 2011; Kinoshita et al., 2012	**No data**
	Fast Inactivating (20–24°C)	Nicolaou et al., 2007; Hou et al., 2014; Yan et al., 2015	
	Become non-inactivating at higher expression (UKN)	Honoré et al., 1992	
Kv1.4	Fast inactivating (20–25°C)	Bertoli et al., 1994; Akhtar et al., 1999; Sankaranarayanan et al., 2005; Xie et al., 2014; Chen et al., 2016	**No data**
Kv1.5	Non-inactivating (20–24°C)	Po et al., 1993; Wible et al., 1998; Kuryshev et al., 2001; Remillard et al., 2007; Tipparaju et al., 2012; Wang et al., 2016	Inactivation time constant decreases with increase in temperature. Wettwer and Terlau, 2014 **(24 vs. 37°C)**, Rich and Snyders, 1998; **22 vs. 34°C)**.
	Slow inactivating (22–24°C)	Yang et al., 1997; Jeong et al., 2012; Lee et al., 2016; Wang et al., 2016	
	Fast inactivating when co-expressed with Kvβ1.1, Kvβ3 or Kvβ1.3 (RT, 17–18°C)	Heinemann et al., 1996; Bähring et al., 2004; Tipparaju et al., 2012	
Kv1.6	Non-inactivating (18–22°C)	Roeper et al., 1998; Ferber et al., 2004; Aguilar et al., 2010; Rangaraju et al., 2010; Zhu et al., 2011; García-Fernández et al., 2016	Channel inactivates near physiological temperature **(35°C;** Guihard et al., 2003
	Slow inactivating (UKN)	Bertoli et al., 1994; Guihard et al., 2003	
	Inactivating when co-expressed with Kvβ3 (RT)	Bähring et al., 2004	
Kv1.7	Slow or fast inactivating depending on the isoform or species (20–22°C)	Kalman et al., 1998; Bardien-Kruger et al., 2002; Finol-Urdaneta et al., 2006	**No data**
Kv1.8	Non-inactivating (UKN)	Lang et al., 2000; Tian et al., 2002	**No data**
Kv2.1	Non-inactivating (20–26°C)	Wible et al., 1997; Blaine and Ribera, 1998; Malin and Nerbonne, 2002; Kihira et al., 2010	Activation and Inactivation time constant decrease with increase in temperature **(32–35°C)** MacDonald et al., 2003; Zhong et al., 2010; Yang and Zheng, 2014
	Slow inactivating (20-22 °C)	Kerschensteiner et al., 2003, 2005; Aréchiga-Figueroa et al., 2015	
Kv2.2	Non-inactivating (22–26°C)	Blaine and Ribera, 1998; Malin and Nerbonne, 2002; Kihira et al., 2010; Dong et al., 2013	**No data**
Kv3.1	Slow inactivating (22–23°C)	Diochot et al., 1998; Sung et al., 2009	Inactivation time constant decreases with increase in temperature **(27, 36, 39°C)** Oliver et al., 2017
	Non-inactivating (RT)	Weiser et al., 1994; Moreno et al., 1995; Rudy et al., 1999	
	Non-inactivating with sharp inactivation peak at start (RT, 22–25°C)	Kanemasa et al., 1995; Macica et al., 2003; Lewis et al., 2004; Kanda et al., 2011; Brown et al., 2016	
Kv3.2	Non-inactivating (RT, 21–23°C)	Weiser et al., 1994; Moreno et al., 1995; Hernández-Pineda et al., 1999; Rudy et al., 1999	**No data**
	Non-inactivating with sharp inactivation peak at start (RT)	Lewis et al., 2004	
Kv3.3	Non-inactivating (UKN)	Rudy et al., 1999	**No data**
	Non-inactivating with sharp inactivation peak at start (RT)	Rashid et al., 2001	
	Slow inactivating (RT)	Kanda et al., 2011; Zhang et al., 2016	
	Fast inactivating (20–22°C)	Weiser et al., 1994; Diochot et al., 1998; Mock et al., 2010	
	Non-or slow inactivating depending on the cell line (UKN)	Fernandez et al., 2003	
Kv3.4	Fast inactivating (20–24°C)	Weiser et al., 1994; Elinder et al., 1996; Diochot et al., 1998; Rudy et al., 1999; Baranauskas et al., 2003; Yeung et al., 2005; Kanda et al., 2011	**No data**
Kv4.1	Fast inactivating outward A-type current (20–23°C)	Jerng et al., 1999; Beck et al., 2002; Yunoki et al., 2014	**No data**
Kv4.2	Fast inactivating outward A-type current (RT, 20–23°C)	Pérez-García et al., 1999; Jerng et al., 2004; Yunoki et al., 2014; Abbott, 2016	**No data**
	Low current (RT)	Zhou et al., 2004	
Kv4.3	Fast inactivating outward A-type current (20–24°C)	Beck et al., 2002; Jeong et al., 2011; Yunoki et al., 2014	Activation and Inactivation rates increase with increase in temperature **(23–39°C)** Yang and Zheng, 2014
Kv7.1	Non-inactivating (22–24°C)	Gamper et al., 2003; Zaika et al., 2008; Oliveras et al., 2014	Increasing the temperature decreases the half-activation time **(20 vs. 37°C)** Unsöld et al., 2000
	Non-inactivating with inactivation peak at start (RT)	Tinel et al., 2000; Yang et al., 2004	
Kv7.2	Non-inactivating (UKN)	Gamper et al., 2003; Li et al., 2004; Peretz et al., 2010; Orhan et al., 2014	**No data**
	Silent or low current becomes more active as heteromers with Kv7.3 (RT, 25°C)	Wang et al., 1998; Jentsch, 2000; Nakajo and Kubo, 2008; Zaika et al., 2008; Choveau et al., 2012a	
	Active or silent depending on the isoform (20–22°C)	Smith et al., 2001	
Kv7.3	Non-inactivating (RT)	Gamper et al., 2003; Zaika et al., 2008; Choveau et al., 2012a	**No data**
	Silent or low current; becomes more active as heteromers with Kv7.2 (RT)	Wang et al., 1998; Jentsch, 2000; Miceli et al., 2009; Choveau et al., 2012a	
Kv7.4	Non-inactivating	Schrøder et al., 2001; Li et al., 2004; Choveau et al., 2012b; Blom et al., 2014	**No data**
Kv7.5	Non-inactivating, low current (RT, 22–25°C)	Gamper et al., 2003; Li et al., 2004; Bentzen et al., 2006; Blom et al., 2009	**No data**
	More active as heteromers with Kv7.3 (RT)	Gilling et al., 2013; Kim et al., 2016	
Kv10.1	Non-inactivating (20–22°C)	Ju and Wray, 2002; Schönherr et al., 2002; Hsu et al., 2012; Chuang et al., 2014	Activation time constant decreases with increase in temperature **(25 vs. 30°C)** Mortensen et al., 2015
Kv10.2	Non-inactivating (20–22°C)	Ju and Wray, 2002; Schönherr et al., 2002; Hsu et al., 2012; Yang et al., 2013; Chuang et al., 2014	**No data**
Kv11	This family requires special 2 pulse protocol for kinetic characterization		
Kv12.1	Non-inactivating (22–24°C)	Zou et al., 2003; Zhang et al., 2009; Kazmierczak et al., 2013	**No data**
Kv12.2	Low-current, non-inactivating with inactivation peak at start (21–25°C)	Trudeau et al., 1999; Clancy et al., 2009; Noma et al., 2009	**No data**
Kv12.3	Non-inactivating (25°C)	Miyake et al., 1999	**No data**

*Temperature is indicated when known (RT, room temperature; UKN, unknown temperature)*.

Taken together, the non-standardized experimental conditions, the lack of studies near physiological temperature and the unavailability of the electrophysiological traces, prevent reaching a consensus on Kv channel's kinetic properties. We have therefore developed a standardized method to systematically characterize the electrophysiology of the homomeric Kv channels at different temperatures: 15 and 25°C to compare against the literature, and 35°C to provide data on the behavior of these channels near physiological temperature. Briefly, we cloned the Kv genes from the rat brain and generated a library of isogenic cell lines over-expressing single homomeric Kv channels, in an inducible manner. We then used automated patch clamping to characterize the biophysics of each channel. This allowed us to construct comprehensive maps of the kinetics of the homomeric Kv channels at 15, 25, and at 35°C—a temperature close to physiological conditions.

The large amount of data produced using automated patch clamping, especially near physiological temperature, allowed us to observe unexpected qualitative changes for specific Kv channels, beside quantitative changes in kinetics associated with changes in temperature. We found that the Q_10_ is non-linear not only with temperature but also with voltage. Furthermore, the systematic kinetic characterization revealed that some ICs exhibit inherent kinetic variability (heterogeneity), which might have been overlooked as artifacts. We illustrate how these data on temperature and voltage dependencies in IC kinetics can also be used to build more accurate temperature-dependent models of Kv channels, using the data for the Kv1.1 channel as an example. Contrarily to previous reports (Petersen and Nerbonne, 1999; Fernandez et al., 2003) that show that host cell lines affect Kv kinetic properties, we show that, under standardized conditions and with large sample sizes, the kinetic properties of the homomeric Kv channels are largely consistent across host cell lines (CHO, HEK, CV1). We also show that kinetics is well-conserved across mouse, rat, and human species. The raw and processed data from our study have been made publicly available through Channelpedia (https://channelpedia.net or https://channelpedia.epfl.ch)—a web-based wiki-like resource.

## Results

### Standardized Kinetic Characterization of Kv Channels

To establish a standardized kinetic map of the Kv family, we developed a screening workflow for the kinetic characterization of Kv channels. The workflow consists of four main steps: cloning, cell line generation, automated electrophysiology, and data sharing (Figure 1C).

Heterologous over-expression of ICs in host cell lines is commonly used to characterize IC kinetics. Previous studies have used many different host cell lines (e.g., CHO, COS7, CV1, HEK, LTK, ND7-23, NG108, NIH3T3; Lalik et al., 1993; Stephens et al., 1997; John et al., 2004). We chose CHO cells as they meet a broad range of criteria for successful IC experiments (Gamper et al., 2005), in particular: an efficient exogenous expression of recombinant proteins, very low endogenous ionic current and a good compatibility with planar automated patch clamp method. To ensure highly standardized conditions, we produced isogenic cell lines using the Flp-In^™^ system (O'Gorman et al., 1991), which provides reproducible conditions within and across different cell lines. We combined the Flp-In system with the tetracycline induction system T-Rex^™^, that enables expression of IC on demand, avoiding possible side effects of constitutive expression (Yao et al., 1998).

We generated a cDNA library from rat brain tissue, and attempted to amplify the 40 known Kv coding genes using the corresponding RefSeqs from the GenBank database (NCBI, Resource Coordinators, 2013; Figure S1). Thirty-five out of **40** Kv genes were successfully amplified from rat brain tissue. The remaining five channels (Kv1.8, Kv6.2, Kv7.4, Kv11.3, and Kv12.1), which we did not succeed to amplify, were obtained by commercial synthesis (see Methods “IC gene cloning”). All the amplified genes were fully sequenced and verified against the original RefSeqs. With minor exceptions, all cloned sequences were identical to the RefSeqs or contained synonymous variations (Table S1). The amplification process also revealed several new splicing variants (Table S2). As we only focused on the main isoforms (commonly known as isoform 1), these new variants are not included in this study.

We then cloned each amplified Kv gene in a customized mammalian expression vector and transfected them in CHO Flp-In^™^ T-Rex^™^ cells to construct a library of isogenic tetracycline-inducible CHO-Kv stable cell lines (Figure S1). Each Kv cell line was validated before kinetic characterization, as shown for the CHO rKv1.1 cell line as an example (Figure S2). Gene expression screening of the entire library confirmed that each cell line over-expressed a single target Kv gene (Figure S3 and Table S4).

Electrophysiology experiments were performed with an automated patch clamp (APC) robot in a dedicated temperature-controlled room, ensuring temperature stability of the environment (see Methods “Electrophysiology environment”). Different voltage protocols were applied at three different temperatures (15, 25, and 35°C) to probe the kinetic properties of each Kv channel. To allow comparisons across all Kv channels, we used the same ion concentrations in intracellular and extracellular solutions and the same voltage protocols for all Kv channels. Each recorded cell has been assigned a unique ID (Cell ID) for ease of data management. Overall, we recorded from more than **18,700** cells, extracting kinetic features from each recording. **13,401** of these recordings met quality assurance criteria for qualitative analysis and **6,540** met criteria for quantitative analysis (see Methods “QA-QC electrophysiology data”). The raw traces of all cells are publicly available for download from Channelpedia (https://channelpedia.net or https://channelpedia.epfl.ch; Figure 1C).

### Kinetic Characterization of the Rat Kv1.1 Channel

**Figure 2:**
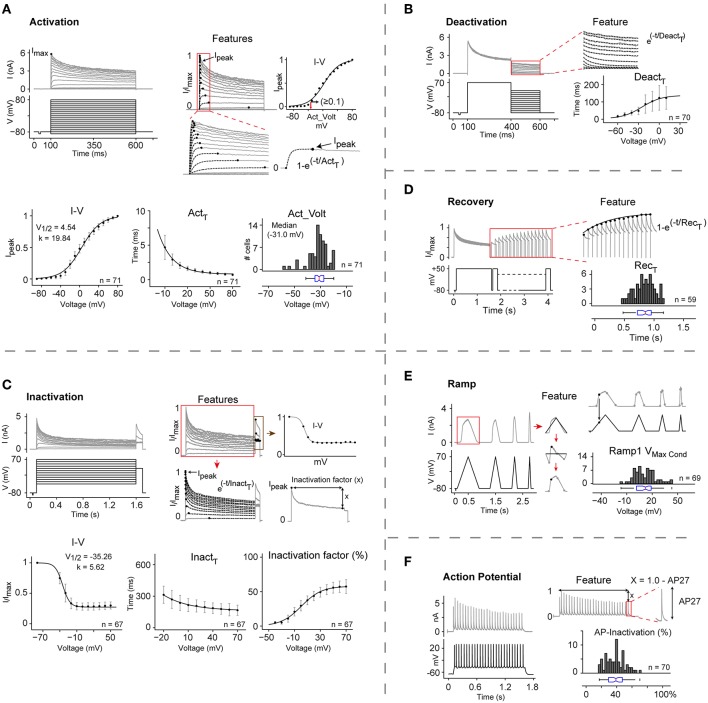
Automated kinetic characterization of CHO rKv1.1 at 25°C. **(A–F)** The kinetic properties of rat Kv1.1 channel in CHO cells are extracted from the current response of six different voltage protocols: Activation, Deactivation, Inactivation, Inactivation recovery, and two *in vivo*-like stimuli, Ramp, and Action potential. Each panel shows the applied voltage stimulus (black traces), representative traces of the current responses (gray traces), region of interest for analysis (red box), and extracted features. For all analysis, current traces are first normalized to overall maximum current (I_max_). All data are presented as means ± S.D. **(A)** I-V curve, activation voltage (Act_Volt), and activation time constant (*Act*_τ_) are extracted for all cells (*n* = 71). Peak current (I_peak_) for each trace is identified and plotted against command voltage to get I-V curve (top right panel). For each cell the voltage where normalized current (I_peak_) exceeds 0.1 in I-V curve is considered as the activation voltage (red arrow). *Act*_τ_ is calculated by fitting single exponential curve from 0 to I_peak_ for each current trace. I_peak_ and *Act*_τ_ from each cell are plotted and fitted with Boltzmann and single exponential function, respectively to get mean I-V curve and mean *Act*_τ_. **(B)** Deactivation time constant (*Deact*_τ_) for each trace is calculated by fitting a single exponential to current response during the second stimulus pulse (400–600 ms) and then plotted against command voltage (*n* = 70). **(C)** Inactivation curve, time constant (*Inact*_τ_), and inactivation factor are features extracted for all cells (*n* = 67). I_peak_ from the second stimulus pulse (red box) are plotted against command voltage to get Inactivation curve. *Inact*_τ_ is calculated by fitting a single exponential to each current trace from I_peak_ to the end of the first stimulus pulse. Inactivation factor (x) is the difference from I_peak_ to the end of the first stimulus pulse. Inactivation I-V, *Inact*_τ_, and Inactivation factors from all cells are averaged and plotted against command voltage and fitted with Boltzmann, single exponential, and Boltzmann function, respectively. **(D)** Inactivation recovery time constant (*Rec*_τ_) is obtained by fitting a single exponential function to the peak current values of the responses to recovery pulses (red box) (*n* = 59). **(E)** Maximum conductance (V_max_Cond_) is calculated on the rising phase of the first Ramp (*n* = 69). **(F)** AP-Inactivation is measured by subtracting last AP (AP27) amplitude from normalized maximum value (1) (*n* = 70). Act_Volt, *Rec*_τ_, V_max_Cond_, and AP-inactivation values for cell population are reported with histograms and box plots.

To illustrate our method, we describe the detailed kinetic characterization of Kv1.1 at 25°C (Figure 2), a well-studied ion channel that has been implicated in several diseases including episodic ataxia, malignant hyperthermia, and hypomagnesaemia (Rajakulendran et al., 2007; van der Wijst et al., 2010; D'Adamo et al., 2014).

Activation properties were analyzed in terms of their I-V relation, time constant for activation (*Act*_τ_), and activation voltage (Act_Volt) (Figure 2A). Normalized peak currents against command voltage (I-V) curves were fitted to a Boltzmann function, yielding V_1/2_ = **4.54** ± 5.87 mV, and slope k = **19.84** ± 1.72 mV. *Act*_τ_ was measured by fitting a single exponential curve to the recorded current trace from start of stimulus to peak current. For voltages from −10 to +80 mV, the value of *Act*_τ_ decreased from **4.68** ± 1.76 to **0.71** ± 0.15 ms. The median activation voltage (Act_Volt)—defined as the voltage where the channel current exceeds 10% of the peak current—was **−31.0** mV (IQR = −34.2 to −27.38).

Deactivation properties were characterized by measuring the tail currents evoked by 200 ms hyperpolarizing stimuli at voltages increasing from −80 to +50 mV in 10 mV steps (Figure 2B). For voltages between −60 and +10 mV, the deactivation time constant (*Deact*_τ_) increased from **14.96** ± 7.32 ms to **120.68** ± 64.9 ms.

Inactivation properties were analyzed in terms of their I-V relation, time constant (*Inact*_τ_) and inactivation factor (Figure 2C), yielding a V_1/2_ value of –**35.26** ± 4.0 mV and slope k = **5.62** ± 2.01 mV. For voltages from −20 mV to +70 mV, *Inact*_τ_ from the peak to the end of the first pulse decreased from **313.25** ± 81.7 ms to **166** ± 48.38 ms. Kv1.1 has been reported to be a non-inactivating or slowly inactivating IC which becomes rapidly inactivating in presence of Kvβ1 subunits (Heinemann et al., 1996; Jow et al., 2004). Our characterization study shows that at +70 mV, this channel inactivates as much as **57.4** ± 10%. However, targeted PCR and full transcriptome screening showed no significant Kvβ1 expression in CHO cells (Figure S4). This led us to conclude that Kv1.1 is indeed an inactivating IC, and that Kvβ1 is not essential for its inactivation.

Recovery from inactivation was analyzed at a recovery potential of −80 mV using 1.5 s conditioning pulse (+50 mV) to induce inactivation, followed by 150 ms test pulses at varying intervals (Figure 2D). The time constant for recovery from inactivation (*Rec*_τ_) was measured by fitting a single exponential curve to the maximum current values during the test pulse, yielding median *Rec*_τ_ =**0.85** s (IQR = 0.72 to 0.95).

To gain more insight into the behavior of Kv1.1, we used two *in vivo*-like stimuli: a slow voltage ramp and a train of 27 action potentials (APs). With the slow ramp we observed maximum conductance at **+12.03** mV (IQR = 1.40 to 18.71; Figure 2E). The response to the train of APs displayed median inactivation of **39**% (IQR = 29 to 47), providing further evidence that Kv1.1 is indeed inactivating (Figure 2F).

### Kinetic Characterization of All Rat Kv Channels at 25 and 15°C

**Figure 3:**
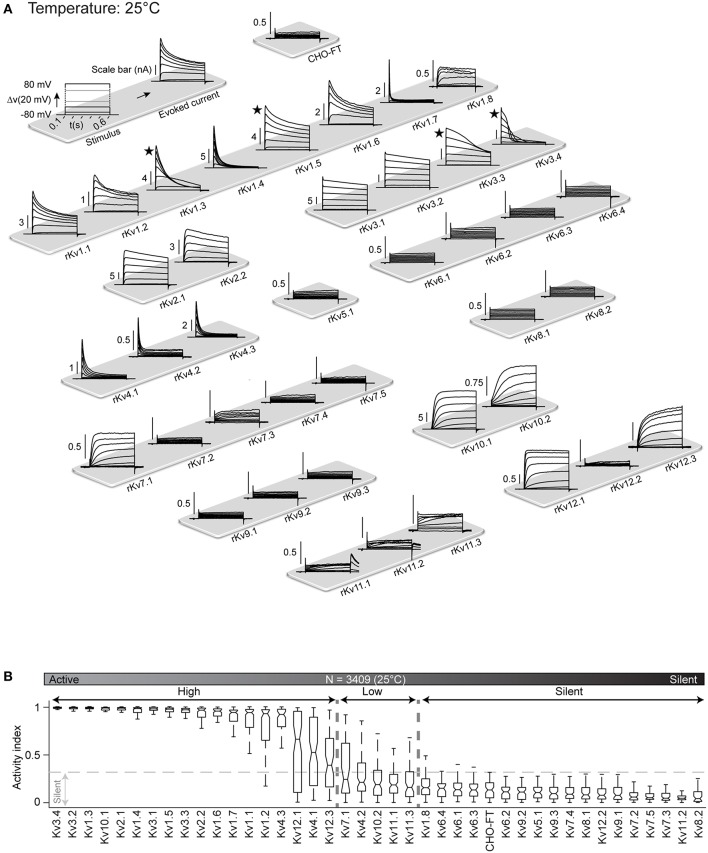
Kv channels kinetic map at 25°C. **(A)** Illustration of activation stimulus with 20 mV steps and evoked current response are shown (top left panel). The amplitude is indicated in nA with scale bar. The non-transfected CHO-FT cell line is shown as control for the background current (top right panel). Representative traces of the typical response to activation stimulus for each Kv channel, recorded at 25°C, is shown. Current traces are sorted by Kv subfamily. ★ indicates a channel with inherent kinetic heterogeneity (see also Figure 6); for these channels, only one from a range of recorded responses is shown. **(B)** Box plots of AI values for each Kv channel at 25°C; ion channels are ordered by their median AI values and categorized as active or silent based on the 0.3 cut-off value for the 3rd quartile of the box plot (*N* = 3,409 cells). Active channels are further divided into highly active or low active based on 0.3 cut-off value for the median value of the box plot.

Each of the Kv cell lines was characterized using the method described above for Kv1.1. Figure 3A shows typical behavior of all the 40 Kv channels in response to the activation protocol. This map provides the first comparative overview of all rat Kv channels kinetics obtained in standardized condition.

As a first analysis, in order to differentiate between active and silent channels we measured voltage-dependent activity with activity index (AI) for each cell. AI is calculated by combining signal to noise ratio (SNR) and non-linearity factor (NLF) (Figure S5). An AI value close to 0 for a cell indicates low voltage dependence denoting an electrically silent channel. Whereas, an AI value close to 1 represents a highly active channel. We produced box plot of AI values for all cells of a Kv cell line and assigned a cut-off value of 0.3 for third quartile (i.e., 75% of the population) to distinguish between active and silent channel. Below this cut-off channels are considered as silent. The remaining channels are considered as active, with a wide range of activity levels. We further used the median values of AI to divide active channels in highly active (median value above 0.3) and low active channels (median value below 0.3).

According to this criterion, at 25°C all members of the Kv5, Kv6, Kv8, and Kv9 families were classified as silent (Figure 3B), which is consistent with the literature; these channels are known to have regulatory effects on active channels (Salinas et al., 1997; Kramer et al., 1998; Bocksteins et al., 2014). Several other channels were silent according to the AI criterion: Kv1.8, Kv7.2, Kv7.3, Kv7.4, Kv7.5, Kv11.2, and Kv12.2. Previous studies reported Kv7.2, Kv7.3, Kv7.4, and Kv7.5 channels as active, showing however that the homomeric channels conduct relatively small current, becoming substantially more active as heteromers (Wang et al., 1998; Nakajo and Kubo, 2008; Gilling et al., 2013), or in association with their accessory subunits KCNE (Barhanin et al., 1996). According to transcriptomic data, KCNE subunits are not expressed in CHO cells (Table S3), that could explain why these channels are silent in this system. Kv11.2 and Kv12.2 have not been formally considered as silent channels but have been shown to have very low conductance (Engeland et al., 1998; Wimmers et al., 2002; Sturm et al., 2005), that is consistent with our data. The remaining 23 Kv channels (Kv1.1-Kv1.7, Kv2.x, Kv3.x, Kv4.x, Kv7.1, Kv10.x, Kv11.1, Kv11.3, Kv12.1, Kv12.3) are all electrically active at 25°C, with wide range of activity levels (Figure 3B). As control, screening for mRNA expression confirmed that all cell lines, for both active and silent channels, correctly expressed the target gene (Figure S3 and Table S4). In addition, analysis of membrane fraction by western-blot on a sample of two active and two silent channels further showed that they had been correctly translocated to the membrane (Figure S6).

The kinetics of all active channels have been already reported (Table 2), but mainly at room temperature which can vary between 18 and 28°C from one study to another. These reports contain many inconsistencies, with agreement only for a few channels (Kv1.4, Kv2.2, Kv4.1-4.3, Kv10.1, Kv10.2). The largest inconsistencies concern Kv1.3, Kv1.5, and Kv3.3 (sometimes described as non-inactivating and sometimes as fast inactivating channels), that could be due to differences in experimental conditions across studies (species, host cell lines, method of expression, temperature, intracellular/extracellular solutions, patch clamp technique etc.). The standardized workflow used in our study makes it possible to compare the kinetic properties for all Kv channels, potentially reconciling these inconsistencies.

In our study at 25°C, most of the active ICs shows more inactivation than previously reported; for example, Kv1.1, Kv1.6, Kv2.1, and Kv2.2, mainly reported as non-inactivating (Table 2), show a consistent inactivating pattern at 25°C (Figure 3A). One possible reason could be that the data reported in literature were acquired at temperatures below 25°C (Table 2). To assess this possible effect of lower temperature, we re-characterized the complete Kv cell line library at 15°C. Indeed, all cell lines show less inactivation at 15°C than at 25°C (Figures S7A, S8A); for example, Kv1.1, Kv1.6, Kv2.1, and Kv2.2 are almost non-inactivating at 15°C providing a closer match to the results in literature (Table 2). This substantial temperature sensitivity points out that measurements of IC kinetics around room temperature do not necessarily reflect their behavior at physiological temperature.

### Kinetic Characterization of All Kv Channels at 35°C

**Figure 4:**
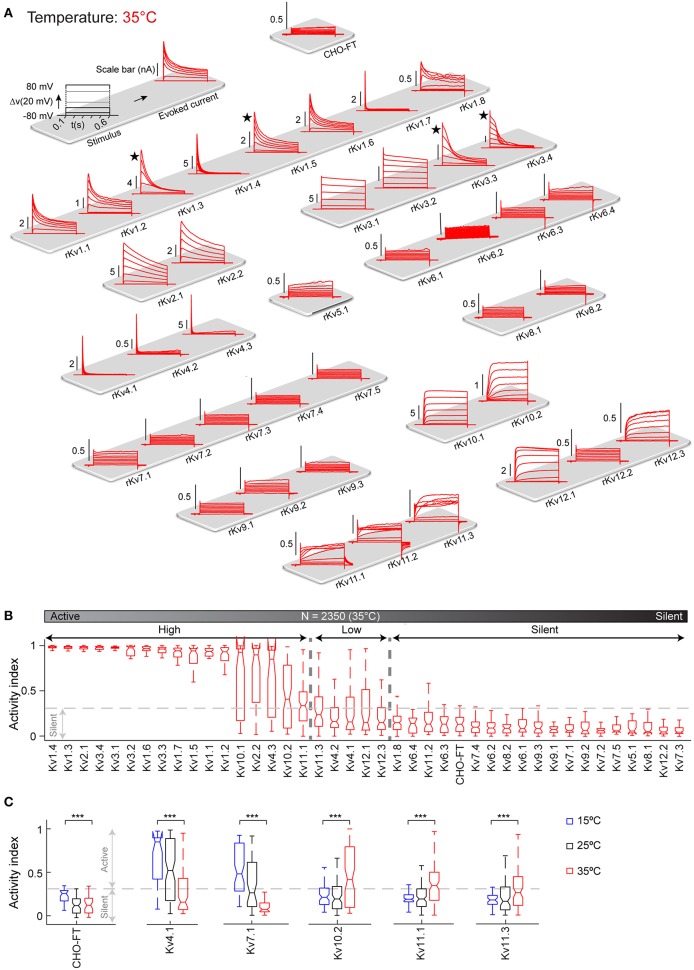
Kv channels kinetic map at 35°C. **(A)** Illustration of activation stimulus with 20 mV steps and evoked current response are shown (top left panel). The amplitude is indicated in nA with scale bar. The non-transfected CHO-FT cell line is shown as control for the background current (top right panel). Representative traces of the typical response to activation stimulus for each Kv channel, recorded at 35°C is shown. Current traces are sorted by Kv subfamily. ★Indicates a channel with inherent kinetic heterogeneity (see also Figure 6); for these channels only one response from the range of recorded responses is shown. **(B)** Box plots of AI values for each ion channel at 35°C; ion channels are ordered by their median AI values and categorized as active or silent based on the 0.3 cut-off value for the 3rd quartile of the box plot (N = 2,350 cells). Active channels are further divided into highly active or low active based on 0.3 cut-off value for the median value of the box plot. **(C)** AI values at 15°C (blue), 25°C (black), and 35°C (red) are plotted for Kv channels that show significant change in activity over temperature. AI values for the non-transfected CHO-FT cell line is plotted as a control (see also Figure S9). ^***^p < 0.001, Student's t-test.

Like other biological processes, Kv channel kinetics is known to become faster with increase in temperature (Table 3). However, there are very few studies on Kv kinetics near physiological temperature (Table 2, right column), and moreover temperature varies between 30 and 39°C depending on the study. To obtain detailed kinetic behavior for all Kv channels near physiological temperature, we screened the complete Kv cell line library at 35°C (Figure 4), close to the maximum limit of the temperature controller in our setup. From a technical perspective, it is more difficult to patch cells at 35°C than at lower temperatures: cell membrane becomes less stable, making it more difficult to achieve and maintain a good seal—likely the reason why so few studies are conducted at higher temperature. In our case, for example, the success rate at 35°C could be as low as ~**15**% compared to ~**80**% at 25 and 15°C. However, the automated patch clamp robot used in our study produced a sufficient number of recordings to compensate for this high failure rate. The map in Figure 4A, provides the first comparative overview of the Kv channel kinetics near physiological temperature (35°C). The kinetics is qualitatively very different from 15, to 25 to 35°C (especially for inactivation; see Figure S7 and Figures 3, 4). Quantitatively, Kv channels activate and inactivate faster at 35°C than at 25°C (Figures S8B,C). In addition, the comparison of AI values of all channels at 15, 25, and 35°C (Figure S9) shows a surprising temperature-dependent effect on the overall level of activity of specific Kv channels. With increases in temperature, Kv10.2, Kv11.1, and Kv11.3 become significantly more active, while Kv4.1 becomes significantly less active (Figure 4C). At 35°C, Kv7.1 becomes even completely silent; all these specific temperature-dependent effects would require further investigations. The voltage-dependent activity of the other Kv channels is not affected by the temperature; as shown in Figure S9, the channels with high activity stay highly active at all three temperatures. In addition, Kv5, Kv6, Kv8, and Kv9 families stay silent at all temperatures. This observation is consistent with the literature where these channels are described to have regulatory effects on active channels (Salinas et al., 1997; Kramer et al., 1998; Bocksteins et al., 2014). As mentioned before, Kv7.2-Kv7.5, Kv11.2, and Kv12.2 homomeric channels are reported in literature to have low conductance at room temperature; our study shows that they remain silent also at 35°C in CHO cells. These channels might require heteromerization or modulatory subunits to be active at physiological temperature.

**Table 3:** Summary of literature reports on the effect of temperature on Kv kinetics, indicating temperatures and reported effects (brief descriptions) with references (detailed references are listed in supplementary document).

**IC**	**Temperatures**	**Reported kinetics**	**References**
Kv1.1	16,3 vs. 26.6°C	Activation time constant decreases with increase in temperature	Moran and Conti, 1995
Shaker B (Kv1.3)	Range of T° (9.9–30.7°C)	N-type inactivation is accelerated with increase in temperature	Meyer and Heinemann, 1997
Kv7.2-7.5	28 vs. 18°C	Faster activation and deactivation kinetics with increase in temperature	Miceli et al., 2009
Kv11.1, Kv11.3	21, 30, and 35°C	Faster activation and inactivation at higher temperature	Mauerhöfer and Bauer, 2016
Kv	Range of T°	Faster activation and inactivation at higher temperature	Pahapill and Schlichter, 1990; Yang and Zheng, 2014

### Comparative Kinetic Properties of Kv Channels at 35°C

**Figure 5:**
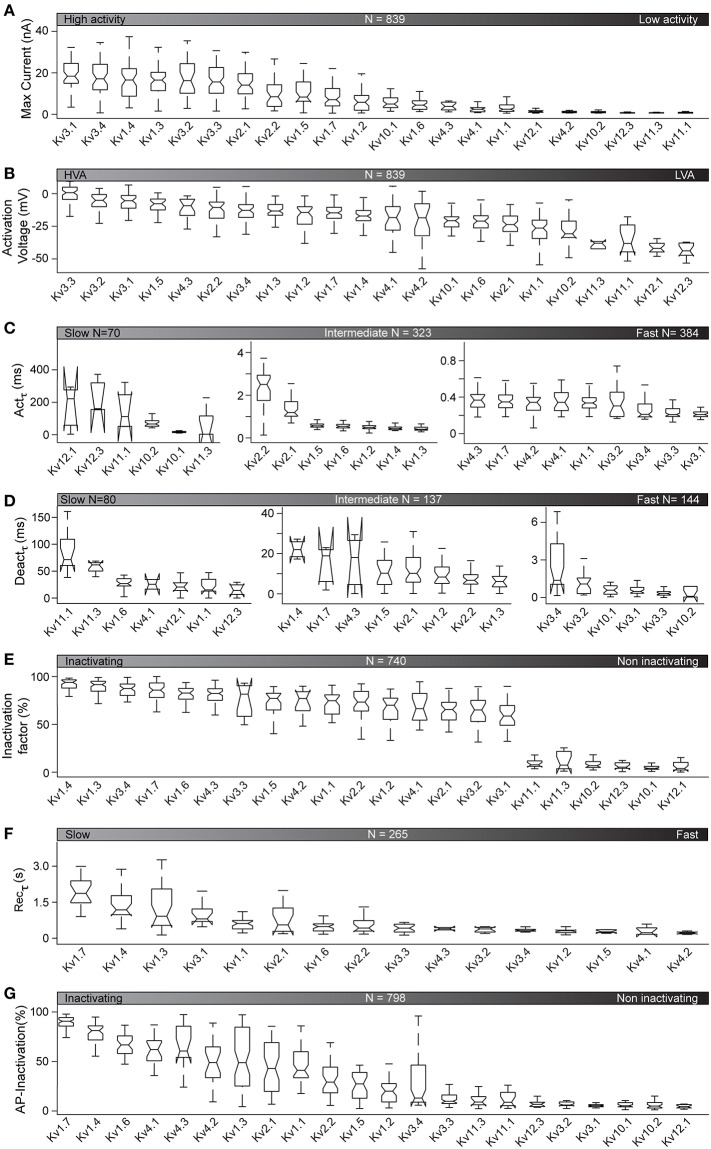
Kinetic properties of active Kv channels at 35°C. Kinetic features, analyzed as illustrated in Figure 2, are plotted for the 22 active Kv channels at 35°C (see Figure 4B). Features are represented as box plots for each ion channel and sorted by their median values. *N* is the total number of cells used for the analysis of each feature (see also Table S5 for detailed cell counts of each group). **(A–G)** Kv responses to the activation stimulus are analyzed to get maximum current response **(A)**, activation voltage (HVA = high voltage activation, LVA = low voltage activation) **(B)**, and activation time constant for V = +50 mV **(C)** (see also Figure S10). Kv responses to the deactivation protocol are analyzed for deactivation time constant for V = −30 mV **(D)**. Responses to the inactivation protocol are analyzed to calculate inactivation factor values at V = +70 mV **(E)**. The 16 inactivating Kv channels (Kv1.4 to Kv3.1) from **(E)** are further analyzed to compare time constants for recovery after inactivation **(F)**. Responses to AP-like stimuli are analyzed for AP-inactivation **(G)**.

Our standardized kinetic characterization of active ICs at 35°C enables us to provide the first comparative analysis of the kinetic properties of the Kv channels near physiological temperature (Figure 5).

The activation protocol used in our characterization captures the opening of an IC in response to a given voltage stimulus. As illustrated in Figure 5A, at 35°C, all active Kv channels show a range of current responses from **0.67** to **18.35** nA (median values). The ICs with the largest current responses (mainly Kv3 family) activate at the highest voltages (Figure 5B) and have the fastest activation rate (Figure 5C, see also Figure S10 in blue). This is consistent with reports of neuronal AP repolarization by currents mediated by Kv3 family (Rudy and McBain, 2001; Labro et al., 2015). In contrast, ICs of the Kv11 and Kv12 families, that show low current responses, activate at lower voltages and have the slowest activation rate (Figures 5A–C and Figure S10 in red). These ICs may play a role in repolarizing the cell membrane at sub-threshold voltages and during sub-threshold oscillations. The behavior of the other Kv ICs lies between these extremes. In general, we observe that channels with high current responses tend to be activated by high voltages and vice-versa (Figure S10 in black).

The Kv4 family is an exception, with all members activating at high voltage but having a low current response (Figure S10 in green).

Deactivation refers to the closing of a channel when a stimulus is removed. The lowest deactivation time constants at 35°C are found for the Kv3 and Kv10 families. The ICs with the highest deactivation time constants (up to **60** ms) belong to Kv11 and Kv12 families (Figure 5D).

Inactivation refers to the closing of a channel during stimulation. Inactivation is an important property of most of the Kv channels, determining how long the channel affects the cell, under suitable activation conditions. There are several conflicting reports about the inactivation properties of Kv channels (Table 2). Our study shows that their kinetic behavior span from highly inactivating (e.g., Kv1.4, Kv1.3, Kv3.4, Kv1.7) to non-inactivating (e.g., Kv12.1, Kv10.1, Kv12.3; Figure 5E). In some cases, we confirm previous reports. For example, we confirm that Kv1.3, Kv1.4, and Kv.4.3 inactivates more than **80**% at 35°C. We also confirm that Kv10.1, Kv10.2, Kv12.1 are truly non-inactivating ICs, maintaining this characteristic at 35°C (Figure 5E). However, in other cases, the extrapolation from 25 to 35°C does not hold. For example, at 35°C, Kv1.6, Kv1.1, and Kv1.2, which are non-inactivating at 15°C, and slowly inactivating at 25°C, actually inactivate by **83**, **74**, and **70**%, respectively at 35°C. Other Kv channels show similar trends. For example, Kv2.1 and Kv2.2, which have been reported in literature to be less inactivating, actually inactivate by **65**% and **75**% respectively, at 35°C.

Apart from conventional inactivation (N, C, P, and U-types), we also observed delayed inactivation for three channels (Kv1.3, Kv3.3, Kv3.4), which is a new type of inactivation recently reported for Kv3.1 (Oliver et al., 2017). This observation is further discussed below. At 35°C, a few Kv channels, particularly Kv1.7, show slow inactivation recovery time (**1.9, 1.18, 0.91**, and **0.81 s** for Kv1.7, Kv1.4, Kv1.3, and Kv3.1, respectively; Figure 5F). All other inactivating ICs require < **0.6 s** (median values) to recover from inactivation.

We also observed inactivation in response to AP-like physiological stimuli. When applying a train of APs, inactivating channels like Kv1.4 and Kv1.7 show up to **80**% inactivation, whereas non-inactivating channels like Kv10.1 and Kv12.1 show no inactivation at all (Figure 5G).

### Inherent Kinetic Heterogeneity and Delayed Inactivation

**Figure 6:**
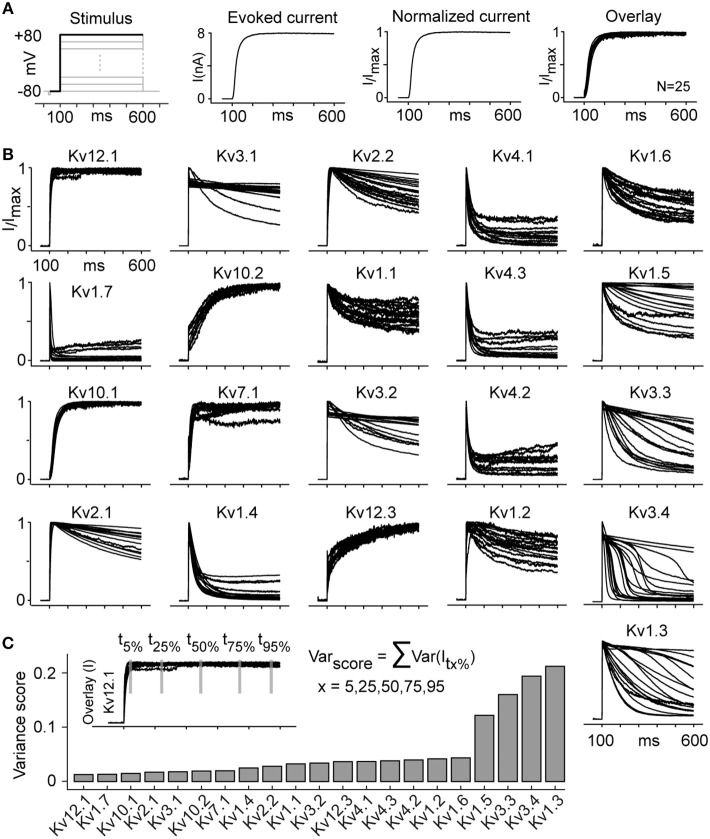
Kv channels kinetic heterogeneity (see also Figure S11). **(A)** The evoked current trace corresponding to the activation stimulus at +80 mV is normalized to maximum current for each cell. The overlay plot presents the normalized currents from 25 cells that statistically represent the whole group (same variance). **(B)** Overlay plots as described in panel A are shown for all active channels at 25°C (Kv11.1 and Kv11.3 are not included due to low current and high noise). The overlay plots visually represent the kinetic heterogeneity. **(C)** Kinetic heterogeneity is quantified by calculating Var_score_, obtained by summing five variances of normalized currents evaluated at five time points for all cells of a given cell line, as shown for Kv12.1 as an example. The channels that show the highest heterogeneity (highest Var_score_) are Kv1.3, Kv1.5, Kv3.3, and Kv3.4. The number of cells (*N*) for each group is listed in Table S5.

The effect of temperature on inactivation explains some inconsistencies in ion channel literature. For example, Kv1.1, Kv1.6, and Kv2.1 are reported as non-inactivating or slow-inactivating depending on the study (Table 2); that we show is a temperature-dependent effect as these channels are all non-inactivating at 15°C but become inactivating at 25°C (Figure S7 and Figure 3). This systematic characterization led to an additional observation: in our study, the majority of the Kv cell lines shows responses that are consistent across different cells from the same cell line. However, in some cases, different cells from the same cell line systematically show different responses (kinetic heterogeneity), despite the fact that they are isogenic (Figures 6A,B). To quantify this kinetic heterogeneity, we have measured the variance in current response for all cells of each cell line, in response to +80 mV command stimulus from activation protocol (Figure 6C). This analysis shows that at 25°C, four ICs, namely Kv1.3, Kv1.5, Kv3.3, Kv3.4, exhibit particularly high kinetic heterogeneity. This phenomenon was observed independently of the temperature or the host cell line (data not shown).

We have verified that the heterogeneity observed is not due to gene mutations (Table S1), cell contamination (Figure S3), recording artifacts (series resistance, intracellular/extracellular composition, extracellular K^+^ accumulation, etc.), cell handling or cell environment. Since the methods used were highly consistent (standardized workflow, isogenic cell lines, recording of ~100 cells for each of these channels), we conclude that the kinetic heterogeneity is an inherent property of specific Kv channels. The underlying mechanism may include transcriptional, post-translational and/or translocational factors and requires further investigation. Except for one previous report about two distinct phenotypes for Kv1.2 cells (Rezazadeh et al., 2007), this is the first report of inherent kinetic heterogeneity in Kv channels. The inherent heterogeneity of Kv1.3, Kv1.5, and Kv3.3 could also explain inconsistencies in previously reported kinetics for these channels. However, the high heterogeneity we observed in the kinetics of Kv3.4, contradicts all previous reports, which describe this channel as a fast inactivating channel (Table 2).

Three of the abovementioned channels (Kv1.3, Kv3.3, Kv3.4) also show striking delayed inactivation, with inactivation starting after a delay that varies from cell to cell—a behavior that differs from classical inactivation patterns (slow, intermediate, or fast inactivation; Figures S11A,B). With longer stimuli, delayed inactivation also appears in two further channels (Kv3.1, Kv3.2) (data not shown). The delayed inactivation was observed at all three temperatures (Figure S11C). To assess if the delayed inactivation is linked with extracellular potassium accumulation, we performed control experiment on Kv3.4 cells. A single voltage pulse of +70 mV was applied to evoke delayed inactivation and then extracellular space was washed with extracellular solution for 90 s to remove potassium accumulation. The delayed inactivation was observed even after multiple washes with extracellular solution (Figure S12A). Moreover, for Kv3.4 cells, Person's linear correlation coefficient between maximum current amplitude and delay in inactivation resulted in a value of 0.12, indicating that the delayed inactivation is not correlated with outward potassium current amplitude (Figures S12B1–B3). Full transcriptome analysis showed no significant expression of Ih, Ca or Na voltage-gated channels, indicating that this delay was not caused by inward current (Figure S12C and Table S3). The mechanism behind this delayed inactivation requires further investigations.

### Voltage-Dependent Q_10_

The Q_10_ has been widely used in biology as a convenient measure of temperature effects. For ion channel modeling, Hodgkin and Huxley (1952) used a constant value of Q_10_ = 3 to model Na^+^ and K^+^ conductances in the squid axon. Since then, other authors have used a fixed Q_10_ value between 1 and 5 in models to estimate the kinetic rates for Kv channels at different temperatures. Our experiments at 15, 25, and 35**°**C, allowed us to measure empirical Q_10_ values for all kinetic parameters, using Van't Hoff's equation:

(1) $$\begin{array}{r} \tau \left(T1\right)=\tau {\left(T\right)}^{*}Q_{10}^{\left(\frac{T-T1}{10}\right)} \end{array}$$

where *T* and *T*1 are the temperatures (in °C) at which experiments were carried out, and τ(*T*) and τ(*T*1) are the time constants.

**Figure 7:**
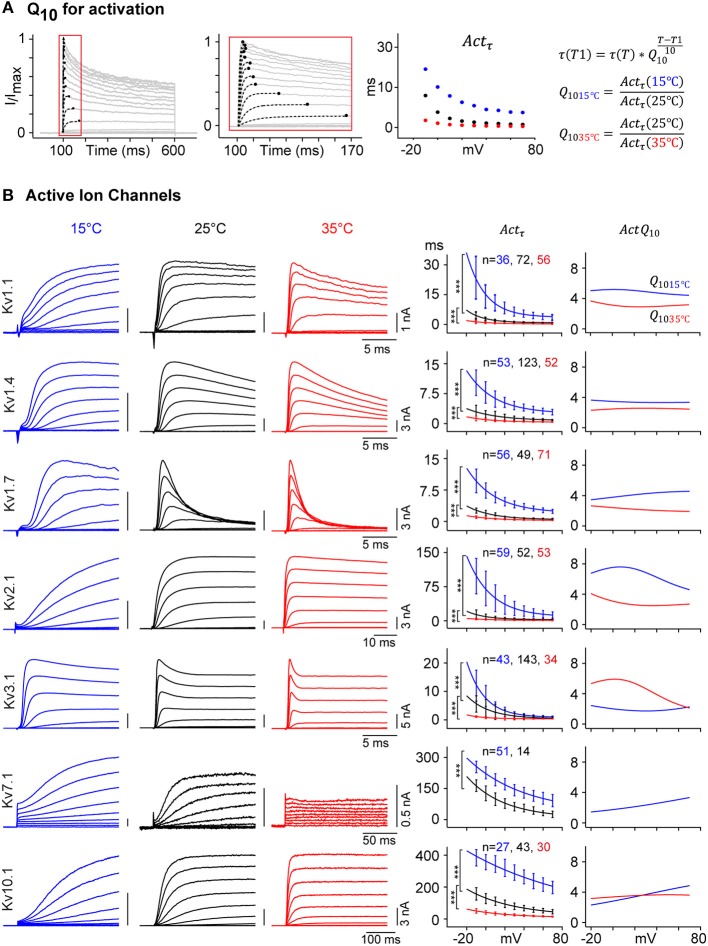
Temperature dependence of Kv channel kinetics. **(A)** Voltage-dependent *Act*_τ_ is measured as shown in Figure 2. Q_10_ of *Act*_τ_ is calculated for 15 and 35°C, with 25°C as a reference. **(B)** Representative current traces for selected Kv channels in response to activation protocol at 15°C (blue), 25°C (black), and 35°C (red) are shown (left panels), the amplitude in nA and time in ms are indicated with scale bars. Single exponential curve is fitted to the median value of *Act*_τ_ for each of the three temperatures (middle panel, solid line; ^***^p-value < 1e-5, Student's *t*-test). For each selected Kv channel, Q_10_ values for 15°C (blue) and 35°C (red) are plotted against command voltage (right panel). For a given Kv channel, Q_10_ value is different for different temperature range and vary between 2 and 8 across different voltages. For Kv7.1, which becomes electrically silent at 35°C, *Act*_τ_ and Q_10_ values for 35°C are not plotted. Error bars are ± S.D.

As an example, Figure 7 shows Q_10_ for *Act*_τ_ for selected Kv channels with very different activation profiles. *Act*_τ_ was obtained by fitting the current traces to a single exponential and plotted against command voltage (Figure 7A). Q_10_ for 15 and 35°C were calculated considering 25°C as the base temperature, the usual temperature used for ion channel studies:

(2) $$\begin{array}{r} Q_{10_{15c}}=\;\frac{\tau \left(15^{{}^{\circ}}C\right)}{\tau \left(25^{{}^{\circ}}C\right)}and\;Q_{10_{35c}}=\;\frac{\tau \left(25^{{}^{\circ}}C\right)}{\tau \left(35^{{}^{\circ}}C\right)} \end{array}$$

Applying Equation (2) to our empirical data for all active Kv channels yields values that vary not only as a function of temperature but also of voltage (Figure 7B). For example, Kv1.1 has a *Q*_1_0__15*c*__ value around 5 and a *Q*_1_0__35*c*__ value between 3 and 4, varying with voltages. Data for other IC show that Q_10_ can be lower than 2 (ex: Kv7.1 at 15°C) or as high as 8 (e.g., Kv2.1 at 15°C) depending on voltages and temperature range. Our detailed kinetic characterization of all Kv channels provides data to calculate accurate temperature and voltage-dependent Q_10_ for all kinetic parameters.

### Voltage-Dependent Q_10_ Hodgkin-Huxley Model

One case where extrapolating across temperatures may lead to inaccurate results is the implementation of IC kinetics in computational models. Neuronal models often use the Hodgkin-Huxley (H-H) formalism to describe IC conductances. In this section, we describe a revised model-fitting procedure, which accurately incorporates voltage dependence in temperature sensitivity. We use Kv1.1 as an example.

Briefly, current traces from individual cells were fitted to single activation and inactivation gates in the H-H formulation, and model parameters were extracted for each cell. The medians of these parameters were fitted with Q_10_ functions.

The net transmembrane current flow, *I*_*Kv*1.1_, and the conductance *g*_*Kv*1.1_were computed using Equations (3) and (4):

(3) $$\begin{array}{r} I_{Kv1.1}=g_{Kv1.1}\left(V_{m}-E_{k}\right) \end{array}$$

(4) $$\begin{array}{r} g_{Kv1.1}=\frac{I_{Kv1.1}}{\left(V_{m}-E_{k}\right)}=\bar{g}m^{p}h^{q} \end{array}$$

Where *V*_*m*_ is the membrane potential, and *E*_*k*_ denotes the Nernst potential. Variable $\bar{g}$ scales for maximum channel conductance. *m* and *h* represent the proportion of open activation and inactivation gates, respectively; *p* and *q* are the numbers of independent gates required to account for the observed time course of activation and inactivation. Since single gates are used both for activation and for inactivation, *p* = *q* = *1*. Gating variables are modeled as a first-order kinetic process

(5) $$\begin{array}{r} \frac{dm}{dt}=\frac{m_{\infty }-\;m}{m_{\tau }} \end{array}$$

(6) $$\begin{array}{r} \frac{dh}{dt}=\frac{h_{\infty }-\;h}{h_{\tau }} \end{array}$$

Where *m*_∞_, *h*_∞_ represent the voltage-dependent steady state and *m*_τ_, *h*_τ_ are the voltage-dependent time constants for activation and inactivation gates.

**Figure 8:**
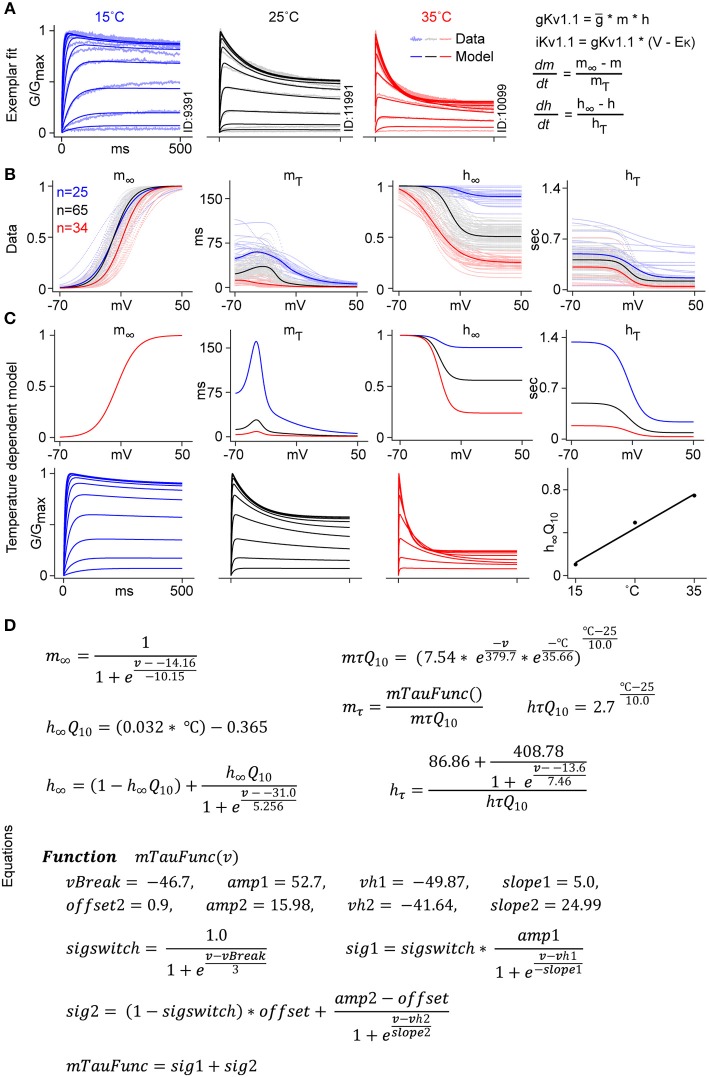
Kv1.1 temperature-dependent H-H modeling. **(A)** Kv1.1 CHO cells recorded from three different temperatures are used for H-H model fitting. An example of the fit to normalized conductance for cells recorded at three different temperatures, 15°C (blue), 25°C (black), and 35°C (red) is shown (see Methods “H-H model fitting”). **(B)** Voltage-dependent *m*_∞_, *h*_∞_, *m*_τ_, *h*_τ_ parameters are plotted (in dots) for all fitted cells. Median values, fitted with a smooth function, are represented with solid lines. **(C)** Voltage dependence of the *m*_∞_ parameter is approximated with a single Boltzmann function for all three temperatures. *m*_τ_ is fitted with voltage and temperature-dependent Q_10_. The steady state value of *h*_∞_ is computed using the temperature-dependent linear function *h*_∞_*Q*_10_. *h*_τ_ is fitted to a Boltzmann curve with a constant Q_10_ value of 2.7. **(D)** Equations used to fit the temperature-dependent Kv1.1 model.

The free parameters in Equations (3)–(6) were fitted to the normalized conductance for each cell (Figure 8A for a sample fit). The fitted parameter values for all cells were plotted and median parameter values for each temperature were calculated (Figure 8B).

The *m*_∞_ function was considered temperature independent, thus a single Boltzmann function was used for all three temperatures. The remaining three parameters, *h*_∞_, *m*_τ_ *and h*_τ_, were fitted with three different Q_10_ functions. For the steady state inactivation function *h*_∞_, we used a temperature-dependent linear function *h*_∞_*Q*_10_. *m*_τ_ curves were fitted using a double exponential function of voltage and temperature, *mτQ*_10_. The *hτQ*_10_ function was represented by a constant value of 2.7 (Figure 8D). Figure 8C shows the final plots for each gating variable, and the normalized conductance plot for each temperature.

The main difference between our approach and regular H-H modeling was that, instead of using a constant value for Q_10_ (usually between 2 and 3), we used a voltage and temperature-dependent function for *m*_τ_ and a temperature-dependent linear function for *h*_∞_. With these changes, we were able to fit the temperature sensitivity of Kv1.1 to the experimental data, which was not possible with the standard model. The resulting revised H-H model can thus be used to extrapolate kinetics from the temperature at which the channel was studied to physiological temperatures.

### Kv Channel Kinetics Across Host Cell Lines and Species

**Figure 9:**
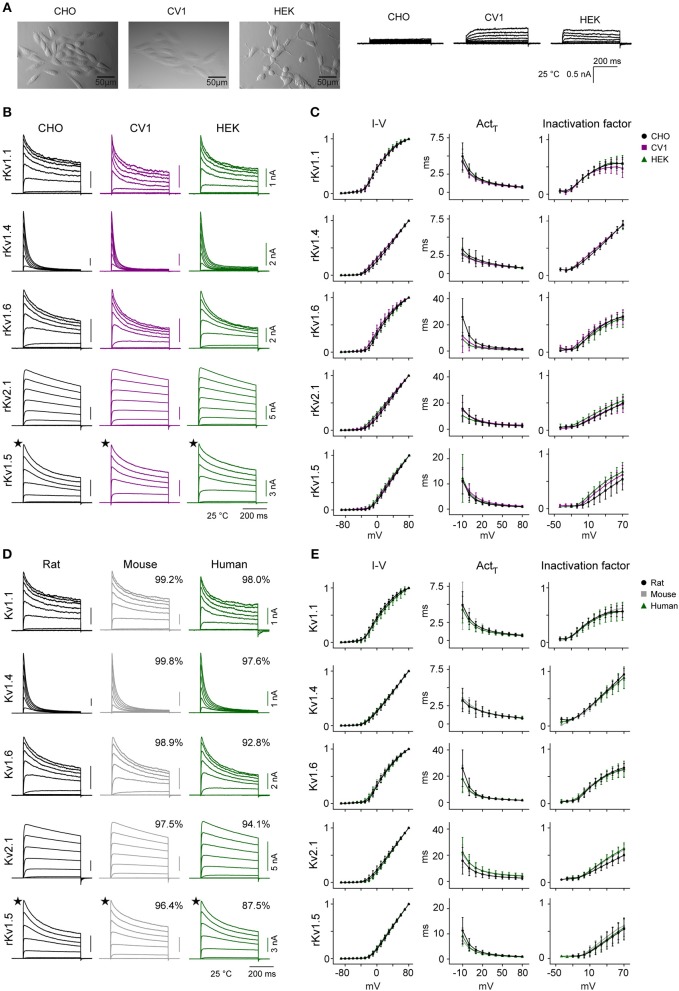
Kv channel kinetics across host cell lines and species at 25°C. **(A–C)** Comparison of Kv kinetics across three mammalian host cell lines. **(A)** Pictures showing the morphology of the three adherent host cell lines (CHO, HEK, and CV1) used in the study, with their corresponding endogenous outward currents in response to the activation protocol at 25°C. **(B)** Representative current traces from rat Kv1.1, Kv1.4, Kv1.6, Kv2.1, and Kv1.5 expressed in the three different cell lines, in response to activation protocol at 25°C. **(C)** Median values for three kinetic features (I-V curve, activation time constant, and inactivation factor calculated as explained in Figure 2) obtained from CHO, CV1, and HEK host cell lines for each Kv channel, overlaid for comparison. **(D,E)** Comparison of Kv kinetics across three species. **(D)** Representative current traces for rat, mouse, and human Kv1.1, Kv1.4, Kv1.6, Kv2.1, and Kv1.5 expressed in CHO cells, in response to activation protocol at 25°C. The percentage of homology compared to rat are shown in box above current traces (see also Table 4). **(E)** Median values for three kinetic features (I-V curve, activation time constant, and inactivation factor calculated as explained in Figure 2) obtained from rat, mouse, and human genes of each Kv channel, overlaid for comparison. Error bars are ± S.D. The amplitude in nA and time in ms are indicated with scale bars. ★: For Kv1.5, only one representative trace from a range of responses is shown. The number of cells (*N*) for each group is listed in Table S5.

Previous studies of IC kinetics used many different host cell lines, with different origins, morphologies, intracellular environments, cell handling etc. These differences may also have contributed to all those conflicting reports on the kinetics of specific ICs. To verify possible dependencies on the specific cell line (i.e., CHO) used in our study, we used our standardized workflow to characterize the kinetics of five significantly different active Kv channels (Kv1.1, Kv1.4, Kv1.5, Kv1.6, and Kv2.1) in two additional cell lines (CV1 and HEK). The three cell lines are all adherents and have different morphologies (Figure 9A). In contrast with CHO cells, CV1 and HEK cells both generate small outward currents, possibly due to higher levels of endogenous IC expression (Table S3). Our analysis of three kinetic features (I-V, *Act*_τ_, Inactivation factor) shows that, except for Kv1.5's inactivation factor, the kinetic behaviors of the ICs are largely consistent across the three cell lines at 25°C (Figures 9B,C). Furthermore, the kinetics are comparable also at 35°C for Kv1.1, Kv1.4, and Kv2.1 (Figures S13A,B).

IC genes are highly conserved across species (Siepel et al., 2005); 36 out of 40 rat Kv channels have >**97**% homology with mouse orthologs (Table 4) and even the least homologous channels (Kv6.4, Kv8.2) share >**95**% homology in their protein sequence. Similarly, 23 out of 40 rat Kv channels show >**95**% homology with human orthologs, and 36 out of 40 share >**90**% homology. However, some channels do show differences in the length of their N-terminus and/or C-terminus regions (Table 4). What is not known is whether and how these small differences in sequences translate into a difference in Kv channels kinetics. To address this question, we cloned the mouse and human versions of the same five active channels previously used for host cells comparison, and we characterized them in CHO cells at 25°C (Figures 9D,E) and at 35°C for Kv1.1, Kv1.4, and Kv2.1 (Figures S13C,D). Among these five characterized channels, Kv1.1, Kv1.4, Kv1.6, and Kv2.1 have high homology between species, whereas Kv1.5 was chosen as an example of an active channel with one of the lowest homology across species. We detected only subtle quantitative differences in some kinetic parameters across species both at 25 and 35°C (Figures 9D,E and Figures S13C,D). For example at 25°C, we measured a slightly slower activation time constant for human Kv2.1 compared to rat Kv2.1, as previously reported (Ju et al., 2003; Figure 9E); however, the difference no longer remains significant at 35°C (Figure S13D). We conclude that the behavior of these five Kv channels is relatively conserved across the three species.

**Table 4:** Protein sequences of rat, mouse, and human Kv channels were aligned and analyzed for homology using the ClustalW method alignment function in the DNAstar software.

**Ion channel**	**% Homology with rat**		**Main inter-species size differences**
	**Mouse**	**Human**	
Kv1.1	99.2	98	
Kv1.2	100	99.4	
Kv1.3	99.6	97.3	Human, 52 aa longer N-tail
Kv1.4	99.8	97.6	
Kv1.5	96.4	87.5^*^	Human, 11 aa insertion in N-ter region
Kv1.6	98.9	92.8	
Kv1.7	98.4	93.6	Human, 32 aa shorter N-tail
Kv1.8	98	92.4	
Kv2.1	97.5	94.1	
Kv2.2	97.7	93.7	
Kv3.1	100	99.7	
Kv3.2	96.4	97.4	Mouse, different, and 22 aa longer N-tail
Kv3.3	97.7	91.4	Human, different, and 11 aa shorter N-tail
Kv3.4	99.4	95	Human, different, and 11 aa longer N-tail
Kv4.1	98.2	95.2	
Kv4.2	99.7	99	
Kv4.3iso2	99.5	99.4	Mouse, 19 aa insertion in C-ter region
Kv5.1	99.2	96.6	Human, 12 aa shorter N-tail
Kv6.1	98.6	92.6	
Kv6.2	97.3	90	Human, different, and 14 aa shorter N-tail
Kv6.3	99.1	96.8	
Kv6.4	95.9	81.1^*^	Human, 11 aa insertion in C-ter region
Kv7.1	97.5	90.3	Human, 6 aa insertion in C-ter region
Kv7.2	99.3	95.5	
Kv7.3	99.7	95.3	
Kv7.4	99.3	96.7	
Kv7.5	98.4	95.3	Human, 19 aa deletion in N-ter region
Kv8.1	99.2	95.6	
Kv8.2	95	83.5^*^	Human, shorter N-ter region, and S3-S4 loop
Kv9.1	97.8	90.9	Human, 31 aa longer N-tail
Kv9.2	99.6	98.1	
Kv9.3	98.8	95.5	
Kv10.1	98.7	97.6	
Kv10.2	99.4	98.3	
Kv11.1	99	95.9	
Kv11.2	97	90.2	Human, 44 aa insertion in C-ter region
Kv11.3	98.2	94.7	
Kv12.1	97.6	92.6	
Kv12.2	98.7	95.9	Difference in length of the S5–S6 region
Kv12.3	97.1	89.9^*^	

*Homology of the mouse and human sequences with respect to the rat sequence are indicated as a percentage, along with other significant interspecies differences in sequence length*.

* *Indicates sequences with <90% homology*.

### A Data Resource of Ion Channel Kinetics

To facilitate sharing and further validation of our results, we have published all raw and processed data from our study as a wiki-like web resource (https://channelpedia.net or https://channelpedia.epfl.ch), freely available for academic use. Each cell has been assigned a unique cell ID, which is appended to the data file name. The raw data file provides the recorded currents for each repetition of the voltage protocols and a description of the stimuli applied. The analyzed data file contains features extracted from recordings of each repetition of each protocol. The experimental and analyzed data for each Kv IC are made available in Neurodata without Borders (NWB) format under “Experimental Data” section on the Channelpedia website. NWB is a new format designed to promote data standardization and sharing (Teeters et al., 2015). NWB is based on the HDF5 file format, thus can be viewed with any HDF5 viewer. The raw data, stimulus and metadata are stored under “acquisition/timeseries,” “stimulus/presentation,” and “general,” respectively. The data can be accessed via a web interface or a REST API. Over 1 million recording traces from over **18,700** cells are available and can be downloaded for academic use.

## Discussion

In this study, we produced a standardized map of the kinetics of the homomeric form of all members of the Kv family, at 15, 25, and 35°C. The map includes all the channels, also those not previously studied or underrepresented in the literature (Table 1). The data we provide near physiological temperature were not available for most of the Kv family members. We show that under controlled conditions, these channels kinetics is reproducible and mostly conserved across host cell lines and species. We find that for many Kv channels, the kinetics near physiological temperature not only differ quantitatively as expected from temperature-dependent effects, but also qualitatively. We find that for all Kv channels, Q_10_ is not only temperature-dependent, but also voltage-dependent. To allow extrapolation from the large number of low temperature kinetics available in the literature to kinetics at physiological temperatures, we provide the data on the voltage-dependent Q_10_ for each channel and a revised Hodgkin-Huxley model to capture the channel kinetics accurately. We found a novel form of delayed inactivation and inherent heterogeneity in kinetics for some Kv members, behavior that was likely interpreted as artifacts in previous studies. We suggest that different experimental conditions, non-trivial temperature dependencies and inherent heterogeneity can account for most of the inconsistencies in kinetics reported in the ion channel literature. The full dataset of over one million traces is available as a publicly accessible resource (https://channelpedia.net or https://channelpedia.epfl.ch).

### Reference Kinetic Dataset for Kv Channels

A vast amount of data on Kv channel kinetics is available in the literature. The lack of consistency limits the value of these data and prevents a consensus on ion channel kinetics. The reference dataset we provide was generated using a standardized heterologous system with isogenic cell lines and an inducible-expression system to achieve a high level of reproducibility and reliability.

Our system, however, does have limitations. In particular, levels of expression are probably much higher than *in vivo*, and it is not possible to control levels of expression across cells. We also cannot account for possible effects of transcriptional regulation in different cells of the body at different stages of development.

The need of special recording solutions for APC experiments is another limitation of this study. Specific fluoride-based intracellular solution (ICS) (50 mM KCl, 10 mM NaCl, 20 mM EGTA, 60 mM KF, 10 mM Hepes), extracellular solution (ECS) (140 mM NaCl, 4 mM KCl, 1 mM MgCl_2_, 2 mM CaCl_2_, 5 mM D-glucose monohydrate, 10 mM Hepes) and seal enhancer solution (SES) (80 mM NaCl, 3 mM KCl, 10 mM MgCl_2_, 35 mM CaCl_2_, 10 mM Hepes) were used for APC experiments. In ICS, instead of usual 2–10 mM, 20 mM EGTA was used as per specifications of APC robot to keep cells longer in whole cell configuration and to maintain constant access resistance. However, we verified that reducing EGTA to 10 or 0 mM did not affect the kinetics of the selected Kv ion channels (data not shown). Inclusion of fluoride in ICS has been shown to improve patch clamp seal quality and to stabilize the cell membrane, resulting in longer and more stable patch clamp recordings (Kostyuk et al., 1975). While the effect of internal fluoride on Kv channel kinetics has been reported, directly (Adams and Oxford, 1983) or through PIP2 (Rodriguez-Menchaca et al., 2012), there are other reports claiming a lack of side effects on Kv channel kinetics (Clay, 1988; López-López et al., 1993; Zeng et al., 2013). Along with fluoride in ICS, SES with high Ca^2+^ and Mg^2+^ was also applied extracellularly to achieve the giga seal. In our experience, without fluoride in ICS and high Ca^2+^ extracellularly, the APC robot could not form the giga seal. The SES was washed out with ECS after reaching whole cell configuration as well as between each repetition of voltage protocols. We observed that high Ca^2+^ and Mg^2+^ in SES did cause membrane potential oscillation artifacts for Kv11.1 and Kv11.3 cell lines, but it disappeared after washing with ECS solution (Figure S14). In conclusion, fluoride in ICS and high Ca^2+^ in SES were necessary to achieve giga seal in our APC experiments. Previous studies have found no significant differences in channel kinetics between manual and automated patch clamp system (Li et al., 2017). In addition, we did not find any difference in Kv2.1 kinetics when comparing with data obtained with another automated patch clamp robot (Fejtl et al., 2007) that worked with physiological ICS, ECS, and without the need of SES solution (data not shown). Despite these limitations, the solutions used in this study still contain physiological levels of sodium and potassium concentration to better reflect *in vivo* ion composition, and not bi-ionic high potassium (K^+^) solution as used in many previous studies on potassium channels.

Currently, the reference dataset we provide, contains the main isoform of each Kv channel and does not include other known splicing variants. It is known that Kv channel genes, such as those involved in the immune system, are highly prone to alternative splicing (Lipscombe, 2005) that substantially increases the repertoire of Kv channels proteins. For example, the 40 known human Kv genes have 73 validated, and 113 predicted splicing isoforms. Our study additionally identified several unreported splicing variants (Table S2), showing that the full repertoire is yet to be mapped. The investigation of the kinetic activity of the different variants using our standardized system would also be of high value.

### Kv Kinetics Near Physiological Temperature

Data on Kv kinetics near physiological temperature were not available for most of the Kv members (Table 2). This is the first complete map of Kv channels kinetics at 35°C. The qualitative differences we found at 35°C clearly suggests the need of a reassessment of previous functional characterizations of ICs. For example, Kv1.1 is generally considered to be a non-inactivating channel, which only inactivates in presence of Kvß1 or Kvß3 subunits (Table 2). We show that Kv1.1 actually inactivates strongly at 35°C, even in absence of these subunits in CHO cells (Figure S4). Similarly, Kv1.2 and Kv1.6, reported to become fast inactivating in presence of Kvß1 or Kvß3 subunits (Heinemann et al., 1996; Bähring et al., 2004), actually are strongly inactivating at 35°C even in absence of these subunits. As another example, we show that Kv7.1, one of the most studied ion channel, reported as active at room temperature and also at 35°C (Loussouarn et al., 1997), is actually only active at low temperatures and becomes silent at 35°C in CHO cells. Furthermore, it is generally accepted that KCNE1 subunit (mink) is required for proper functioning of Kv7.1 (Sanguinetti et al., 1996; Aromolaran et al., 2014); the KCNE1 subunit might make Kv7.1 more active, but it is not required to form a functional channel, at least at low temperature. These examples illustrate how understanding the homomeric channel kinetics across temperatures is essential to correctly interpreting the effects of the several levels of regulation, such as heteromerization, accessory subunit associations or effects of post translational modifications (e.g., phosphorylation, glycosylation).

### Implications for Ion Channel Modeling

The biophysical map and the reference dataset provide data for both improving the accuracy of genetically specified ion channel models and for cellular models (e.g., neurons and heart cells) which often use generic conductances (e.g., A-type channels, delayed rectifier channels) that do not capture the diversity of kinetics present within these generic classes. For example, Kv1.1 and Kv1.7 both belong to the Kv1 family but have completely different time constants for activation and inactivation. Generic IC models are unable to exploit the growing volume of data from single cell transcriptomics, which makes it possible to identify the full spectrum of IC genes expressed by specific cell types.

The data we publish will enable researchers to develop improved models for all the Kv channels. Particularly important is the availability of temperature and voltage-dependent Q_10_ for all kinetic parameters of Kv channels. Patch clamp experiments on cell lines at physiological temperature are difficult and hence majority of the experiments in the future might continue to be performed at lower temperatures. The availability of Q_10_ values from our data makes it possible to interpret those data in the context of physiological temperature. This prospect is very important for ongoing brain modeling programs such as the Blue Brain Project and the Allen Institute for Brain Sciences as well as for initiatives which develop on their work, such as the Human Brain Project. In addition, since specific receptors affect different subsets of ICs in different cells, the new data open the prospect of modeling the effects of neuromodulators on specific neuron types (Nicoll, 1988; Khorkova and Golowasch, 2007).

### Inherent Heterogeneity and Delayed Inactivation

Our standardized kinetic characterization has revealed inherent kinetic heterogeneity in four Kv channels, namely Kv1.3, Kv1.5, Kv3.3, and Kv3.4, three of which also show a novel form of delayed inactivation. This inherent heterogeneity is present in isogenic cell lines that have not been manipulated in any way. The large number of recordings in standardized condition and screening of all Kv channels, allowed us to conclude that it is a truly inherent property. To our knowledge, the only previous study that mentions inherent heterogeneity is a report of two distinct phenotypes for rat Kv1.2 expressed in CHO cells after transient transfection (Rezazadeh et al., 2007). Specifically, the authors describe two gating phenotypes (fast and slow) influenced by a cytoplasmic regulator. Modulation in IC kinetics have been reported to occur upon glycosylation (Brooks et al., 2006; Noma et al., 2009), phosphorylation (Desai et al., 2008; Ritter et al., 2012), oxidation (Hoshi and Heinemann, 2001), or by co-expression of beta-subunits (Heinemann et al., 1996; Bähring et al., 2004) or other binding proteins (Pongs and Schwarz, 2010). We suggest that especially these four channels (Kv1.3, Kv1.5, Kv3.3, and Kv3.4) are susceptible to abovementioned modifications. The inherent heterogeneity we described may also explain some of the conflicting reports present in the literature (Table 2).

The delayed inactivation has been observed only for human Kv3.1, and only at high (>35°C) temperatures (Oliver et al., 2017). The mechanism underlying this novel form of delayed inactivation is unknown and would require further investigation.

### Conserved Kinetics Across Species

Using a sample set of five Kv channels with different kinetics and different degree of homology, we found only subtle differences in some kinetics parameters between mouse, rat and human channel kinetics. Compared to the strong temperature-dependent effect on ion channel kinetics, the inter-species differences we observed are minor. According to these data on a sample set and the high degree of homology of ion channels (Table 4), we postulate that data on ion channels kinetics could be generalized over species. However, this has to be done with caution as even subtle differences observed in kinetics for a single ion channel type could have a strong impact over a neuronal network.

Our observation is consistent with the fact that transmembrane regions S4 to S6, believed to be primarily responsible for channel kinetics, are structurally almost identical across the three species. Nearly all the interspecies differences in the Kv channels sequences concern the N-terminus and C-terminus regions and the S1–S2 loop. The N and C-terminus contain protein binding motifs that are involved in the distribution of IC through the cell (Lai and Jan, 2006; Vacher et al., 2008; Duménieu et al., 2017). The N-terminus region is also involved in tetramerization and N-type inactivation via the “ball and chain” mechanism (Bezanilla and Armstrong, 1977). Hence significant differences in the length of N-terminus or C-terminus regions could affect channels kinetics between different species. For example, human Kv1.7, that has a 32 amino acids shorter N-terminus, has been reported as slow-inactivating while the longer versions in mouse and rat are reported as fast inactivating (Finol-Urdaneta et al., 2006, 2012). Such channels with significant difference in the length of their N-terminus and/or C-terminus regions (Table 4) would require separate studies to clarify the effect of such possible interspecies differences on their kinetics.

### Implications for Drug Discovery

The methods we have established to create the kinetic map of Kv channels can be used to systematically screen drug candidates for potentially positive or deleterious effects on their kinetics. They can also be used to test the effects of targeted mutations and the ability of drugs to reverse their effects. Such studies have the potential to improve our understanding of disease mechanisms, and have obvious applications in drug discovery. In a longer-term perspective, detailed neuron models incorporating genetically specified ion channels could allow simulation of channelopathies at the cellular and network level.

### Future Outlook

For the first time in ion channel research, electrophysiology data from a complete family of ICs have been obtained and made publicly available. We have developed Channelpedia as an online tool for data sharing and we believe that the availability and the ease of data access on this website may encourage other researchers to make their electrophysiology data available through Channelpedia or similar platforms. The availability of the new dataset allows us to investigate links between IC genetics, structure, and biophysics. For example, preliminary results from pairwise alignment of the sequences for the S1–S2 loops, reveal a genetic basis for at least two big groups—inactivating and non-inactivating ICs (data not shown). Large-scale bioinformatics studies aimed at establishing genotype-phenotype correlations are now possible. A key issue for future works is to unravel the mechanism for inherent heterogeneity of IC kinetics.

Homotetrameric Kv channels consist of four identical alpha (α)-subunits, but additional diversity is introduced when different α-subunits combine to form heterotetramers (Dodson et al., 2002; Hadley et al., 2003; Zhang et al., 2016). The function of homotetramers and heterotetramers is further modulated by a broad range of auxiliary (signaling/scaffolding) proteins (Levitan, 2006; Li et al., 2006), including Kvβ-subunits (Pongs and Schwarz, 2010), chaperone proteins (HSP90, HSP70) (Ficker et al., 2003), and other regulatory protein families like K^+^ channel-Interacting proteins (KChIPs), K^+^ channel-associated proteins (KChAP), 14-3-3 proteins, A-kinase-anchoring proteins (AKAP) (Zhang et al., 2016), Dipeptidyl aminopeptidase-like protein (DPP), and KCNE-encoded proteins (Coetzee et al., 1999). Among all possible combinations for heterotetramers and auxiliary subunits, some have been shown to have modulatory effects on kinetic properties, trafficking, permeation, and stability of Kv channels. However, there are also contradictory results on the role of auxiliary subunits; for example on the role of Kvβ3 on Kv1.1 activity ((Bähring et al., 2004) shows that co-expression of Kvβ3 modulates the inactivation of Kv1.1 whereas (Majumder et al., 1995) shows no effect of Kvβ3 on Kv1.1 kinetics) or on the effect of KCNE1(Mink) on Kv7.1 activity ((Bett et al., 2006) shows that Mink modulates KCNQ1 kinetic whereas (Sanguinetti et al., 1996) shows that Mink is necessary for KCNQ1 to be active). The next layers of the kinetic map for the Kv family are the heterotetramers map (kinetics of viable combinations of α-subunits), the β-subunits map (kinetics when bound to β-subunits), and the neuromodulatory map (kinetics for all forms of modulation). The biggest challenge in building these maps is to identify principles for biologically viable combinations of subunits and modulation since all theoretical combinations cannot be mapped. Knowledge of the detailed kinetics of the homotetrameric channels with their structural and genetic correlates, combined with transcriptomics data on the expression of channels in single cells or cell types, will provide a starting point for investigation of this vast, as yet unexplored, combinatorial space.

## Ethics Statement

All procedures were conducted in conformity with the Swiss Welfare Act and the Swiss National Institutional Guidelines on Animal Experimentation for the ethical use of animals.

## Author Contributions

RR, EL, and HM: conceptualization, writing, and supervision. RR, EL, and MM: methodology and data curation. RR: data analysis. RR and ES: coding and channelpedia. RR, EL, MM, MH, VT and VB: investigation.

### Conflict of Interest Statement

The authors declare that the research was conducted in the absence of any commercial or financial relationships that could be construed as a potential conflict of interest.

## Abbreviations

AP: Action potential
APC: Automated patch clamp
CHO: Chinese hamster ovary
CV1: *Cercopithecus aethiops* kidney
FT: Flp-In^™^T-Rex^™^
HEK: Human embryonic kidney
HSP: Heat shock proteins
IC: ion channel
Kv: Voltage-gated potassium channel
RT: Room temperature.
