# Supplementary material for: A Kinetic Map of the Homomeric Voltage-Gated Potassium Channel (Kv) Family
Source: Front Cell Neurosci. 2019 Aug 20;13:358. doi: 10.3389/fncel.2019.00358 (PMC6710402; doi:10.3389/fncel.2019.00358)
Supplement: Supplementary file 4 [file Data_Sheet_1.pdf]

EXPERIMENTAL PROCEDURES

RNA isolation and cDNA library synthesis

Total RNA was isolated from the whole brain, cerebellum or cerebral cortex of wild-type three months-old male Wistar-Han rats or (C57BL6/J) mice. All animal procedures were carried out in agreement with the animal authorization (VD1550) approved by the Veterinary Authorities and the Cantonal Commission for Animal experimentation of the Canton of Vaud according to the Swiss animal protection law. The brain tissue was collected in Qiazol® Lysis $_{\text{Reagent}}$ (Qiagen), grinded with plastic piston, sonicated for 2-3 pulses, followed by chloroform extraction and RNA purification through the RNeasy® Mini Kit (Qiagen), following the supplier's protocol. 5 µg of total RNA were primed by an oligo-dT primer and reverse transcribed using SuperScript® III Reverse Transcriptase (Invitrogen™-Life Technologies) according to the manufacturer's instructions (final volume of 20 µl). For Kv11.2 and Kv8.2 that were expressed at a very low level, we primed the RNA with a gene-specific reverse primer (GSP) instead of oligo-dT. For human gene amplification, PCR ready first strand human cDNA from cerebellum, cortex and thalamus were purchased from BioChain Institute Inc. About 6 ng of cDNA was used in each PCR reaction (see below). The quality and purity of total RNA was tested by spectrophotometric analysis.

IC gene amplification

Primers specific for each ion channel were designed using the DNASTAR Lasergene software SeqBuilder™ (see Table S6). 2 to 3 µl of cDNA were used for PCR amplification, using the high fidelity Pwo SuperYield DNA Polymerase (Roche Life Science) in the presence of GC-rich solution on an Eppendorf thermal cycler. A two-fragments cloning strategy was used to clone longer products (*KCNQ5, KCNH5, KCNH3* and *KCNH4)* (see GENEART® Seamless Cloning kit, Invitrogen™-Life Technologies). Details on PCR conditions used for the amplification of each ion channel are available on Channelpedia website in the form of datasheet on each Kv ion channel page. Despite many trials, we were not able to amplify rat *KCNG2, KCNA10, KCNQ4, KCNH8* and *KCNH7* from our cDNA libraries hence we chose to amplify them from purchased vectors (4 µg, pUC57, Purified DNA samples, GenScript®).

IC gene cloning

PCR products were cleaned-up using the NucleoSpin® Gel and PCR Clean-up kit (Macherey-Nagel GmbH) before direct cloning in the pENTR™ Directional TOPO® Cloning vector (TOPO/D-pENTR; Invitrogen™-Life Technologies), following the manufacturer's instructions. Transformants were analyzed after miniprep preparation (Machery-Nagel GmbH). Positive clones were selected according to size and verified by full DNA sequencing (Microsynth AG).

Expression plasmids were then generated using the Gateway® cloning system (Invitrogen™-Life Technologies). Briefly, the IC gene was transferred from the pENTR vector into a Gateway destination vector (pDEST) by LR recombination reaction using the Gateway® LR clonase® II Enzyme Mix (Invitrogen™-Life Technologies).

To produce a mammalian expression vector suitable for the Flp-In™T-Rex™ system, a Gateway version of the pcDNA5/FRT/TO vector was generated using the Gateway® Vector Conversion System (Invitrogen™-Life Technologies). In summary, the pcDNA5/FRT/TO vector (Invitrogen™-Life Technologies) was linearized by *pme1* restriction enzyme digestion before ligation of the RfA cassette. Transformants are analyzed after transformation in the *ccdB* Survival™ 2 T1® Competent *E. Coli*. Correct insertion of the cassette was analyzed by PCR using combination of specific sequencing primers of the pcDNA5/FRT/TO vector and RfA cassette. The generated gateway destination vector is referred to as pDEST, and a pDEST containing an ion channel gene as pDEST-IC vector (see also Figure S1).

After the LR Clonase reaction, correct recombination of the IC gene in the pDEST was confirmed by partial DNA sequencing. The NucleoBond®PC Plasmid purification kit (Macherey-Nagel GmbH), was used following the user instructions, to produce a high yield of DNA. High DNA quality suitable for transfection was determined by both agarose gel electrophoresis and UV spectroscopy (quotient A_{260}/A_{280}).

Cell lines handling and maintenance

The Flp-In™T-Rex™-HEK cell line was purchased from Invitrogen™-Life Technologies and cultured in DMEM medium supplemented with 10% fetal bovine serum (FBS) (Gibco™-Life

Technologies), 100 µg/ml Zeocin™ (Invitrogen™-Life Technologies) and 5 µg/ml Blasticidin S HCl (Gibco™-Life Technologies).

The Flp-In™-CHO and Flp-In™-CV1 cell lines were purchased from Invitrogen™-Life Technologies and transfected with pcDNA6/TR© (Invitrogen™-Life Technologies) to establish stable host cell lines constitutively expressing the tetracycline repressor (TetR). Cells were selected using 10 µg/ml (CHO) or 2.5 µg/ml (CV1) Blasticidin S HCl. The sensitivity of the cell lines to Blasticidin was preliminarily tested. Expression of TetR was checked by RT-PCR analysis and the cell batch expressing the highest level of TetR was selected and amplified. The generated Flp-In™T-Rex™-CHO cell line was maintained in RPMI Media 1640 supplemented with 5% FBS, 100 µg/ml Zeocin™ and 10 µg/ml Blasticidin S HCl. The generated Flp-In™T-Rex™-CV1 cell line was maintained in DMEM medium supplemented with 10% FBS, 100 µg/ml Zeocin™ and 2.5 µg/ml Blasticidin S HCl. All cells were grown at 37°C in a humidified 5% CO_2 atmosphere. For simplicity, Flp-In™T-Rex™ is abbreviated as FT.

Cell lines library construction and maintenance

To generate isogenic and inducible stable cell lines, 0.3 µg of each pDEST-IC vector were co-transfected with 1 µg of pOG44 (Invitrogen™-Life Technologies) into $5x10^5$ CHO-FT cells, $4x10^5$ CV1-FT cells or $1x10^6$ HEK-FT cells, plated on 6cm cell plate, using Lipofectamine2000 (Invitrogen™-Life Technologies), according to the manufacturer's instructions. Two days after transfection, Hygromycin B (500 µg/ml for CHO-FT, 150 µg/ml for CV1-FT and 300 µg/ml for HEK-FT) (Invitrogen™-Life Technologies) was added to select stable transfected cells. Cells were passaged twice a week in media containing Hygromycin B and Blasticidin S HCl, at the doses stated above. As Flp-In™T-Rex™ cell lines are isogenic (thanks to the Flp-In™ recombination site present in both vector and cell genome; see the Flp-In™T-Rex™ Core Kit Manual from Invitrogen™-Life Technologies), we performed polyclonal selection of Hygromycin-resistant cells. After sub-culturing each cell line for at least four passages, stable cell lines were verified for ion channel expression by RT-PCR analysis (see cell line validation). Stable cells were maintained in Hygromycin-containing medium at the concentrations used for selection. Cell stocks were made in a controlled-rate freezing apparatus following standard procedures. All cell lines were checked to

be free of mycoplasma. Stable cell lines are hereafter abbreviated as CHO-IC, CV1-IC and HEK-IC (with IC being the name of the ion channel stably transfected).

Induction of IC expression

Cells were plated and grown for 24 hours in their respective culture medium, free of antibiotics. The number of cells and the type of culture vessel depended on the specific experiment. Ion channel expression was induced by adding 1µg/µl Tetracycline (Doxycycline hyclate, Sigma-Aldrich®) in the culture medium for 24h.

Cell line validation by RT-PCR

Cells (0.5 million) were seeded on 6 cm plates and induced as stated above. RNA was isolated using the RNeasy® Mini Kit (Qiagen), following the supplier's protocol. First-strand cDNA was synthesized from 2 µg of total RNA using SuperScript® III Reverse Transcriptase and oligodT, according to the manufacturer's instructions, in a 20 µl final volume. Overexpression of the IC gene was then checked by PCR in comparison to non-induced cells. Briefly, 1 µl of a 1:50 dilution of the cDNA product was used for PCR amplification using gene-specific primers (Table S7) on a thermal cycler (SensiQuest) with 28 cycles of amplification. The housekeeping gene GAPDH was used as an internal control. Validation of each cell lines by RT-PCR is available on Channelpedia website (https://channelpedia.net or https://channelpedia.epfl.ch) in the form of datasheet on each Kv ion channel page.

Transcriptome analysis

Total RNA of CHO rKv1.1, CHO-FT, CV1-FT and HEK-FT cells were isolated using the RNeasy® Mini Kit, following the supplier's protocol. The quality and purity of RNA was tested by spectrophotometric analysis. 10 µg of each RNA sample was sent in dry ice to Beijing Genomics Institute (BGI), Hong Kong for HiSeq transcriptome sequencing. RNA-Seq technology was used for sequencing which resulted in on average 46,773,206 raw sequencing reads and then 44,469,969 clean reads per sample after filtering low quality. Clean reads were then mapped to reference genome (Human: GCA_000001405.22, GRCh38.p7; CHO: Jul. 2013 C_griseus_v1.0/criGri1) using HISAT/Bowtie2. Gene expression level was then quantified into FPKM (Fragments Per Kilobase of

transcript per Million mapped reads) values by a software package called RSEM. These FPKM values are reported for CHO, CV1 and HEK cell lines in Table S3.

Gene expression assay

A multiplexed detection of gene expression was performed for all CHO-IC cell lines (except for Kv6.2, Kv1.8 and Kv7.4 cell lines that were included later in the study) using the nCounter™ Gene Expression Assay according to the manufacturer's instructions (nCounter™ Gene Expression Assay User Manual, NanoString® Technologies*).* Cells were seeded on 6 cm plates and induced as stated above. After 24h, total RNA was isolated using the RNeasy® Mini Kit, following the supplier's protocol. 300ng of RNA were then hybridized with a custom designed CodeSet of 46 reporter probes (specific probes for the 37 IC genes and 9 CHO housekeeping genes) for 22 hours at 65°C. Each investigated nucleic acid was targeted by a capture probe and a reporter probe (50 nucleotides each, complementary to the region of interest). Each capture probe carries a biotin-tag for immobilisation with the nCounter PrepStation and each reporter probe was labeled with a barcode of six fluorophores that was unique for each targeted sequence. The fluorescent barcodes were imaged, decoded and counted with the nCounter Digital Analyser. All counts for all samples are listed in Table S4. Induced CHO-FT samples were used as control for background expression and the differential expression was calculated with

Differential count (gene, sampleID) = [ActualCount(gene, sampleID)-mean(CHO-FT(gene))]/std(CHO-FT(gene))

The differential count was calculated by first subtracting mean count from actual count and then dividing by standard deviation of CHO-FT. Differential count was plotted with Matlab for visualization (Figure S3).

Cell surface protein biotinylation

CHO-Kv1.1, Kv11.1, Kv7.5 and Kv5.1 cells were respectively seeded on eight 10 cm plates (1 million cells per plate) before induction. Ion channel expression was induced in four plates while the remaining plates were left untreated as control. Cell surface proteins were labelled with biotin and purified by using the Pierce™ Cell Surface Protein Isolation Kit (Thermo Fisher Scientific),

following the manufacturer's instructions. At the end of the cell lysis step, 5% of the total protein amount was collected as an "input" sample, to use as positive control in subsequent immunoblotting. Membrane fractions and input samples were tested for IC expression by western-blot. The expression of the intracellular protein Actin was tested as control for purity of the membrane fraction.

Immunoblotting

Cells were seeded on 6 cm plates (0.5 million cells per plate) prior to induction. In parallel, control cells were left untreated. Cells were then washed in PBS, collected by trypsinization and lysed in lysis buffer containing 50 mM Tris (pH 8), 150mM NaCl, 1% NP40 and protease inhibitor cocktail (cOmplete™, EDTA-free Protease Inhibitor Cocktail, Roche Life Science), at 4°C. Samples were sonicated (3 x 1s pulses) before incubation on ice for 30 minutes. Lysates were then clarified by centrifugation at 12'000 rcf for 5 minutes at 4°C. 3X Blue Loading Buffer-DTT (New England BioLabs®) was added to the clear supernatant and solubilized proteins were separated by SDS-PAGE following standard procedures. Proteins were transferred to nitrocellulose membranes (Bio-Rad) and blocked in TBS-T (TBS + 0.2% Tween 20) supplemented with 4% non-fat dried milk powder (AppliChem). Membranes were then incubated overnight at 4°C in TBS-T-milk with primary antibody at the following concentration: anti-Kv1.1 (1:500; K36-15, Neuromab), anti-Kv11.1 (1:500; APC-016, Alomone Labs), anti-Kv7.5 (1:500; APC-155, Alomone Labs), anti-Kv5.1 (1:500; LS-C384235, LifeSpan BioSciences, Inc) and anti-Actin (1:1000; ACTN05-C4, Abcam®). Finally, proteins were detected using the Pierce™ ECL Western Blotting Substrate (Thermo Fisher Scientific).

Cells preparation for automated patch clamp (APC) recordings

CHO-IC, CV1-IC or HEK-IC cells were seeded on a 10 cm plate ($1x10^6$, $0.5x10^6$ and $2x10^6$ cells per plate respectively) and grown for 24 h before induction. CHO and CV1 cells were washed twice in PBS, and gently detached with 1-minute incubation at 37°C with Trypsin 0.025% (Trypsin 0.05% EDTA Gibco™-Life Technologies diluted 1/1 in D-PBS). Trypsin was carefully aspirated and cells were re-suspended in HBSS (-$CaCl_2$, -$MgCl_2$) (Gibco™-Life Technologies) by gentle pipetting. HEK cells were detached by gently pipetting with suspension medium HBSS. Cells were suspended in

HBSS with concentration between 0.5 and 1 million cells/ml and suspension of 2 - 4 ml cells with no clusters or bubbles was loaded onto the cell hotel of a Nanion patchliner APC and constantly mixed at 300 - 700 µl at 15 s interval depending on the volume of suspension.

Electrophysiology environment

All electrophysiology experiments were performed in a dedicated 2.5m x 2.5m temperature-controlled room. An air conditioning equipment (NEXT DW OS, rcgroup.it) was used to set room temperature at 15, 25 or 35°C. Before the start of each experiment, the recording solutions were left long enough in the experiment room to ensure they were also at the same temperature.

All cells were recorded in whole-cell patch clamp configuration on Nanion's NPC-16 Patchliner Quattro (Nanion Technologies GmbH) fitted with EPC-10 HEKA Quadro amplifiers (HEKA Elektronik GmbH). The patchliner system also includes a temperature-control feature, which was used to set the same temperature as of experiment room. Disposable borosilicate (Nanion NPC-16) medium resistance (2-3 MΩ) glass chips were used for all recordings. The patchliner device records up to four cells simultaneously. The PatchControlHT software (Nanion Technologies GmbH) was used for the automation of patch clamp steps (cell capture, seal formation, whole-cell access, washing, etc.). The PatchControlHT software internally uses Patchmaster software (HEKA) for the data acquisition. The data were filtered with internal filter 1 (Bessel) at 10 kHz and filter 2 (I_Bessel) at 2.9 kHz of the EPC-10 and digitized at 10-50 kHz configuration according to different voltage protocols.

The extracellular solution (ECS) contained: 140 mM NaCl, 4 mM KCl, 1 mM $MgCl_2$, 2 mM $CaCl_2$, 5 mM D-Glucose monohydrate, 10 mM Hepes; pH 7.4 with NaOH, osmolarity 298 mOsm. The intracellular solution (ICS) contained: 50 mM KCl, 10 mM NaCl, 60 mM K-Fluoride, 20 mM EGTA, 10 mM Hepes; pH 7.2 with KOH, osmolarity 285 mOsm. The Seal enhancer solution (SES) contained: 80 mM NaCl, 3 mM KCl, 10 mM $MgCl_2$, 35 mM $CaCl_2$, 10 mM Hepes (Na+salt); pH 7.4 with HCl, osmolarity 298 mOsm. All solutions were filtered with 0.2 µm filter (GP Express PLUS membrane, Millipore) and stored in 50 ml aliquots at +4°C.

APC automation

Patchcontrol HT software (Nanion Technologies GmbH) was used for the automation of patch clamp experiment on Nanion Patchliner Robot. 4 ml vials were used for the extracellular, intracellular and seal enhancer solutions and placed at the location F(4,3), F(4,1) and F(4,2) on Patchliner recording station. The main steps involved in the automation and parameters are listed in Table S8 and implemented in a xml configuration file required by the Patchcontrol HT software.

Stimulation protocols

VRest: Resting potential of each cell was recorded in current clamp mode by applying 0 current for 5 s. The protocol was repeated 3 times with 6 s intervals after each repetition.

For all voltage-clamp protocols except Action Potential (AP), cells were held at holding potential of -80 mV and had identical beginning (first 100 ms) and end (last 100 ms). The first 100 ms were divided into three parts: 40 ms at holding potential (-80 mV), 10 ms at -90 mV and remaining 50 ms at holding potential (-80 mV). The last 100 ms were always held at holding potential (-80 mV).

Activation: To probe the steady state activation kinetics, a conventional step voltage protocol containing 18 sweeps starting from -90 mV to +80 mV in +10 mV step increments was applied; each sweep duration was 500 ms and inter-sweep interval was 5 seconds.

Deactivation: To probe the steady state deactivation kinetics, a two-pulse protocol with 12 sweeps was designed. The first pulse of 300 ms duration at +70 mV was applied to induce opening of channel and the 2nd pulse of 200 ms duration, with voltage steps from -80 mV to +30 mV in +10 mV increments was applied to measure the deactivation kinetics that occurs as the voltage is changed from one level to another.

Inactivation: To study the steady state voltage dependence of inactivation, a two-pulse protocol with 12 sweeps was designed. The first conditioning pulse of 1.5 s duration, with voltage steps from -40 mV to +70 mV in +10 mV step increments was applied to observe maximum inactivation. The following 2nd brief test pulse of 100 ms duration at a fixed voltage of +30 mV was applied to measure the inactivation due to the conditioning pulse.

Recovery: To study the dynamics of recovery from inactivation, a two-pulse protocol with 16 sweeps was designed. The first pulse of duration 1.5 s at +50 mV was applied to induce inactivation and then a test pulse of duration 200 ms at +50 mV was applied after holding cell at recovery potential -80 mV for variable intervals, from 50 ms to 2.3 s in steps of 150 ms.

Ramp: To assess the response of Kv channels to sub-threshold and supra-threshold voltage fluctuations, a series of four triangular voltage pulses between -80 mV and +70 mV, with varying duration (400 ms, 200 ms, 100 ms, 50 ms) and with 400 ms interval between each pulse was applied. A total of 5 sweeps with inter-sweep interval of 5 s was applied for this protocol.

AP: The 1.8 s long regular spiking action potential voltage protocol was applied to mimic the physiological stimuli. The train of action potentials at 18 Hz frequency were obtained from somatic whole-cell current clamp recordings from L2/3 pyramidal neuron at 34 ± 1°C in rat somatosensory cortex slices from animals at postnatal day 14.

Data management

A custom library written in C/C++ was used to read PatchMaster (www.heka.com) data files. Each recorded cell was assigned a unique identifier (Cell ID) and its raw data were stored in a file called rCell<ID>.nwb in neurodata without borders (NWB) file format. The NWB file contains all metadata (as ion channel, host cell, species, temperature, etc.) and the recorded data for each repetition of each protocol. Data were then analyzed off-line using a commercial software package (Matlab 8.6, The MathWorks Inc.). A matlab structures called aCell was created after analyzing raw data from each protocol and the extracted features were stored in a file called aCell<ID>.mat. Raw and analyzed files (rCell<ID>.nwb and aCell<ID>.mat) for each recorded cell are available for download from Channelpedia website (https://channelpedia.net or https://channelpedia.epfl.ch).

Feature extraction

Multiple kinetic features (see Figure 2) were extracted from each protocol, for each repetition and each cell. Figure 2 and Figure 5 include features extracted from 2nd repetition of activation protocol and 1st repetition of remaining protocols (Deactivation, Ramp, Inactivation, Inactivation recovery

and AP). Matlab program was used for all analysis and "fminsearch" function was used for parameter search and curve fitting.

The Activation protocol contained 18 sweeps of command voltages ranging from -90 mV to +80 mV in 10 mV increments. The recorded currents were normalized to maximum current (I_{max}). Each sweep of the recorded current was analyzed between 100.3 ms and 599.7 ms. Maximum value for this duration was searched for each sweep and marked as "I_{peak}".

I-V curve was obtained by fitting normalized peak_value (I_{peak}) against command voltage (from -90 mV to +80 mV) to a Boltzmann curve (Eq. 1). Act_Volt was searched along the I-V curve and the voltage where the current response exceeded 10% of the maximum current was marked as Act_Volt. Act_τ was obtained by fitting single exponential function (Eq. 2) to each current trace from 100.3 ms to I_{peak}.

$$IV = \frac{1.0}{1 + e^{\frac{v-vh}{-k}}} \tag{1}$$

$$I = 1 - e^{\frac{-t}{Act_\tau}} \tag{2}$$

Deactivation time constant $Deact_\tau$ was obtained by fitting (Eq. 3) to current traces between 400.5 ms and 599.4 ms recorded in response to deactivation protocol.

$$I = C + \left(A * e^{\frac{-t}{Deact_\tau}}\right) \tag{3}$$

Inactivation kinetics was measured with steady state inactivation, inactivation time constant and inactivation factor. For these features, the pre-pulse phase from 100.7 ms to 1599.2 ms, and test pulse from 1600.6 ms to 1699.2 ms from inactivation protocol were used. The steady state inactivation (IV) was captured by fitting a modified Boltzmann function (Eq. 4) to I_{peak} from the test pulse against the command voltage from pre-pulse phase. The inactivation time constant was measured from pre-pulse phase by fitting a modified exponential decaying curve (Eq. 5) to each current traces from the peak_value(I_{peak}) to end of the pre-pulse phase.

$$IV = A1 + \frac{1.0 - A1}{1 + e^{\frac{v - vh}{k}}} \tag{4}$$

$$I = C + \left(A2 * e^{\frac{-t}{Inact_\tau}}\right) \tag{5}$$

The inactivation factor refers to the extent of inactivation and was measured by subtracting $I_{95\%}$ (peak value at 1524.4 ms) from I_{peak}. The time constant of recovery from inactivation (Rec_τ) was measured from recovery protocol. To account for the run down effect, each sweep was normalized to pre-pulse stimulus response. Single exponential curve (Eq. 6) was fitted to the peak values of current traces during test pulse phase.

$$I = 1 - e^{\frac{-t}{Rec_\tau}} \tag{6}$$

$V_{MaxCond}$, the voltage corresponding to maximum conductance (peak) was obtained by searching peak value during the rising phase of the first ramp.

AP-Inactivation was calculated from the AP protocol. The current trace was first normalized to the maximal value and then 1- amplitude of the last (27th) AP was calculated as AP-Inactivation factor.

Activity index (AI)

Second repetition of activation protocol was used for the calculation of AI for each recorded cell. AI is the combination of SNR (signal to noise ratio) and non-linearity factor (NLF).

Normalized SNR (SNR_N) is calculated with (Eq. 7) and (Eq. 8)

$$SNR = \left(\frac{max(I_{Vsignal})}{max(I_{Vbase})}\right)^2 \text{where} \tag{7}$$

$$I_{Vsigna} = \frac{max(I_{v=30mV}) + max(I_{v=40mV})}{2} \; and \; I_{Vbase} = (I_{v\leq -60mV})$$

$$SNR_N = \begin{cases} 1, & if\ SNR > SNR_{Max} \\ \dfrac{SNR}{SNR_{Max}}, & if\ SNR \leq SNR_{Max} \end{cases} where\ SNR_{Max} = 300 \tag{8}$$

NLF is the measure of non-linearity of voltage response from -90 mV to 0 mV. All current responses were normalized by dividing maximal response between -90 mV and 0 mV. Since Kv channels do not open below -60 mV, the voltage range between -90 mV to -60 mV was considered for the calculation of leak current (I_0). I_0 was calculated by fitting a straight line between -90 mV and -60 mV and then extrapolating to 0 mV. Finally, NLF and AI were calculated with (Eq. 9) and (Eq. 10).

$$NLF = 1.0 - I_0 \tag{9}$$

$$AI = \frac{SNR_N + NLF}{2} \tag{10}$$

QA-QC for electrophysiology data

Electrophysiology data were filtered in multiple steps. V-offset, Seal, R-series and C-slow parameters were the first criteria applied to all cells. The acceptable range used as preliminary selection criteria were: V-offset < 45 mV, Seal (after whole cell configuration) >200 MΩ, R-Series < 15.5 MΩ, C-slow < 35 pF. A 2nd stage of filtering was applied based on ion channel category. For highly active ion channels (Kv1.x, Kv2.x, Kv3.x, Kv4.x, KV10.x), the cells with AI values > 0.3 and maximum current (from activation protocol) > 0.5 nA were considered as acceptable recordings. For low active ion channels (Kv7.x, Kv11.x, Kv12.x), AI values > 0.3 and no restriction on maximum current were applied. For silent channels (Kv5.1, Kv6.x, Kv8.x, Kv9.x), AI value < 0.3 was applied for the selection of valid cell. These criteria were sufficient to discard almost all bad recordings. However, still some cells (5.2 %) met all these criteria but had unstable membrane current. Those cells were manually excluded from the analysis. Data from all cells (good, bad, and excluded) are available on Channelpedia (https://channelpedia.net or https://channelpedia.epfl.ch) under each Kv channel page.

H-H model fitting

The fitting process starts with fitting a temperature independent H-H model to each individual cell. The current traces from voltage range between -50 mV and +50 mV of 2nd repetition of the Activation protocol were used for the model fitting. Current recorded at -50 mV was considered as

leak current and subtracted from rest of the current traces. The net transmembrane current $I_{Kv1.1}$ and the conductance $g_{Kv1.1}$ were computed using Eq. 11 and Eq. 12, with single gates for activation and inactivation.

$$I_{Kv1.1} = g_{Kv1.1}(V_m - E_k) \tag{11}$$

$$g_{Kv1.1} = \frac{I_{Kv1.1}}{(V_m - E_k)} = \bar{g}mh \begin{cases} E_k = -90.0, for\ 15°C \\ E_k = -93.1, for\ 25°C \\ E_k = -96.2, for\ 35°C \end{cases} \tag{12}$$

$$\frac{dm}{dt} = \frac{m_\infty - m}{m_\tau} \tag{13}$$

$$\frac{dh}{dt} = \frac{h_\infty - h}{h_\tau} \tag{14}$$

Gating variables (m, h) were modeled as a first-order kinetic process (Eq. 13, Eq. 14). $m_\infty\ and\ h_\infty$ were fitted with equations described in Eq. 15-16

$$m_\infty = \frac{1.0}{1 + e^{\frac{v-vh}{-k}}} \tag{15}$$

$$h_\infty = (1 - A) + \frac{A}{1 + e^{\frac{v-vh}{k}}} \tag{16}$$

m_τ was fitted to a combination of two Boltzmann curves defined in (Eq. 17 - 19) whereas h_τ was fitted with a single Boltzmann equation (Eq. 20).

$$m_{\tau 1} = A1 + \frac{B - A1}{1 + e^{\frac{v-c}{-d}}} \tag{17}$$

$$m_{\tau 2} = A2 + \frac{B - A2}{1 + e^{\frac{v-c}{d}}} \tag{18}$$

$$m_\tau = m_{\tau 1} + m_{\tau 2} \tag{19}$$

$$h_\tau = A + \frac{B}{1 + e^{\frac{v-c}{d}}} \quad (20)$$

To combine two Boltzmann curves as a smooth curve, a function mTauFunc (Figure 8D) was used. Experimental traces (-40 mV to +50 mV) from each cell (N=53, 71, 56) were fitted simultaneously with Matlab's unbounded optimization function "fminsearch" and resulted in one model for each cell.

All fitted models were evaluated against experimental data with residual sum of squares function RSS (Eq. 21).

$$RSS = \sum_{v=-40mV}^{50mV} (g_{\exp(v)} - g_{model(v)})^2 \quad (21)$$

After fitting all cells, RSS cut-off of 0.36 was used to select valid models. This selection resulted in (N=25, 65, 34) models for temperature 15, 25, and 35°C respectively (Figure 7B). Median values for each gating parameter were then recorded and used to formulate temperature-dependent H-H model.

Voltage-dependent gating parameters (median values) from experimental data at 15°C resulted in: m_∞= 1 / (1 + exp((volt - -16.965660) / -10.779574)); h_∞= 0.897737 + (0.101965 / (1 + exp((volt - -9.328883) / 7.491312))); m_τ= mTauFunc(v, -19.780, -13.203, 12.252, -58.529, 5.747, 5.090, 67.971, -22.772, 16.984); h_τ= 162.518286 + (333.208924 / (1 + exp((volt - -11.324982) / 9.092056))).

Voltage-dependent gating parameters (median values) from experimental data at 25°C resulted in: m_∞= 1 / (1 + exp((volt - -17.197057) / -9.206539)); h_∞= 0.506416 + (0.495818 / (1 + exp((volt - -20.135875) / 6.755450))); m_τ= mTauFunc(v, -30.138, -3.663, 25.025, -55.562, 7.531, 1.366, 26.772, -36.519, 15.380); h_τ= 119.600688 + (292.253701 / (1 + exp((volt - -14.895188) / 5.569321))).

Voltage-dependent gating parameters (median values) from experimental data at 35°C resulted in: m_∞= 1 / (1 + exp((volt - -8.386847) / -9.503428)); h_∞: 0.251120 + (0.785618 / (1 + exp((volt - -

36.585932) / 13.927393))); m_τ = mTauFunc(v, -57.076, -401.547, 35050.749, 14595.688, 3286.855, 0.640, 11.107, -40.568, 13.957); h_τ= 44.316090 + (270.171206 / (1 + exp((volt - -16.341487) / 6.397418))).

The final temperature model was fitted with temperature independent m_∞. For h_∞, a temperature-dependent linear Q_{10} function (Eq. 22) for steady state value of h_∞ was used. The linear Q_{10} function was obtained by fitting a straight line to median h_∞ values (at v = -50 mV) from three different temperatures as illustrated in Figure 7C.

$$h_\infty Q_{10} = (0.032 * °C) - 0.365 \tag{22}$$

For h_τ, a constant value of 2.7 was used to account for the temperature dependence. For $m_\tau Q_{10}$, Eq. 23 was used to approximate Q_{10} values observed from experimental data.

$$m_\tau Q_{10} = A * e^{\frac{-V}{B}} * e^{\frac{-°C}{C}} \tag{23}$$

The median gating parameters from all three temperatures along with Q_{10} functions (for h_∞, h_τ and m_τ) resulted in final temperature-dependent gating parameters illustrated in Figure 8D.

Figure generation

Phylogenic tree was constructed using Matlab "seqpdist" alignment function with parameter "ScoringMatrix" = "GONNET" and "seqlinkage" function with method parameter as "average". Radial cladogram in Figure 1A was created with Dendroscope (version 3.5.9) and later modified with Adobe Illustrator for visual presentation. Figure 1B, phylogenic tree was rearranged with Matlab function "phytreeviewer" to list ion channels in order from Kv1 to Kv12. The schematic and scaled depiction of the protein structure for each Kv channel was drawn in Adobe Illustrator.
