# Supplementary material for: A Kinetic Map of the Homomeric Voltage-Gated Potassium Channel (Kv) Family
Source: Front Cell Neurosci. 2019 Aug 20;13:358. doi: 10.3389/fncel.2019.00358 (PMC6710402; doi:10.3389/fncel.2019.00358)
Supplement: Supplementary file 5 [file Data_Sheet_2.pdf]

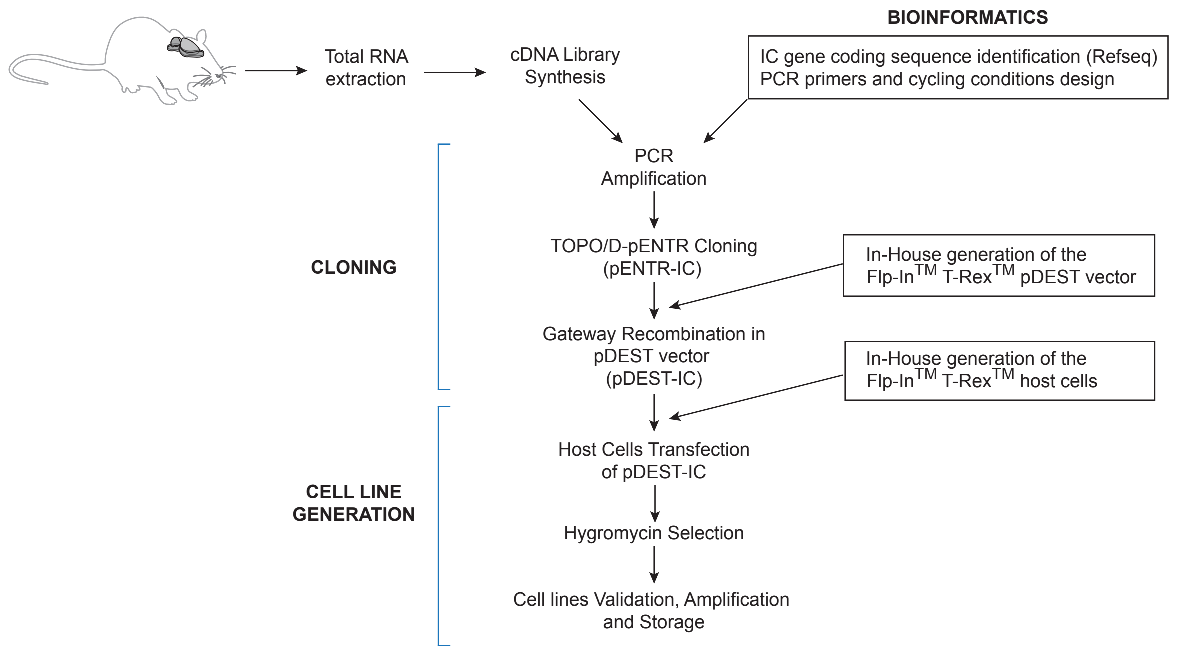

Figure S1 | The standardized cell line generation workflow, related to Figure 1C

A cDNA library is generated by reverse transcription of total RNA extracted from different rat brain regions (cerebellum, cortex or mixed regions) (see Method "RNA isolation and cDNA library synthesis"). In parallel, each Kv gene coding sequence (isoform 1) is retrieved from databases (GenBank, Ensembl). Specific primers (**Table S6**) and cycling conditions are designed for the amplification of each individual Kv gene.

Cloning: each Kv gene is amplified by PCR and cloned via a 2-steps cloning procedure based on the gateway® strategy (see Methods "IC gene amplification" and "IC gene cloning"), in a mammalian-expression vector suitable for the Flp-In™T-Rex™ expression system (in-house generated pDEST as described in Method "IC gene cloning"). The collection of pDEST-IC (expression vector containing an IC gene) is properly stored and catalogued.

Cell line generation: a stable and inducible cell line is generated for each Kv gene by transfection of the pDEST-IC in a Flp-In™T-Rex™ (FT) host cell line (CHO-FT, CV1-FT or HEK-FT: CHO-FT and CV1-FT were in-house generated as described in Method "Cell lines handling and maintenance"), followed by 2-3 weeks Hygromycin selection. Each cell line is validated (**Figure S2**) before being amplified, stored and catalogued.

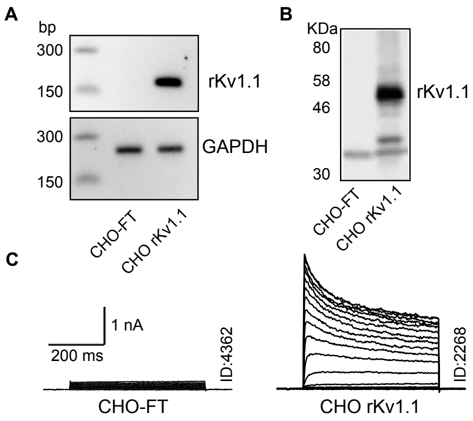

Figure S2 | The CHO rKv1.1 cell line validation, related to Figure 1

CHO-FT (as control) and CHO rKv1.1 cell lines are treated 24 h with tetracycline (see Method "Induction of IC expression") to induce Kv gene expression, and analyzed for gene expression **(A)**, protein expression **(B)** and electrical response **(C)**.

(A) rKv1.1 mRNA expression tested by RT-PCR; GAPDH mRNA expression tested as control (see Method "Cell line validation by RT-PCR"). **(B)** rKv1.1 protein overexpression tested by western-blot. **(C)** Current response to whole-cell voltage-clamp activation protocol performed at 25°C. Each cell line is tested for specific gene expression and electrical response before kinetic characterization (see datasheet on Channelpedia); western-blot is additionally performed upon availability of antibody.

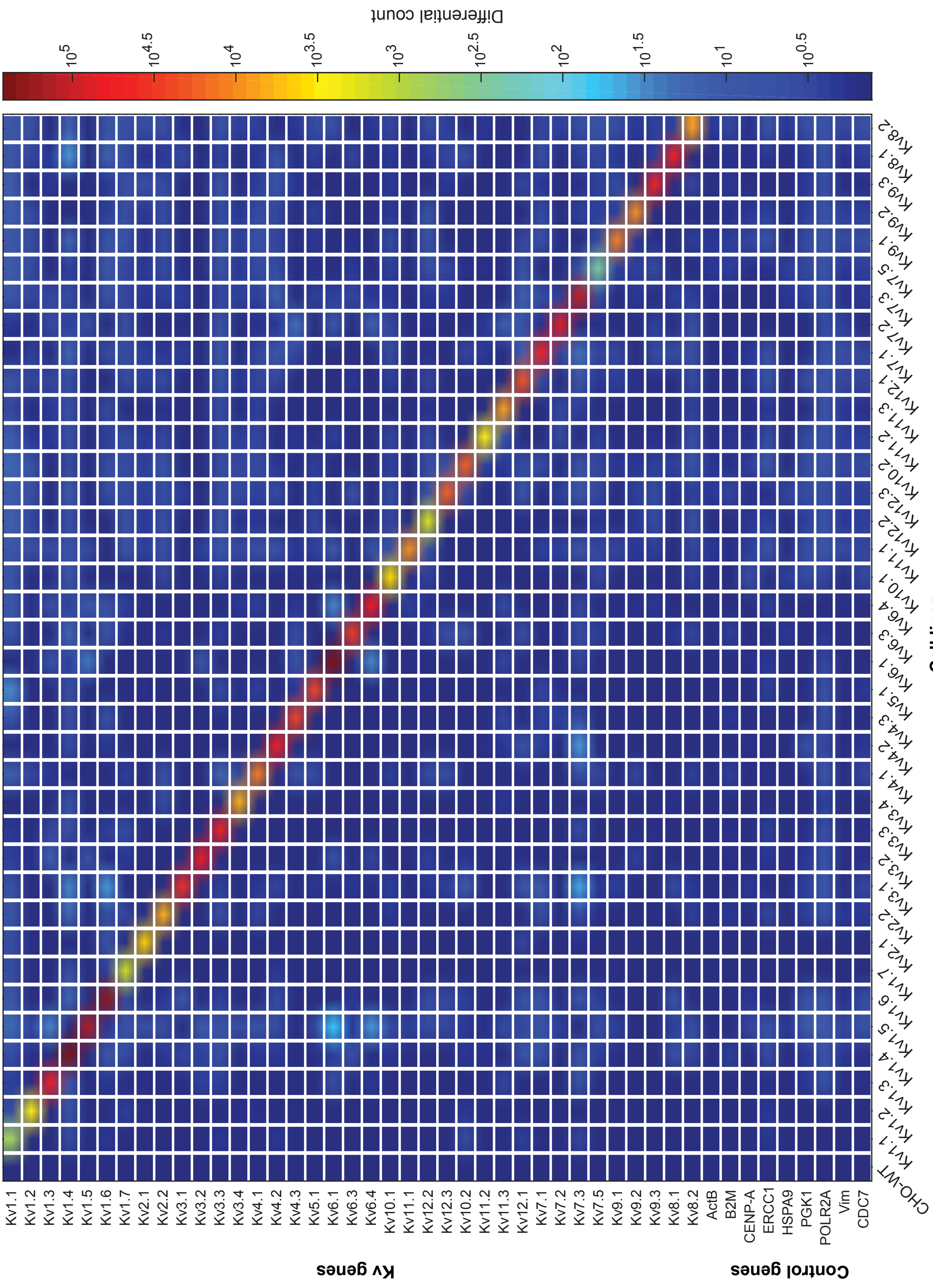

Figure S3 | Kv gene expression screening, related to Figure 1
A multiplexed detection of mRNA expression is performed for all CHO-Kv cell lines (except for kv1.8, kv6.2 and kv7.4 as these cell lines were included later in the study) using the nCounter gene expression assay (see Method "Gene expression assay"). Differential expression counts are plotted using a color-coded heat map. Absolute counts values are available in **Table S4**.

A

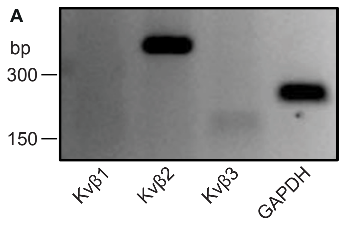

B

	Fw	Rv
Kvβ1	tcagatgaggttgctgaacg	tccaccccagtagagtttgg
Kvβ2	tgacaatggcatcaacctgt	gtaggcctccatgatctcca
Kvβ3	ggtacctgggtcacatttgg	gatctcctccaacctttgct
GAPDH	ttgtgatgggtgtgaaccac	ggatgcagggatgatgttct

C

FPKM	CHO-FT	CHO rKv1.1
Kvβ1	0.25	0.25
Kvβ2	130	111
Kvβ3	0.49	0.58
Kv1.1	2.2	2113

Figure S4 | Kv-beta subunits expression, related to Figure 2
The CHO rKv1.1 cell line is tested for endogenous expression of the Kvβ subunits by RT-PCR **(A** and **B)** and transcriptome analysis **(C)**.
(A) mRNA expression of Kvβ1, Kvβ2 and Kvβ3 is tested in CHO rKv1.1 cell line by RT-PCR; GAPDH mRNA expression is tested as control. **(B)** Sequences of primers used in panel A. **(C)** Absolute count values (FPKM) of Kvβ1, Kvβ2, Kvβ3 and Kv1.1 mRNA in CHO-FT and CHO rKv1.1 cell line (see Method "Transcriptome analysis"). For analysis, alignment was done on CHO genome.

A

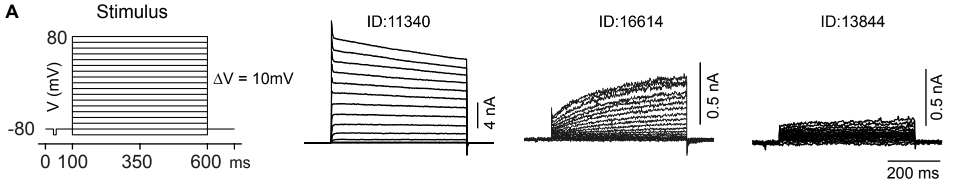

B

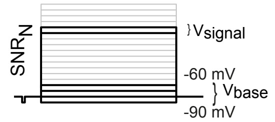

$$SNR = \left(\frac{max(I_{Vsignal})}{max(I_{Vbase})}\right)^2 \qquad SNR_N = \left(\frac{SNR}{SNR_{Max}}\right)$$

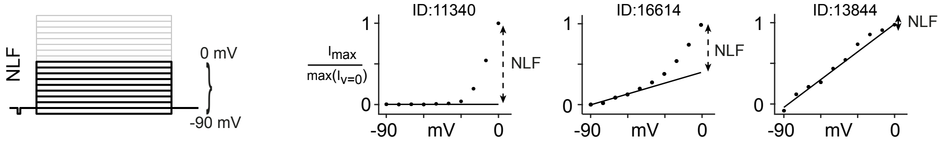

$$AI = \frac{SNR_N + NLF}{2}$$

ID:11340			ID:16614			ID:13844		
SNR_N	NLF	**AI**	SNR_N	NLF	**AI**	SNR_N	NLF	**AI**
0.99	1.0	**0.99**	0.61	0.58	**0.59**	0.0	0.03	**0.01**

C

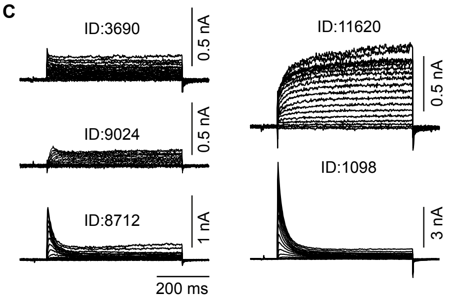

Cell ID	SNR_N	NLF	AI
3690	0.14	0.35	0.24
9024	0.22	0.68	0.45
8712	0.87	0.26	0.56
11620	0.26	0.51	0.38
1098	0.71	0.22	0.46

Figure S5 | Activity index, related to Figure 3
(A) Activity index (AI) is calculated from current response upon application of activation stimulus as shown. Current traces from three different cells (highly active ID:11340, low active ID:16614 and silent cell ID: 13844), are shown as exemplar for AI value calculation. Cell ID is indicated for each trace. **(B)** The normalized signal to noise ratio (SNR_N) and non-linearity factor (NLF) are two parameters used for the calculation of AI values. Voltage range (-90 to -60 mV) is used for base current (I_{Vbase}) calculation and voltage range (+30 to +40 mV) is used for signal ($I_{Vsignal}$) current. NLF is the measure of voltage-dependent non-linearity, calculated from current response of the voltage range (-90 to 0 mV) (see Method "Activity index"). Calculated SNR_N, NLF and AI values for each exemplar cell are shown in the table. The AI value for a highly active cell is close to 1 whereas it is close to 0 for an electrically silent cell. **(C)** AI values for other five cells with different levels of activity are shown as additional examples.

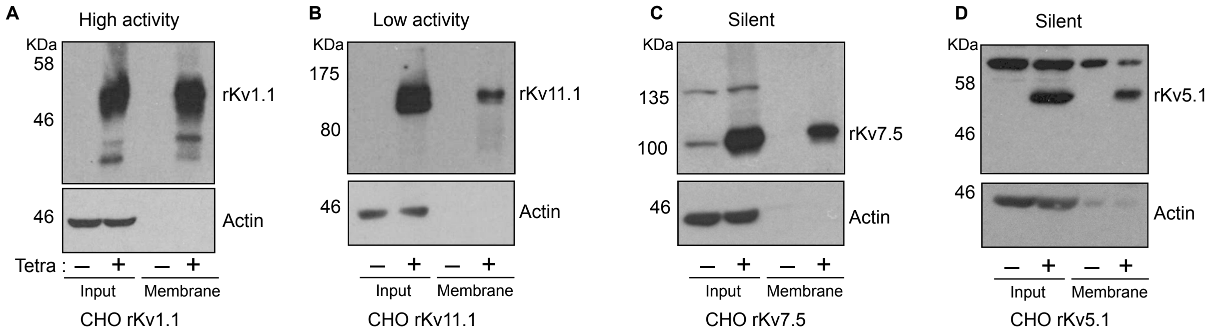

Figure S6 | Membrane localization of Kv channels, related to Figure 3
Two active CHO Kv cell lines (Kv1.1 **(A)** and Kv11.1 **(B)**) and two silent CHO Kv cell lines (Kv7.5 **(C)** and Kv5.1 **(D)**) are induced 24 h with tetracycline before biotinylation-based fractionation assay followed by western-blot (see Method “Cell surface protein biotinylation”). Non-induced cells are used as control. “Input” represents the total extract as control of protein expression. Actin is used as a control of membrane fraction purity.

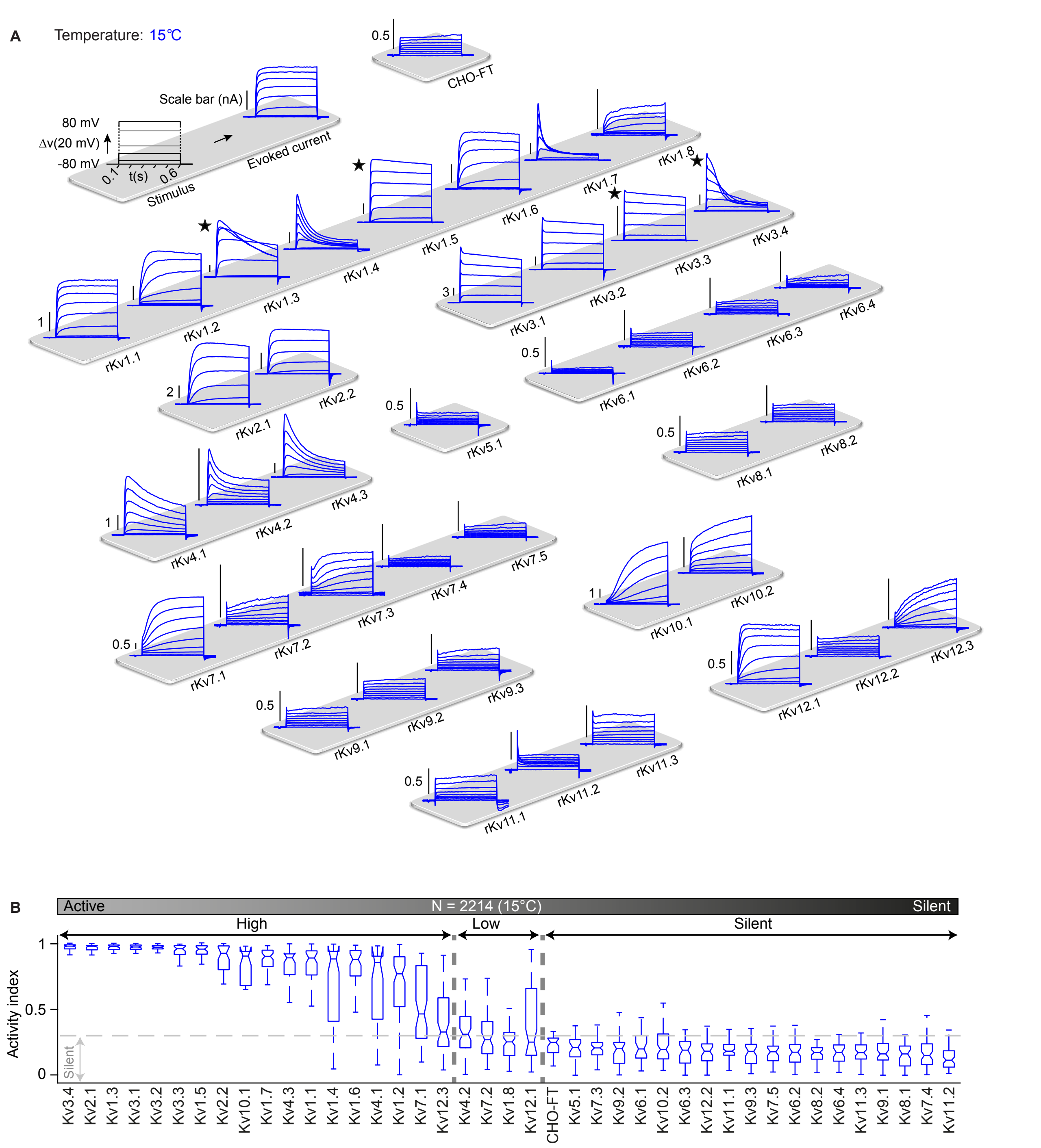

Figure S7 | Kv kinetics map at 15°C, related to Figure 3
(A) Illustration of activation stimulus with 20 mV steps and evoked current response are shown (top left panel). The amplitude is indicated in nA with scale bar. The non-transfected CHO-FT cell line is shown as control for the background current (top right panel). Representative traces of the typical response to activation stimulus for each Kv channel, recorded at 15°C is shown. Current traces are sorted by Kv subfamily. ★ indicates channels with inherent kinetic heterogeneity (see also **Figure 6**); for these channels only one response from the range of recorded responses is shown. **(B)** Box plots of AI values for each ion channel at 15°C. Ion channels are ordered by their median AI values and categorized as active or silent based on the 0.3 cut-off value for the 3rd quartile of the box plot (N = 2,214 cells). Active channels are further divided into highly active or low active based on 0.3 cut-off value for the median value of the box plot.

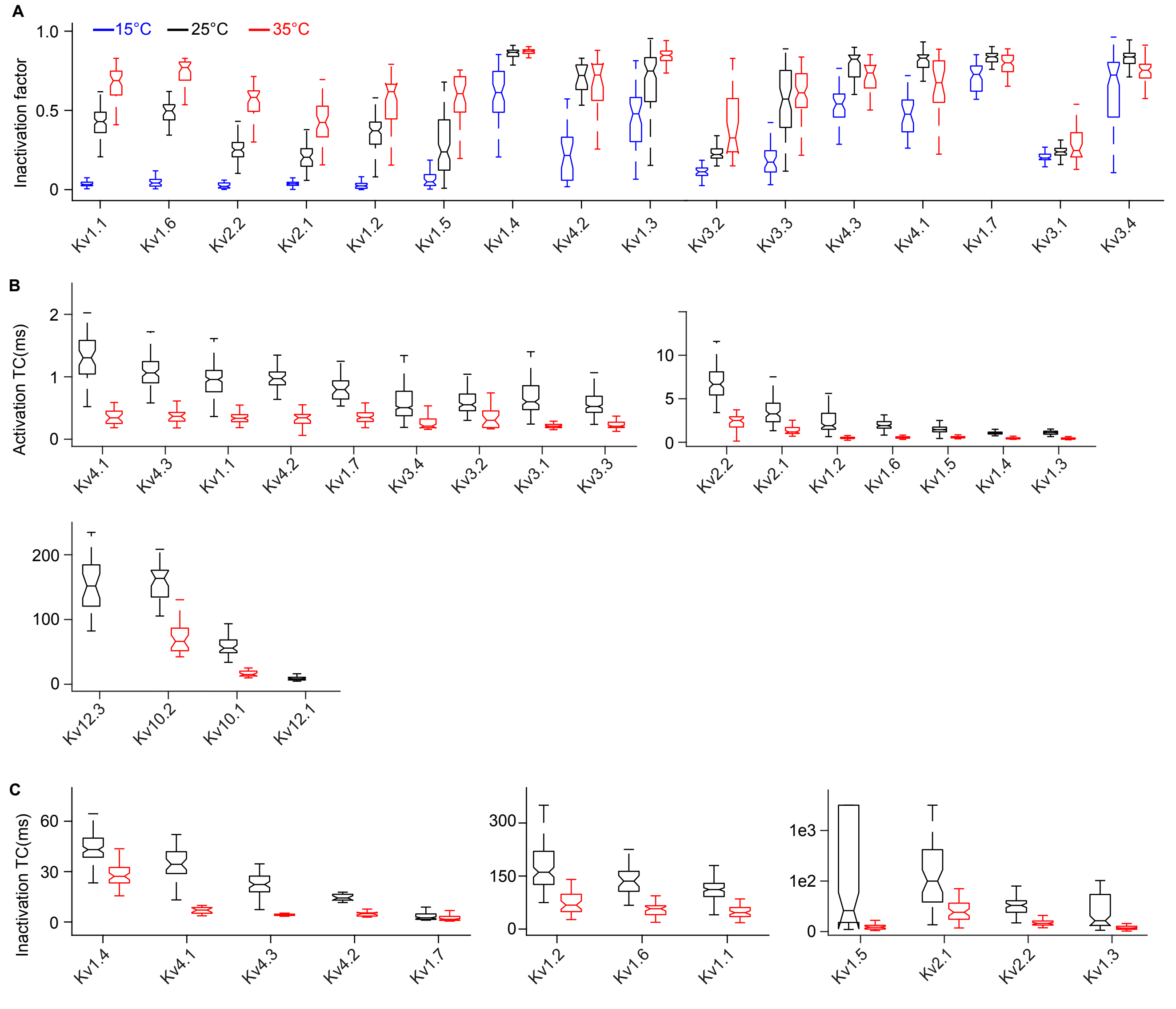

Figure S8 | Effect of temperature on Kv channels activation and inactivation, related to Figures 3, 4 and S7
(A) Box plots of inactivation factor for inactivating Kv channels at 15°C, 25°C and 35°C illustrate that many channels which show non-inactivating behavior (inactivation factor close to 0) at 15°C are actually inactivating at 35°C. **(B)** Box plots of activation time constant for all active Kv channels at 25°C and 35°C show that activation becomes faster at higher temperature. Kv11.1 and kv11.3 are not included. **(C)** Box plots of inactivation time constant for inactivating Kv channels at 25°C and 35°C show that inactivation becomes faster at higher temperature. Channels from Kv3 family are not included because of delayed inactivation.

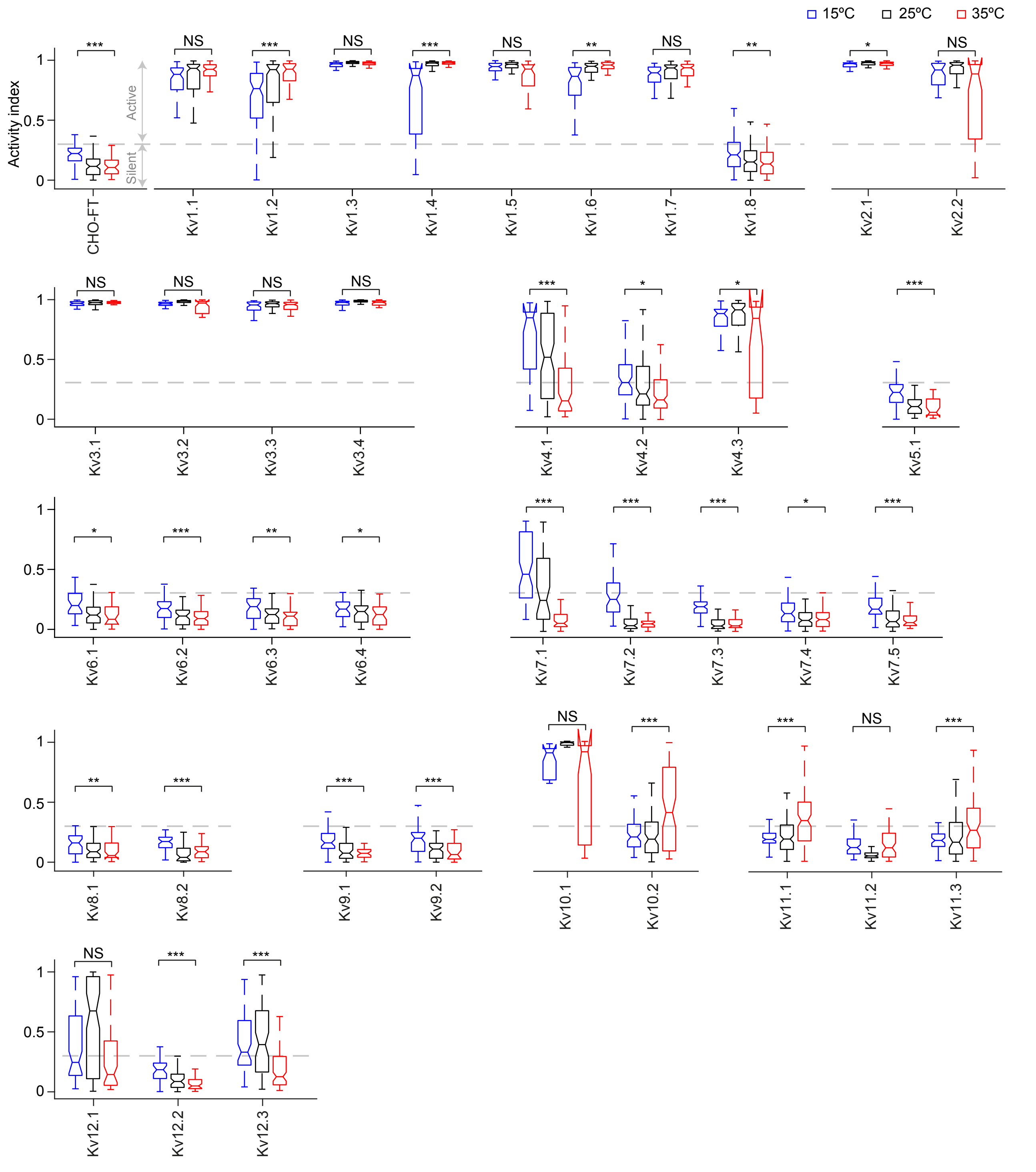

Figure S9 | Temperature effect on Kv channels activity, related to Figure 4
AI values at 15°C, 25°C and 35°C are plotted for all cells of all Kv channels and sorted by Kv subfamily (* p-value<0.05; ** p-value<0.01; *** p-value<0.001, Student's *t* test). The dashed line represents the AI value cut-off (0.3) that differentiates silent and active Kv channels.

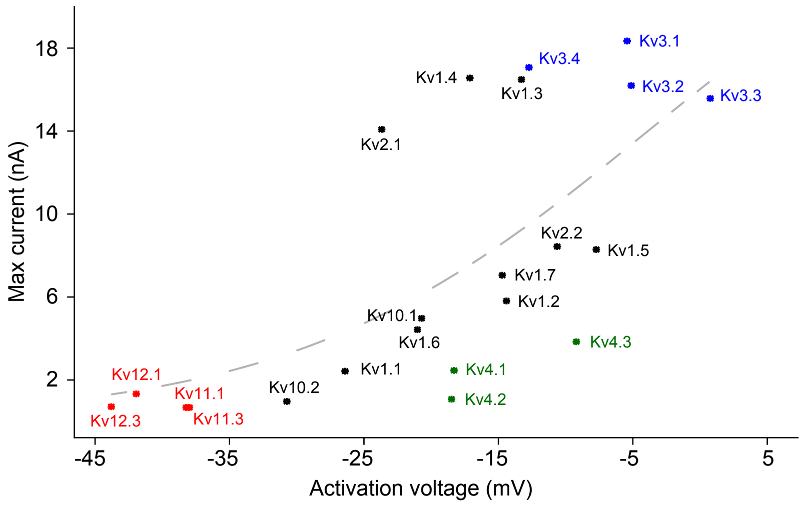

Figure S10 | Max current against activation voltage for active Kv channels, related to Figure 5
Observed maximum current (median values) in response to activation protocol at 35°C for all active Kv channels are plotted against their activation voltage (median values) indicating clusters of ion channel families activated at low voltage (in red) or high voltage (in blue).

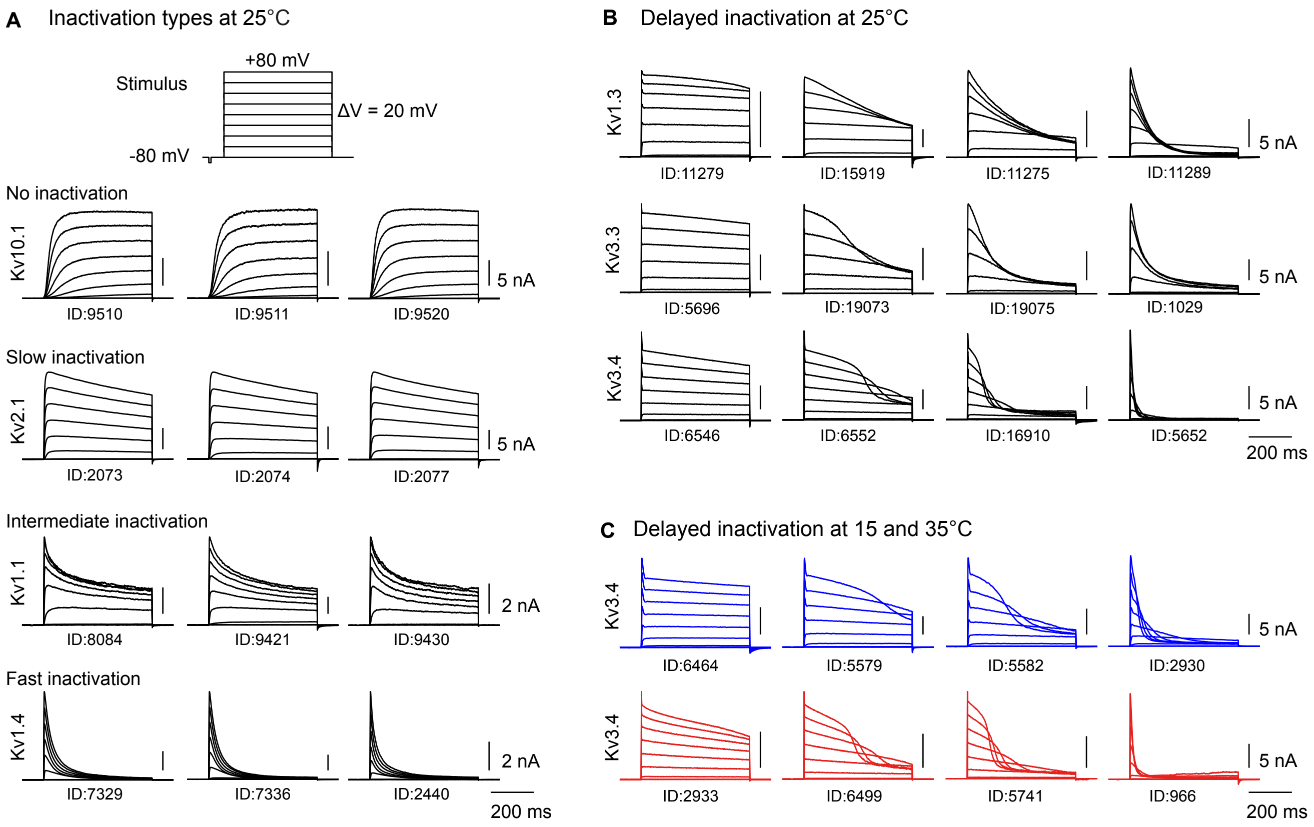

Figure S11 | Delayed inactivation, related to Figure 6
(A) The different patterns of inactivation (non-inactivation, slow inactivation, intermediate inactivation and fast inactivation) are represented by three exemplar traces for Kv10.1, Kv2.1, Kv1.1 and Kv1.4 cell lines in response to activation protocol (as depicted on top panel) at 25°C. **(B)** Representative traces of the range of kinetics, with delayed inactivation, for Kv1.3, Kv3.3 and Kv3.4, in response to activation protocol at 25°C are shown. **(C)** Representative traces of the range of kinetics, with delayed inactivation, for Kv3.4 in response to activation protocol at 15°C and 35°C are shown.
Cell ID is indicated below each trace.

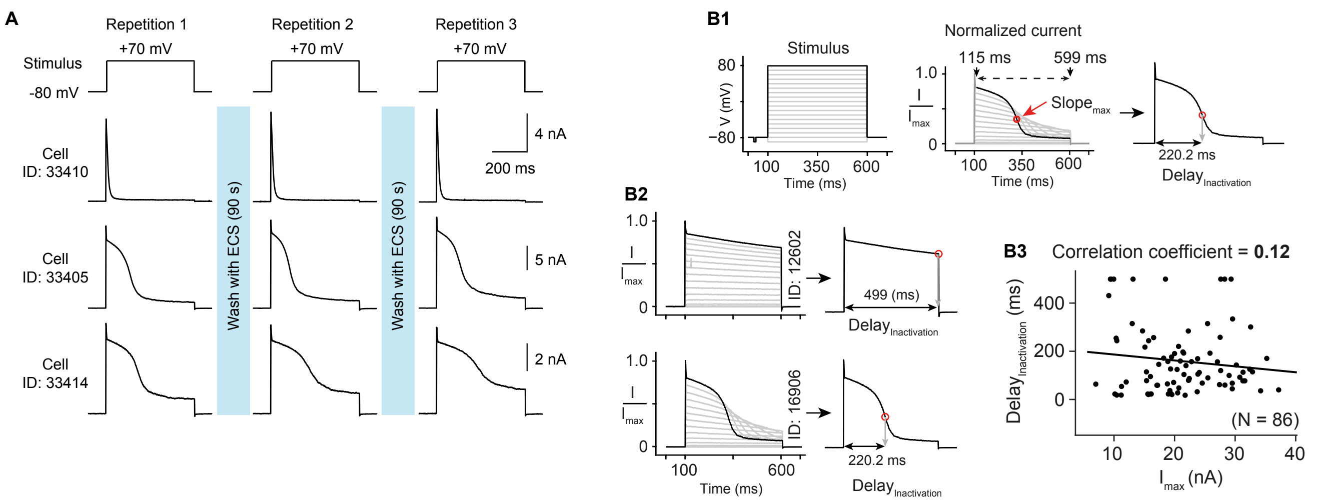

C

Transcriptome	FPKM Values CHO WT	FPKM Values CHO rKv3.4
Kv3.4	1.59	1298.66

House keeping genes	CHO WT	CHO rKv3.4
ActB	2656.72	2453.98
CENP-A	45.66	55.96
ERCC1	58.77	61.8
HSPA9	126.31	205.69
PGK1	438.61	382.23
Vim	1717.27	1588.9
cdc7	32.02	28.7

Ih channels	CHO WT	CHO rKv3.4
HCN1	0	0
HCN2	0	0
HCN3	0.18	1.57
HCN4	0	0

Ca channels	FPKM Values CHO WT	FPKM Values CHO rKv3.4
Cav1.1	0.02	0.07
Cav1.2	1.89	1.55
Cav1.3	0	0
Cav1.4	0.02	0.02
Cav2.1	0	0.02
Cav2.2	0.07	0.02
Cav2.3	0.02	0
Cav3.1	3.91	3.69
Cav3.2	0	0
Cav3.3	0.04	0

Na channels	FPKM Values CHO WT	FPKM Values CHO rKv3.4
Nav1.1	1.07	0.87
Nav1.2	0	0
Nav1.3	3.22	0.96
Nav1.4	0	0.02
Nav1.5	0	0.02
Nav1.6	2.96	2.2
Nav1.7	0.02	0
Nav1.8	0	0
Nav1.9	0.02	0
Nav2.1	0.02	0

Figure S12 | Kv3.4 delayed inactivation and kinetic heterogeneity, related to Figure 6
(A) Control experiment to wash out extracellular potassium accumulation. Single voltage pulse of +70 mV, followed by 90 s wash with extracellular solution (ECS) was applied multiple times. Evoked current response from exemplar Kv3.4 cells (ID 33410, 33405 and 33414) at 25 °C shows delayed inactivation even after multiple washes with ECS. **(B1)** Inactivation delay is calculated from normalized current response corresponding to +80 mV of the activation stimulus protocol. Inactivation delay ($Delay_{Inactivation}$) is the difference in time between start of stimulus (100 ms) to the point where maximum slope (>1.5e-4) is observed. Maximum slope is searched from 115 ms to 599 ms and in case where delayed inactivation is not present or maximum slope does not exceed 1.5e-4, 499 ms is considered as inactivation delay. **(B2)** In response to activation protocol, normalized current traces and calculated inactivation delay for two different exemplar Kv3.4 cells are shown. The current response corresponding to +80 mV is shown in black. Cell ID is indicated next to each trace. Please note that for Cell ID 12602, where delayed inactivation is not present and maximum slope does not exceed 1.5e-4, 499 ms is considered as inactivation delay. **(B3)** Correlation plot between inactivation delay and maximum current amplitude at 25°C for CHO cells expressing rat Kv3.4 ion channel. **(C)** Transcriptome data from Kv3.4 cell line do not show expression of native Ih, Ca or Na channels.

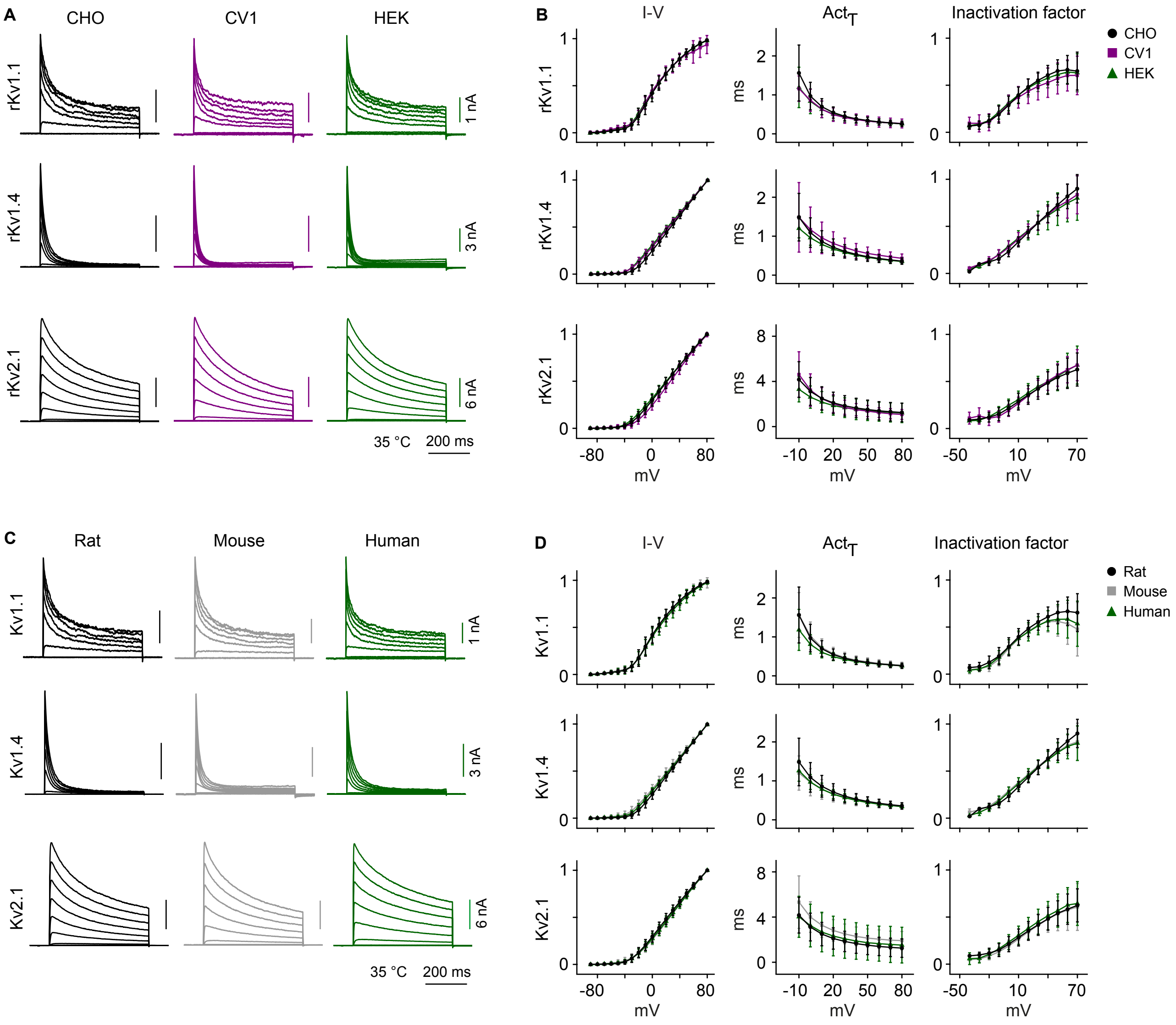

Figure S13 | Kv channel kinetics across host cell lines and species at 35°C, related to Figure 9
(A-B) Comparison of Kv kinetics across three mammalian host cell lines. **(A)** Representative current traces from rat Kv1.1, Kv1.4 and Kv2.1 expressed in the three different cell lines, in response to activation protocol at 35°C. **(B)** Median values for three kinetic features (I-V curve, activation time constant and inactivation factor calculated as explained in **Figure 2**) obtained from CHO, CV1 and HEK host cell lines for each Kv channel, overlaid for comparison.
(C-D) Comparison of Kv kinetics across three species. **(C)** Representative current traces for rat, mouse and human Kv1.1, Kv1.4 and Kv2.1 expressed in CHO cells, in response to activation protocol at 35°C. **(D)** Median values for three kinetic features (I-V curve, activation time constant and inactivation factor calculated as explained in **Figure 2**) obtained from rat, mouse and human genes of each Kv channel, overlaid for comparison.
Error bars are ± S.D. The amplitude in nA and time in ms are indicated with scale bars.

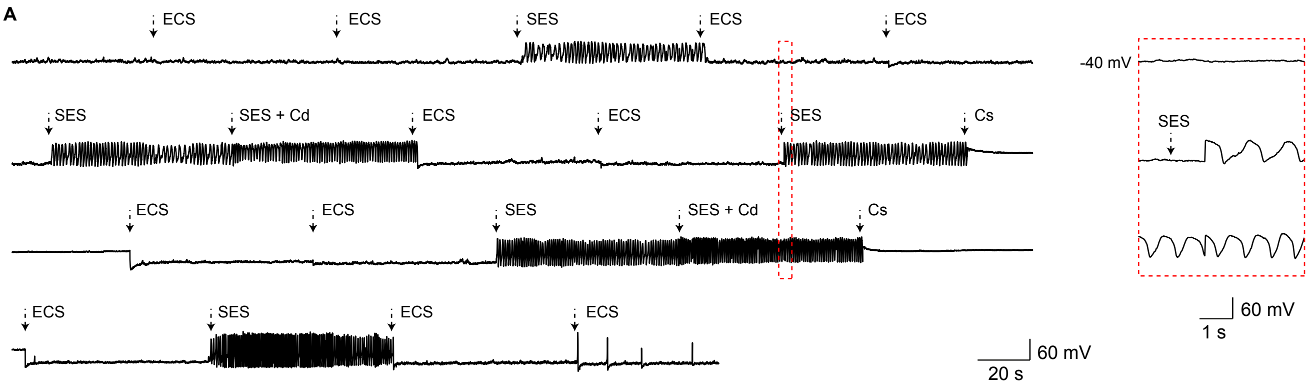

ECS : 140 mM NaCl, 4 mM KCl, **1 mM $MgCl_2$**, **2 mM $CaCl_2$**, 5 mM D-Glucose, 10 mM Hepes

SES : 80 mM NaCl, 3 mM KCl, **10 mM $MgCl_2$**, **35 mM $CaCl_2$**, 10 mM Hepes

SES + Cd : SE + 800 µM $CdCl_2$

Cs : 50 mM CsCl, 10 mM NaCl, 60 mM CsF, 20 mM EGTA, 10 mM Hepes

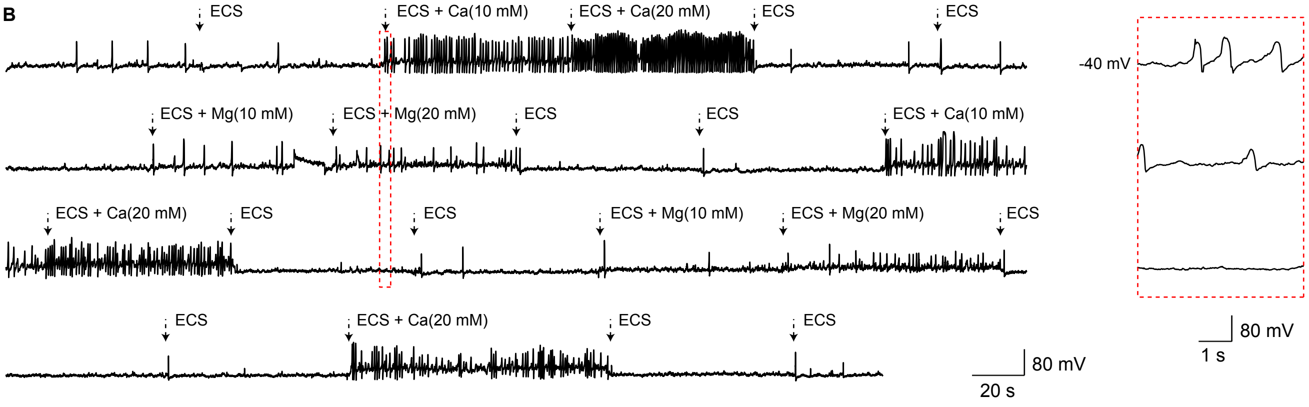

ECS : 140 mM NaCl, 4 mM KCl, **1 mM $MgCl_2$**, **2 mM $CaCl_2$**, 5 mM D-Glucose, 10 mM Hepes

Figure S14 | High concentrations of Ca^{2+} and Mg^{2+} in seal enhancer solution (SES) cause membrane potential oscillations for Kv11.1 cells
(A) Seal enhancer solution causes membrane potential oscillation for Kv11.1 expressing CHO cells. Continuous 24 minutes recording of membrane potential upon application of different bath solutions (illustrated with downward arrows) in whole cell current clamp configuration (Cell ID 14234). Expanded 5 s interval is shown in red box. Application of 800 µM $CdCl_2$ (Ca^{2+} channel blocker) with SES does not block oscillation. Wash with extracellular solution (ECS) or Cesium solution blocks membrane potential oscillation. **(B)** High concentrations of Ca^{2+} and Mg^{2+} in ECS cause membrane potential oscillations for Kv11.1 cells. Upon application of different concentrations of Ca^{2+} and Mg^{2+} in ECS (illustrated with downward arrow), continuous 25 minutes recording of membrane potential in whole cell current clamp configuration (cell ID 14277). Expanded 5 s interval is shown in red box.
