# Supplementary material for: A Kinetic Map of the Homomeric Voltage-Gated Potassium Channel (Kv) Family
Source: Front Cell Neurosci. 2019 Aug 20;13:358. doi: 10.3389/fncel.2019.00358 (PMC6710402; doi:10.3389/fncel.2019.00358)
Supplement: Supplementary file 6 [file Data_Sheet_3.pdf]

Table S1. Sequence description of the Kv channel genes used in the study, related to Figures 3, 4, 9, S7 and S12.

Nucleotides variations with respect to the original refSeq are listed.
Synonymous modifications introduced in the cloning primers to improve primer quality are labeled as “modified nt”. Unreported missense variations shown in bold, have been corrected by mutagenesis and tested for functionality. These variations do not affect the activity of the corresponding channels (data not shown).

[1] *KCND3*-isoform 2 is the only isoform we detected in the rat brain. This isoform has a 19aa shorter C-tail compared to isoform 1.

[2] The amplified sequence of *KCNQ3* corresponds to a former sequence version (NM_031597.3). The new version (NM_031597.4) contains 117 supplementary nucleotides at the 5’-end that we did not detect in the rat brain.

[3] This deletion was previously reported as XM_001071249, the only isoform we detected in the rat brain.

[4] We amplified two sequences for rat *KCNH1* and *KCNQ2*. As these Refseqs are provisional in NCBI, we chose sequences homologous to human and mouse isoform 1: NM_031742 with an 81nt insertion at position nt952 for *KCNH1*; NM_133322 with a 54nt insertion at position nt1248 and a few nucleotide differences for *KCNQ2*.

Gene	Protein	NCBI Ref Seq	Nucleotide modifications compared to RefSeq
**Rat Kv genes**			
KCNA1	Kv1.1	NM_173095	
KCNA2	Kv1.2	NM_012970	nt396>C (synonymous, reported in Ensembl)
KCNA3	Kv1.3	NM_019270	modified nt15>A; nt297>C (synonymous, rs199378897)
KCNA4	Kv1.4	NM_012971	Codons nt126>GCT and nt925>GGC (missense, reported in Ensembl)
KCNA5	Kv1.5	NM_012972	modified nt15>T
KCNA6	Kv1.6	NM_023954	
KCNA7	Kv1.7	NM_001108914	modified nt15>T
KCNA10	Kv1.8	NM_0001191713	
KCNB1	Kv2.1	NM_013186	
KCNB2	Kv2.2	NM_054000	nt2292>A (synonymous, not reported)
KCNC1	Kv3.1	NM_012856	
KCNC2	Kv3.2	NM_139216	
KCNC3	Kv3.3	NM_053997	out of frame �TAG� insertion nt1981 (reported in Ensembl: Kcnc3-203)
KCNC4	Kv3.4	NM_001122776	modified nt15>T; nt657>T (synonymous), nt1817>G (missense) (both reported in Ensembl), nt1492>G (missense, not reported)
KCND1	Kv4.1	NM_001105748	modified nt12>A
KCND2	Kv4.2	NM_031730	nt1173>T (synonymous, not reported)
KCND3	Kv4.3	NM_001270963	(= isoform 2)[1]
KCNF1	Kv5.1	NM_001169104	nt31>G (missense, rs198924451)
KCNG1	Kv6.1	NM_001106545	
KCNG2	Kv6.2	NM_001107372	
KCNG3	Kv6.3	NM_133426	
KCNG4	Kv6.4	NM_001107435	
KCNQ1	Kv7.1	NM_032073	modified nt12>T, nt1402>G (missense, not reported), nt1912>G (missense, reported in Ensembl)
KCNQ2	Kv7.2	Kcnq2_201[4]	Δ(1526-1528), nt 2447>G (missense, not reported), 7 others synonymous variations (reported in NM_133322).
KCNQ3	Kv7.3	NM_031597[2]	nt1656>T, nt2127>C, nt2505>T (all synonymous: rs105980670, rs198097269, rs105394238)
KCNQ4	Kv7.4	XM_008764109	
KCNQ5	Kv7.5	NM_001134643	Δ(1246-1302)[3], nt1770>A, nt1815>C, nt2232>C, nt2748>A (all synonymous, not reported)
KCNV1	Kv8.1	NM_021697	
KCNV2	Kv8.2	NM_001106370	nt306>A (synonymous, not reported)
KCNS1	Kv9.1	NM_053954	modified nt18>A
KCNS2	Kv9.2	NM_023966	
KCNS3	Kv9.3	NM_031778	modified nt12>C; nt327>T, nt900>T (synonymous, not reported)

KCNH1	Kv10.1	kcnh1_003[4]	
KCNH5	Kv10.2	NM_133610	
KCNH2	Kv11.1	NM_053949	nt156>T (synonymous, not reported), codon nt900>CCT (missense, reported in Ensembl)
KCNH6	Kv11.2	NM_053937	nt405>T, nt414>G, nt786>C (synonymous, rs107171774, rs106162519, rs105164542), nt2744>A (missense, rs105334634), nt1538>A (missense, not reported)
KCNH7	Kv11.3	NM_131912	
KCNH8	Kv12.1	NM_145095	
KCNH3	Kv12.2	NM_017108	
KCNH4	Kv12.3	NM_053630	modified nt15>A; nt1640>G (missense, not reported)
**Mouse Kv genes**			
KCNA1	Kv1.1	NM_010595	modified nt15>A
KCNA4	Kv1.4	NM_021275	modified nt6>G and nt12>A
KCNA5	Kv1.5	NM_145983	modified nt15>T and nt1794>T, and nt2557>A
KCNA6	Kv1.6	NM_013568	
KCNB1	Kv2.1	NM_008420	modified nt12>T and nt2557>A
**Human Kv genes**			
KCNA1	Kv1.1	NM_000217	
KCNA4	Kv1.4	NM_002233	modified nt9>G
KCNA5	Kv1.5	NM_002234	nt1149>C (synonymous, rs2359641)
KCNA6	Kv1.6	NM_002235	
KCNB1	Kv2.1	NM_004975	modified nt12>T

Table S2. New variants cloned from rat brain, related to Table S1

Brief description of seven new variants of the Kv genes cloned from rat brain.

Gene	Variant	Variation	Effect
KCNA5	A	Δ(779-859)	change in reading frame leading to a premature STOP codon at nt846
	B	Δ(490-868)	no change in reading frame but shorter protein (477 AA)
	C	Δ(792-858)	change in reading frame leading to a premature STOP codon at nt900
KCNC3	A		premature STOP codon at nt1518 leading to a shorter isoform (506 AA)
KCND1	A	Different Exon 3	premature stop codon and shorter protein
KCNQ1	A	Δ(1733-1794)	change in reading frame leading to a new C-terminus sequence
KCNQ2	A	Δ(1525-1527)	1 missing codon

Table S3. Transcriptome analysis of the three host cell lines, related to Figures S4 and 9

Absolute count values (in FPKM) of the expression of ion channel-related genes in CHO-FT, HEK-FT or CV1-FT cell lines, obtained by transcriptome sequencing. Alignment was done on CHO genome for CHO-FT cell line, and on human genome for HEK-FT and CV1-FT cell lines.
See attached xlsx file.

Table S4. Absolute count values of the multiplexed gene expression assay, related to Figure S3

See attached xlsx file.

Table S5. Cell counts, related to Figures 3, 5, 6, S7 and S9

Cell counts (N) for each ion channel group used for comparative chart of the Kv kinetic properties used in Figure 2, 3, 4, 5, 9, S7, S9, S13 and for overlay plots in Figure 6.
See attached xlsx file.

Table S6. Cloning primers list, related to Method "IC gene cloning"

List of primers used for PCR amplification and cloning in pENTR/TOPO vector of rat, mouse and human Kv genes.

Rat genes

Gene	Accession n.	Forward primer (5' to 3')	Reverse primer (5' to 3')
KCNA1	NM_173095	CACCATGACGGTGATGTCAGGGGAGA	TTAAACATCGGTCAGGAGC
KCNA2	NM_012970	CACCATGACAGTGGCTACCGGAGACCCAGTGGAT	TCAGACATCAGTTAACATTTTGGTAATATTCAC
KCNA3	NM_019270	CACCATGACCGTGGTGCCAGGGGACC	TTAGACATCAGTGAATATCTTTTTGA
KCNA4	NM_012971	CACCATGGAGGTGGCAATGGTGAG	TCACACATCAGTCTCCACAGCCT
KCNA5	NM_012972	CACCATGGAGATCTCCCTTGTGCC	TTACAAATCTGTTTCACGGCTAGT
KCNA6	NM_023954	CACCATGAGATCGGAGAAATCCCTGA	TCAAACCTCGGTGAGCATCCTT
KCNA7	NM_001108914	CACCATGCTATTTTTACGTGCGGACACC	TCACACCTCGGTCACCATGTGTT
KCNA10	NM_0001191713	CACCATGGATGTGTGTAGCTGGAGAG	TCACTTCCTGGACTTCTCTGCAG
KCNB1	NM_013186	CACCATGACGAAGCATGGCTCGCG	TCAGATACTCTGATCCCTAGTGC
KCNB2	NM_054000	CACCATGGCAGAAAAGGCACCTCC	TCACATGCTGGTCTCACGAGTGG
KCNC1	NM_012856	CACCATGGGCCAAGGGGACGAGA	TCAAGTCACTCTCACAGCCTCT
KCNC2	NM_139216	CACCATGGGCAAGATCGAGAACAACG	TTAAGTAAGAGATCTGGCCTCGG
KCNC3	NM_053997	CACCATGCTCAGCTCAGTGTGCGTCT	CTAGAGGATGGAGGGCAGGGTCG
KCNC4	NM_001122776	CACCATGATCAGCTCGGTTTGTGTC	TTAAAGAGCACAGGTGTGAGACA
KCND1	NM_001105748	CACCATGGCGGCAGGAGTGGC	TCACAGGGAAGAGATCTTGACAG
KCND2	NM_031730	CACCATGGCAGCCGGTGTTGC	TTACAAAGCAGACACCCTGACGA
KCND3	NM_001270963	CACCATGGCGGCAGGAGTTGC	TTAAATACACAGTGTTATAATTTCTTCC
KCNF1	NM_001169104	CACCATGGAACCGGGGCTTGC	TCACTTGCAGCTCTGTAGCC
KCNG1	NM_001106545	CACCATGACCCTGTTACCAGGAGACAA	TCAGTTGTTGTCCCTAGTGTCTG
KCNG2	NM_001107372	CACCATGGCCCGGCTCCCGGGGCAC	TCAGGGTCCCACCGGGCCTG
KCNG3	NM_133426	CACCATGACCTTCGGGCGCG	TTAATTCAGGAACTCAGCGGAG
KCNG4	NM_001107435	CACCATGCCCATGTCTTCCCGAG	CTACATGTAGCGGCCTGGGGAG
KCNQ1	NM_032073	CACCATGGACACGGCTTCCTCCCC	TCAGGAGCCTTCATCAGGGCCTG
KCNQ2	NM_133322	CACCATGGTGCAAAAGTCGCGCAAC	TCACTTCCTAGGCCCTGCCCAA
KCNQ3	NM_031597	CACCATGGGGCTCAAGGCTCGCA	CTAAGTGGGCTTGTTGGAAGGGG
KCNQ4	XM_008764109	CACCATGGCCGAGGCCCCCCCG	TCAGTCCATGTTGGTGCTGACTG
KCNQ5	NM_001134643	F1-GCCGCCCCCTTCACCATGCCCCGCCACCACGC	R1-AACTTCCGCTTTGCAACATGAAACTTCATGA TTCTGA
		F2-TGCAAAGCGGAAGTTTAAGGAAACATTACGC CCATAT	R2- GGCGCGCCCACCCTTTTAGTTCAGTTTGACGTGAG GCA
KCNV1	NM_021697	CACCATGGATCTGTCACCCCGCAACC	TTAAAACCAGAAATCATCTCCACC
KCNV2	NM_001106370	CACCATGCTGAAACAGAGCACCGAGA	TTAATTCTCTTGCCTTGTGGCGGACTG
KCNS1	NM_053954	CACCATGGTGAGCGAGTTTCCGGGTCC	TTAATAACTGTGAGATTTTGCAGGC
KCNS2	NM_023966	CACCATGACCCGCCAGAGCCTGTG	CTAATGTAGAGAGTCGTCTAAAC
KCNS3	NM_031778	CACCATGGTGTTTGGCGAGTTTTTCC	TCATTTTGCAGTACAGTTCTCCA
KCNH1	Not available	CACCATGACCATGGCTGGCGG	TCAGCTTGCCCCAAAAATGTCTC
KCNH5	NM_133610	F1-GCCGCCCCCTTCACCATGCCGGGGGGCAAGA GAG	R1-CTAAGGCCTTTCGGGACCTGATATAATTTCA GGA
		F2-CCCGAAAGGCCTTAGTGAACGAGTCATGGAT TAC	R2-GGCGCGCCCACCCTTTTAAAAGTTGATTTCA TCTTTG
KCNH2	NM_053949	CACCATGCCGGTGCGGAGGGGC	CTAACTGCCTGGATCTGAGCC
KCNH6	NM_053937	CACCATGCCAGTCCGCAGGGG	CTAACTCCTTGTGAATCCAGGATCGGA
KCNH7	NM_131912	CACCATGCCTGTTCGCAGGGG	TTACTTTCCCGGAAGACCAGGAT
KCNH8	NM_145095	CACCATGCCGGTTATGAAAGGATTGCT	TTATACGTTTATGGCTTTGCTGT

KCNH3	NM_017108	F1-GCCGCCCCCTTCACCATGCCGGCCATGCGGGGGCTC	R1-TCGAGCATGCGCTGCTTGAGGGGCTTGGGGATGC
		F2-GCAGCGCATGCTCGAGTACTTCCAAGCCACCTGG	R2-GGCGCGCCCACCCTTTCAGACTCCTGTGCCCTCCT
KCNH4	NM_053630	F1-GCCGCCCCCTTCACCATGCCGGTCATGAAAGGGTTGCTG	R1-GACGATGCACTCGGATGAAGTCCTTGAGATC
		F2-CATCCGAGTGCATCGTCTGCCCCGCCCACT	R2-GGCGCGCCCACCCTTTCAGTGGAATGTGTCTGACCCAG

Mouse genes

Gene	Accession n.	Forward primer (5' to 3')	Reverse primer (5' to 3')
KCNA1	NM_010595	CACCATGACGGTGATGTCAGGGGAGA	TTAAACATCGGTCAGGAGC
KCNA4	NM_021275	CACCATGGAGGTGGCAATGGTGAG	TCACACATCAGTCTCCACAGCCT
KCNA5	NM_145983	CACCATGGAGATCTCCCTTGTGCC	TTACAAATCTGTTTCACGGCTAGT
KCNA6	NM_013568	CACCATGAGATCGGAGAAATCCCTGA	TCAAACCTCGGTGAGCATCCTT
KCNB1	NM_008420	CACCATGCCGGCGGGTATGACGAA	TCAGATACTCTGATCCCTAGTGC

Human genes

Gene	Accession n.	Forward primer (5' to 3')	Reverse primer (5' to 3')
KCNA1	NM_000217	CACCATGACGGTGATGTCTGGGGAGAA	TTAAACATCGGTCAGTAGCTTGC
KCNA4	NM_002233	CACCATGGAGGTGGCAATGGTGAG	TCACACATCAGTCTCCACAGCCT
KCNA5	NM_002234	CACCATGGAGATCGCCCTAGTGC	TCACAAATCTGTTTCCCGGCTGG
KCNA6	NM_002235	CACCATGAGATCGGAGAAATCCCTTAC	TCAGACCTCCGTGAGCATTCTTT
KCNB1	NM_004975	CACCATGCCGGCGGGTATGACGAA	TCAGATGCTCTGATCTCGTGTGC

Table S7. Screening primers list, related to Method "Cell line validation by RT-PCR"

List of primers used for the amplification of the Kv genes by RT-PCR for cell lines validation, with expected amplification size.

Gene	Species	Forward primer (5' to 3')	Reverse primer (5' to 3')	Size (bp)
KCNA1	rat, mouse, human	CATGACCACTGTGGGATACG	AGCAACTGAGCCTGCTCTTC	177
KCNA2	rat	TTATCACCCTGGGGACAGAG	TGACCCCAATGAAGAGGAAG	219
KCNA3	rat	GGAGACCCTGTGCATCATCT	TCAGGATGGCCAGTGACATA	197
KCNA4	rat, mouse, human	CGGGTGTCTTAACCATTGCT	TGACTTGTCCCCCAGGGAAG	188
KCNA5	rat, mouse	CTCCGACGTCTGGACTCAAT	CCCGGAGATGTTTATGAGGA	248
	human	CGAGGATGAGGGCTTCATTA	CTGAACTCAGGCAGGGTCTC	186
KCNA6	rat, mouse	CCGGTACCGGTCATTGTCTC	TCTGCATAAGCTCGATGTGG	208
	human	GCGGCTGGTGATCAATATCT	CTCCTCCAGGAAAATGTCCA	238
KCNA7	rat	TTCTTTGTGGTGGAGACCCT	TAGGATGGCCAGGGACATAG	205
KCNA10	rat	ATCTTCAAGCTCTCCCGACA	GTTGTCATGGTGACCACAGC	212
KCNB1	rat	AAGGAGCAGATGAACGAGGA	GCGATGGTGGACAGGACGAT	194
	mouse	AAGGAGCAGATGAACGAGGA	GCAATGGTGGAGAGGACAAT	194
	human	AAAGAGCAGATGAACGAGGA	GCAATGGTGGAGAGGACGAT	194
KCNB2	rat	CCCTTCCTATCCCCATCATT	TTGATATTGGCCGACTCTCC	208
KCNC1	rat	GAAGACTGCCCCCACATAGA	CACTCTCACAGCCTCTGTCG	204
KCNC2	rat	GTACCCCCAAACATGGTCAG	GTAGGTGAGCTTGCCAGAGG	192
KCNC3	rat	TTATGGGATTGCCTCCTCTG	TGATCGGGCTCTTGTCTTCT	183
KCNC4	rat	CAGAAGCTTCCCAAGAAACG	TAGCGTCACCATTCTGCTTG	184
KCND1	rat	CCTACGTGTGTTTCGGGTCT	GGGATGCTTGTGAAGTTGGT	201
KCND2	rat	TGAGTGCATCTCGGCTTATG	TCTGCCTAGCAGTCATGGTG	182
KCND3	rat	GGCTACACCCTGAAGAGCTG	GCCAAATATCTTCCCAGCAA	222
KCNF1	rat	CCTGCAGACCCTCACCTACG	CACTGTGGTCATGGTGATGA	186
KCNG1	rat	CGAGAATGAGATGGCAGACA	ATCAGCACCCGTTCTTGTTC	243
KCNG2	rat	CATCCTGCCCTTCTACGTGT	GAGGAAGAGCAGCAGCAGTC	214
KCNG3	rat	CGATGCTACCGAGAGATGGT	ACGATCCCACTGACAACACA	245
KCNG4	rat	CTGGAGCACCCTGGATGCCT	ATGCCCCAGTAGCTCAGCTC	215
KCNQ1	rat	GGCGCATGCAGTACTTTGTA	TGTCACCTTGTCTTCCACCC	245
KCNQ2	rat	TCGACATCATGGTGCTGATT	CCAATGTACCACGCAGTCAC	205
KCNQ3	rat	ATCGGGTTCGCCTTTCTAAT	TCTTCTGTCATGGGGTCTCC	214
KCNQ4	rat	AGTATGCGCTTCCTGCAGAT	GAGTTGGCATCCTTCTCAGC	182
KCNQ5	rat	TTGTTGCCTCCAAGGAAAAC	GAGCCACTTGACTCACTGCC	208
KCNV1	rat	TCCGACAAAAGCTCTGGGAT	AGGAAGTGGCACCTGTCTTT	238
KCNV2	rat	ACTCCAACCCAGCTGTCTAC	CTTTGCCTCGTAGTCATCGC	162
KCNS1	rat	ACAAGTGTTTCGCCTCATGC	GTCGAAGCCCACGTTCTTTT	193
KCNS2	rat	CTGAGCTCTTCCCCTACGTG	TCTCCTGCTCAGGTTCCACT	177
KCNS3	rat	GAAGTGGATGACCCTGTGCT	ACGTGGCATAGAAGGGAATG	163
KCNH1	rat	TGGTCCCTTACAACGTCTCC	ACTGAACAGGCTGCTGATGC	271
KCNH5	rat	ATGCCAACACCAACCGTTAC	TCAGCTCTCATGTCCTTGGG	181
KCNH2	rat	TGGATGCACTCCAGAGACAG	AACCTGAGAAAGCGAGTCCA	209
KCNH6	rat	GAAGCTGGACCGCTATTCTG	GGCGGTGACATACTTGTCCT	235
KCNH7	rat	ATCCCGCAACTCACTCTGAA	CACTGCTTTGAAAGGGCTGT	234
KCNH8	rat	AAAACCAAGCAGGAAGCTGA	ACGCAGCTCCAATCTCTGAT	238
KCNH3	rat	GAGTACCTCATTCACCAAGG	GGGGCAAACTCAGGGTACAG	250
KCNH4	rat	AATGTCACAGCCATCATCCA	GTTGACGGCCCATGTAGTCT	153
GAPDH	rat	TTGTGATGGGTGTGAACCAC	GGATGCAGGGATGATGTTCT	238
GAPDH	human	CGAGATCCCTCCAAAATCAA	TGACGATCTTGAGGCTGTTG	206

Table S8. APC automation steps, related to Method "APC automation"

Steps	Sub-steps	Comment
Initialize	Initialize amplifier and Patchliner	Specify stimulus protocol file. Location of IC/EC/SE solutions
	Initialize log file with meta data	User, experiment details, cell line, passage, induction, temperature, cell count
Prepare chips	Fill IC and EC	Volume 10 µl, Speed 40
Catch cells	Set pressure	-100 mBar
	Set Vhold -20 mV	
	Get cells from cell hotel	
	Wait 30 s	
	Set Vhold -80 mV	
Make seal	Add seal enhancer	
	Wait for 30 s	
Whole cell patch	Apply atmospheric pressure	
	Wait for whole cell	Suction is applied to the cells until the whole cell configuration is reached
	Wash with EC	To remove seal enhancer
Whole cell setting	Auto C-slow and R-series	Apply auto compensation for C-slow and R-series
	Seal quality control	If seal resistance is less than 100 M, then the channel gets turned off
Current clamp	Record resting potential of cells	
Voltage clamp	Apply Activation protocol	
Protocol loop	Auto C-slow	Apply auto compensation for C-slow
	Activation protocol	
	Ramp	
	Deactivation	
	AP	
	Inactivation	
	Wash with EC	
End loop		End loop if repetition >2
	Recovery	
Save files	Save files in format YYMMDD_00X	Where YY is year, MM is month, DD is date and X is the experiment number for a given day
Clean up	Clean pipettes and move to next recordings	
