# Supplementary material for: A Kinetic Map of the Homomeric Voltage-Gated Potassium Channel (Kv) Family
Source: Front Cell Neurosci. 2019 Aug 20;13:358. doi: 10.3389/fncel.2019.00358 (PMC6710402; doi:10.3389/fncel.2019.00358)
Supplement: Supplementary file 7 [file Data_Sheet_4.pdf]

Supplementary references

Abbott, G.W. (2016). Regulation of human cardiac potassium channels by full-length KCNE3 and KCNE4. Sci. Rep. *6*, 38412.

Aguilar, M.B., Pérez-Reyes, L.I., López, Z., de la Cotera, E.P.H., Falcón, A., Ayala, C., Galván, M., Salvador, C., and Escobar, L.I. (2010). Peptide sr11a from Conus spurius is a novel peptide blocker for Kv1 potassium channels. Peptides *31*, 1287–1291.

Akhtar, S., McIntosh, P., Bryan-Sisneros, A., Barratt, L., Robertson, B., and Dolly, J.O. (1999). A functional spliced-variant of beta 2 subunit of Kv1 channels in C6 glioma cells and reactive astrocytes from rat lesioned cerebellum. Biochemistry *38*, 16984–16992.

Akhtar, S., Shamotienko, O., Papakosta, M., Ali, F., and Dolly, J.O. (2002). Characteristics of brain Kv1 channels tailored to mimic native counterparts by tandem linkage of alpha subunits: implications for K+ channelopathies. J. Biol. Chem. *277*, 16376–16382.

Al-Sabi, A., Kaza, S.K., Dolly, J.O., and Wang, J. (2013). Pharmacological characteristics of Kv1.1- and Kv1.2-containing channels are influenced by the stoichiometry and positioning of their α subunits. Biochem. J. *454*, 101–108.

Aréchiga-Figueroa, I.A., Delgado-Ramírez, M., Morán-Zendejas, R., and Rodríguez-Menchaca, A.A. (2015). Modulation of Kv2.1 channels inactivation by curcumin. Pharmacol. Rep. PR *67*, 1273–1279.

Bähring, R., Vardanyan, V., and Pongs, O. (2004). Differential modulation of Kv1 channel-mediated currents by co-expression of Kvbeta3 subunit in a mammalian cell-line. Mol. Membr. Biol. *21*, 19–25.

Baranauskas, G., Tkatch, T., Nagata, K., Yeh, J.Z., and Surmeier, D.J. (2003). Kv3.4 subunits enhance the repolarizing efficiency of Kv3.1 channels in fast-spiking neurons. Nat. Neurosci. *6*, 258–266.

Bardien-Kruger, S., Wulff, H., Arieff, Z., Brink, P., Chandy, K.G., and Corfield, V. (2002). Characterisation of the human voltage-gated potassium channel gene, KCNA7, a candidate gene for inherited cardiac disorders, and its exclusion as cause of progressive familial heart block I (PFHBI). Eur. J. Hum. Genet. EJHG *10*, 36–43.

Beck, E.J., Bowlby, M., An, W.F., Rhodes, K.J., and Covarrubias, M. (2002). Remodelling inactivation gating of Kv4 channels by KChIP1, a small-molecular-weight calcium-binding protein. J. Physiol. *538*, 691–706.

Bentzen, B.H., Schmitt, N., Calloe, K., Dalby Brown, W., Grunnet, M., and Olesen, S.-P. (2006). The acrylamide (S)-1 differentially affects Kv7 (KCNQ) potassium channels. Neuropharmacology *51*, 1068–1077.

Bertoli, A., Moran, O., and Conti, F. (1994). Activation and deactivation properties of rat brain K+ channels of the Shaker-related subfamily. Eur. Biophys. J. EBJ *23*, 379–384.

Blaine, J.T., and Ribera, A.B. (1998). Heteromultimeric potassium channels formed by members of the Kv2 subfamily. J. Neurosci. Off. J. Soc. Neurosci. *18*, 9585–9593.

Blom, S.M., Schmitt, N., and Jensen, H.S. (2009). The acrylamide (S)-2 as a positive and negative modulator of Kv7 channels expressed in Xenopus laevis oocytes. PloS One *4*, e8251.

Blom, S.M., Rottländer, M., Kehler, J., Bundgaard, C., Schmitt, N., and Jensen, H.S. (2014). From pan-reactive KV7 channel opener to subtype selective opener/inhibitor by addition of a methyl group. PloS One *9*, e100209.

Brown, M.R., El-Hassar, L., Zhang, Y., Alvaro, G., Large, C.H., and Kaczmarek, L.K. (2016). Physiological modulators of Kv3.1 channels adjust firing patterns of auditory brain stem neurons. J. Neurophysiol. *116*, 106–121.

Chen, S.-H., Fu, S.-J., Huang, J.-J., and Tang, C.-Y. (2016). The episodic ataxia type 1 mutation I262T alters voltage-dependent gating and disrupts protein biosynthesis of human Kv1.1 potassium channels. Sci. Rep. *6*, 19378.

Choveau, F.S., Hernandez, C.C., Bierbower, S.M., and Shapiro, M.S. (2012a). Pore determinants of KCNQ3 K+ current expression. Biophys. J. *102*, 2489–2498.

Choveau, F.S., Bierbower, S.M., and Shapiro, M.S. (2012b). Pore helix-S6 interactions are critical in governing current amplitudes of KCNQ3 K+ channels. Biophys. J. *102*, 2499–2509.

Chuang, C.-C., Jow, G.-M., Lin, H.-M., Weng, Y.-H., Hu, J.-H., Peng, Y.-J., Chiu, Y.-C., Chiu, M.-M., and Jeng, C.-J. (2014). The punctate localization of rat Eag1 K+ channels is conferred by the proximal post-CNBHD region. BMC Neurosci. *15*, 23.

Clancy, S.M., Chen, B., Bertaso, F., Mamet, J., and Jegla, T. (2009). KCNE1 and KCNE3 beta-subunits regulate membrane surface expression of Kv12.2 K(+) channels in vitro and form a tripartite complex in vivo. PloS One *4*, e6330.

D'Adamo, M.C., Gallenmüller, C., Servettini, I., Hartl, E., Tucker, S.J., Arning, L., Biskup, S., Grottesi, A., Guglielmi, L., Imbrici, P., et al. (2014). Novel phenotype associated with a mutation in the KCNA1(Kv1.1) gene. Front. Physiol. *5*, 525.

Diochot, S., Schweitz, H., Béress, L., and Lazdunski, M. (1998). Sea anemone peptides with a specific blocking activity against the fast inactivating potassium channel Kv3.4. J. Biol. Chem. *273*, 6744–6749.

Dong, W.-H., Chen, J.-C., He, Y.-L., Xu, J.-J., and Mei, Y.-A. (2013). Resveratrol inhibits K(v)2.2 currents through the estrogen receptor GPR30-mediated PKC pathway. Am. J. Physiol. Cell Physiol. *305*, C547-557.

Elinder, F., Madeja, M., and Arhem, P. (1996). Surface Charges of K channels. Effects of strontium on five cloned channels expressed in Xenopus oocytes. J. Gen. Physiol. *108*, 325–332.

Ferber, M., Al-Sabi, A., Stocker, M., Olivera, B.M., and Terlau, H. (2004). Identification of a mammalian target of kappaM-conotoxin RIIIK. Toxicon Off. J. Int. Soc. Toxinology *43*, 915–921.

Fernandez, F.R., Morales, E., Rashid, A.J., Dunn, R.J., and Turner, R.W. (2003). Inactivation of Kv3.3 potassium channels in heterologous expression systems. J. Biol. Chem. *278*, 40890–40898.

Finol-Urdaneta, R.K., Strüver, N., and Terlau, H. (2006). Molecular and Functional Differences between Heart mKv1.7 Channel Isoforms. J. Gen. Physiol. *128*, 133–145.

Gamper, N., Stockand, J.D., and Shapiro, M.S. (2003). Subunit-specific modulation of KCNQ potassium channels by Src tyrosine kinase. J. Neurosci. Off. J. Soc. Neurosci. *23*, 84–95.

García-Fernández, R., Peigneur, S., Pons, T., Alvarez, C., González, L., Chávez, M.A., and Tytgat, J. (2016). The Kunitz-Type Protein ShPI-1 Inhibits Serine Proteases and Voltage-Gated Potassium Channels. Toxins *8*, 110.

Gilling, M., Rasmussen, H.B., Calloe, K., Sequeira, A.F., Baretto, M., Oliveira, G., Almeida, J., Lauritsen, M.B., Ullmann, R., Boonen, S.E., et al. (2013). Dysfunction of the Heteromeric KV7.3/KV7.5 Potassium Channel is Associated with Autism Spectrum Disorders. Front. Genet. *4*, 54.

Gòmez-Hernandez, J.M., Lorra, C., Pardo, L.A., Stühmer, W., Pongs, O., Heinemann, S.H., and Elliott, A.A. (1997). Molecular basis for different pore properties of potassium channels from the rat brain Kv1 gene family. Pflugers Arch. *434*, 661–668.

Guihard, G., Bellocq, C., Grelet, E., and Escande, D. (2003). Human Kv1.6 current displays a C-type-like inactivation when re-expressed in cos-7 cells. Biochem. Biophys. Res. Commun. *311*, 83–89.

Hatton, W.J., Mason, H.S., Carl, A., Doherty, P., Latten, M.J., Kenyon, J.L., Sanders, K.M., and Horowitz, B. (2001). Functional and molecular expression of a voltage-dependent K(+) channel (Kv1.1) in interstitial cells of Cajal. J. Physiol. *533*, 315–327.

Heinemann, S.H., Rettig, J., Graack, H.R., and Pongs, O. (1996). Functional characterization of Kv channel beta-subunits from rat brain. J. Physiol. *493*, 625–633.

Hernández-Pineda, R., Chow, A., Amarillo, Y., Moreno, H., Saganich, M., Vega-Saenz de Miera, E.C., Hernández-Cruz, A., and Rudy, B. (1999). Kv3.1-Kv3.2 channels underlie a high-voltage-activating component of the delayed rectifier K+ current in projecting neurons from the globus pallidus. J. Neurophysiol. *82*, 1512–1528.

Honoré, E., Attali, B., Romey, G., Lesage, F., Barhanin, J., and Lazdunski, M. (1992). Different types of K+ channel current are generated by different levels of a single mRNA. EMBO J. *11*, 2465–2471.

Hou, P., Zhang, R., Liu, Y., Feng, J., Wang, W., Wu, Y., and Ding, J. (2014). Physiological role of Kv1.3 channel in T lymphocyte cell investigated quantitatively by kinetic modeling. PloS One *9*, e89975.

Hsu, P.-H., Miaw, S.-C., Chuang, C.-C., Chang, P.-Y., Fu, S.-J., Jow, G.-M., Chiu, M.-M., and Jeng, C.-J. (2012). 14-3-3θ is a binding partner of rat Eag1 potassium channels. PloS One *7*, e41203.

Jentsch, T.J. (2000). Neuronal KCNQ potassium channels: physiology and role in disease. Nat. Rev. Neurosci. *1*, 21–30.

Jeong, I., Choi, B.H., and Hahn, S.J. (2011). Rosiglitazone inhibits Kv4.3 potassium channels by open-channel block and acceleration of closed-state inactivation. Br. J. Pharmacol. *163*, 510–520.

Jeong, I., Choi, B.H., Yoon, S.H., and Hahn, S.J. (2012). Carvedilol blocks the cloned cardiac Kv1.5 channels in a β-adrenergic receptor-independent manner. Biochem. Pharmacol. *83*, 497–505.

Jerng, H.H., Shahidullah, M., and Covarrubias, M. (1999). Inactivation gating of Kv4 potassium channels: molecular interactions involving the inner vestibule of the pore. J. Gen. Physiol. *113*, 641–660.

Jerng, H.H., Qian, Y., and Pfaffinger, P.J. (2004). Modulation of Kv4.2 channel expression and gating by dipeptidyl peptidase 10 (DPP10). Biophys. J. *87*, 2380–2396.

Ju, M., and Wray, D. (2002). Molecular identification and characterisation of the human eag2 potassium channel. FEBS Lett. *524*, 204–210.

Kalman, K., Nguyen, A., Tseng-Crank, J., Dukes, I.D., Chandy, G., Hustad, C.M., Copeland, N.G., Jenkins, N.A., Mohrenweiser, H., Brandriff, B., et al. (1998). Genomic organization, chromosomal localization, tissue distribution, and biophysical characterization of a novel mammalian Shaker-related voltage-gated potassium channel, Kv1.7. J. Biol. Chem. *273*, 5851–5857.

Kanda, V.A., Lewis, A., Xu, X., and Abbott, G.W. (2011). KCNE1 and KCNE2 provide a checkpoint governing voltage-gated potassium channel α-subunit composition. Biophys. J. *101*, 1364–1375.

Kanemasa, T., Gan, L., Perney, T.M., Wang, L.Y., and Kaczmarek, L.K. (1995). Electrophysiological and pharmacological characterization of a mammalian Shaw channel expressed in NIH 3T3 fibroblasts. J. Neurophysiol. *74*, 207–217.

Kazmierczak, M., Zhang, X., Chen, B., Mulkey, D.K., Shi, Y., Wagner, P.G., Pivaroff-Ward, K., Sassic, J.K., Bayliss, D.A., and Jegla, T. (2013). External pH modulates EAG superfamily K+ channels through EAG-specific acidic residues in the voltage sensor. J. Gen. Physiol. *141*, 721–735.

Kerschensteiner, D., Monje, F., and Stocker, M. (2003). Structural determinants of the regulation of the voltage-gated potassium channel Kv2.1 by the modulatory α-subunit Kv9.3. J. Biol. Chem. *278*, 18154–18161.

Kerschensteiner, D., Soto, F., and Stocker, M. (2005). Fluorescence measurements reveal stoichiometry of K+ channels formed by modulatory and delayed rectifier alpha-subunits. Proc. Natl. Acad. Sci. U. S. A. *102*, 6160–6165.

Kihira, Y., Hermanstyne, T.O., and Misonou, H. (2010). Formation of heteromeric Kv2 channels in mammalian brain neurons. J. Biol. Chem. *285*, 15048–15055.

Kim, K.S., Duignan, K.M., Hawryluk, J.M., Soh, H., and Tzingounis, A.V. (2016). The Voltage Activation of Cortical KCNQ Channels Depends on Global PIP2 Levels. Biophys. J. *110*, 1089–1098.

Kinoshita, M., Matsuoka, Y., Suzuki, T., Mirrielees, J., and Yang, J. (2012). Sigma-1 receptor alters the kinetics of Kv1.3 voltage gated potassium channels but not the sensitivity to receptor ligands. Brain Res. *1452*, 1–9.

Kuryshev, Y.A., Wible, B.A., Gudz, T.I., Ramirez, A.N., and Brown, A.M. (2001). KChAP/Kvbeta1.2 interactions and their effects on cardiac Kv channel expression. Am. J. Physiol. Cell Physiol. *281*, C290-299.

Lang, R., Lee, G., Liu, W., Tian, S., Rafi, H., Orias, M., Segal, A.S., and Desir, G.V. (2000). KCNA10: a novel ion channel functionally related to both voltage-gated potassium and CNG cation channels. Am. J. Physiol. Renal Physiol. *278*, F1013-1021.

Lee, H.M., Hahn, S.J., and Choi, B.H. (2016). Blockade of Kv1.5 channels by the antidepressant drug sertraline. Korean J. Physiol. Pharmacol. Off. J. Korean Physiol. Soc. Korean Soc. Pharmacol. *20*, 193–200.

Lee, J.-H., Choi, S.-H., Shin, T.-J., Lee, B.-H., Hwang, S.-H., Kim, H.-C., and Nah, S.-Y. (2011). Effect of dextromethorphan on human K(v)1.3 channel activity: involvement of C-type inactivation. Eur. J. Pharmacol. *651*, 122–127.

Lewis, A., McCrossan, Z.A., and Abbott, G.W. (2004). MinK, MiRP1, and MiRP2 diversify Kv3.1 and Kv3.2 potassium channel gating. J. Biol. Chem. *279*, 7884–7892.

Li, Y., Gamper, N., and Shapiro, M.S. (2004). Single-channel analysis of KCNQ K+ channels reveals the mechanism of augmentation by a cysteine-modifying reagent. J. Neurosci. Off. J. Soc. Neurosci. *24*, 5079–5090.

MacDonald, P.E., Salapatek, A.M.F., and Wheeler, M.B. (2003). Temperature and redox state dependence of native Kv2.1 currents in rat pancreatic beta-cells. J. Physiol. *546*, 647–653.

Macica, C.M., von Hehn, C.A.A., Wang, L.-Y., Ho, C.-S., Yokoyama, S., Joho, R.H., and Kaczmarek, L.K. (2003). Modulation of the kv3.1b potassium channel isoform adjusts the fidelity of the firing pattern of auditory neurons. J. Neurosci. Off. J. Soc. Neurosci. *23*, 1133–1141.

Malin, S.A., and Nerbonne, J.M. (2002). Delayed rectifier K+ currents, IK, are encoded by Kv2 alpha-subunits and regulate tonic firing in mammalian sympathetic neurons. J. Neurosci. Off. J. Soc. Neurosci. *22*, 10094–10105.

Mauerhöfer, M., and Bauer, C.K. (2016). Effects of Temperature on Heteromeric Kv11.1a/1b and Kv11.3 Channels. Biophys. J. *111*, 504–523.

Mestre, T.A., Manole, A., MacDonald, H., Riazi, S., Kraeva, N., Hanna, M.G., Lang, A.E., Männikkö, R., and Yoon, G. (2016). A novel KCNA1 mutation in a family with episodic ataxia and malignant hyperthermia. Neurogenetics *17*, 245–249.

Meyer, R., and Heinemann, S.H. (1997). Temperature and pressure dependence of Shaker K+ channel N- and C-type inactivation. Eur. Biophys. J. EBJ *26*, 433–445.

Miceli, F., Cilio, M.R., Taglialatela, M., and Bezanilla, F. (2009). Gating currents from neuronal K(V)7.4 channels: general features and correlation with the ionic conductance. Channels Austin Tex *3*, 274–283.

Miyake, A., Mochizuki, S., Yokoi, H., Kohda, M., and Furuichi, K. (1999). New ether-à-go-go K(+) channel family members localized in human telencephalon. J. Biol. Chem. *274*, 25018–25025.

Mock, A.F., Richardson, J.L., Hsieh, J.-Y., Rinetti, G., and Papazian, D.M. (2010). Functional effects of spinocerebellar ataxia type 13 mutations are conserved in zebrafish Kv3.3 channels. BMC Neurosci. *11*, 99.

Moran, O., and Conti, F. (1995). Properties of the Kv1.1 rat brain potassium channels expressed in mammalian cells: temperature effects. Biochem. Biophys. Res. Commun. *215*, 915–920.

Moreno, H., Kentros, C., Bueno, E., Weiser, M., Hernandez, A., Vega-Saenz de Miera, E., Ponce, A., Thornhill, W., and Rudy, B. (1995). Thalamocortical projections have a K+ channel that is phosphorylated and modulated by cAMP-dependent protein kinase. J. Neurosci. Off. J. Soc. Neurosci. *15*, 5486–5501.

Mortensen, L.S., Schmidt, H., Farsi, Z., Barrantes-Freer, A., Rubio, M.E., Ufartes, R., Eilers, J., Sakaba, T., Stühmer, W., and Pardo, L.A. (2015). KV 10.1 opposes activity-dependent increase in Ca^{2+} influx into the presynaptic terminal of the parallel fibre-Purkinje cell synapse. J. Physiol. *593*, 181–196.

Mourre, C., Chernova, M.N., Martin-Eauclaire, M.F., Bessone, R., Jacquet, G., Gola, M., Alper, S.L., and Crest, M. (1999). Distribution in rat brain of binding sites of kaliotoxin, a blocker of Kv1.1 and Kv1.3 alpha-subunits. J. Pharmacol. Exp. Ther. *291*, 943–952.

Nakajo, K., and Kubo, Y. (2008). Second coiled-coil domain of KCNQ channel controls current expression and subfamily specific heteromultimerization by salt bridge networks. J. Physiol. *586*, 2827–2840.

Nicolaou, S.A., Szigligeti, P., Neumeier, L., Lee, S.M., Duncan, H.J., Kant, S.K., Mongey, A.B., Filipovich, A.H., and Conforti, L. (2007). Altered dynamics of Kv1.3 channel compartmentalization in the immunological synapse in systemic lupus erythematosus. J. Immunol. Baltim. Md 1950 *179*, 346–356.

Noma, K., Kimura, K., Minatohara, K., Nakashima, H., Nagao, Y., Mizoguchi, A., and Fujiyoshi, Y. (2009). Triple N-glycosylation in the long S5-P loop regulates the activation and trafficking of the Kv12.2 potassium channel. J. Biol. Chem. *284*, 33139–33150.

Oliver, K.L., Franceschetti, S., Milligan, C.J., Muona, M., Mandelstam, S.A., Canafoglia, L., Boguszewska-Chachulska, A.M., Korczyn, A.D., Bisulli, F., Di Bonaventura, C., et al. (2017). Myoclonus epilepsy and ataxia due to KCNC1 mutation: Analysis of 20 cases and K+ channel properties. Ann. Neurol. *81*, 677–689.

Oliveras, A., Roura-Ferrer, M., Solé, L., de la Cruz, A., Prieto, A., Etxebarria, A., Manils, J., Morales-Cano, D., Condom, E., Soler, C., et al. (2014). Functional assembly of Kv7.1/Kv7.5 channels with emerging properties on vascular muscle physiology. Arterioscler. Thromb. Vasc. Biol. *34*, 1522–1530.

Orhan, G., Bock, M., Schepers, D., Ilina, E.I., Reichel, S.N., Löffler, H., Jezutkovic, N., Weckhuysen, S., Mandelstam, S., Suls, A., et al. (2014). Dominant-negative effects of KCNQ2 mutations are associated with epileptic encephalopathy. Ann. Neurol. *75*, 382–394.

Pahapill, P.A., and Schlichter, L.C. (1990). Modulation of potassium channels in human T lymphocytes: effects of temperature. J. Physiol. *422*, 103–126.

Peretz, A., Pell, L., Gofman, Y., Haitin, Y., Shamgar, L., Patrich, E., Kornilov, P., Gourgy-Hacohen, O., Ben-Tal, N., and Attali, B. (2010). Targeting the voltage sensor of Kv7.2 voltage-gated K+ channels with a new gating-modifier. Proc. Natl. Acad. Sci. U. S. A. *107*, 15637–15642.

Pérez-García, M.T., López-López, J.R., and González, C. (1999). Kvbeta1.2 subunit coexpression in HEK293 cells confers O2 sensitivity to kv4.2 but not to Shaker channels. J. Gen. Physiol. *113*, 897–907.

Po, S., Roberds, S., Snyders, D.J., Tamkun, M.M., and Bennett, P.B. (1993). Heteromultimeric assembly of human potassium channels. Molecular basis of a transient outward current? Circ. Res. *72*, 1326–1336.

Rangaraju, S., Khoo, K.K., Feng, Z.-P., Crossley, G., Nugent, D., Khaytin, I., Chi, V., Pham, C., Calabresi, P., Pennington, M.W., et al. (2010). Potassium channel modulation by a toxin domain in matrix metalloprotease 23. J. Biol. Chem. *285*, 9124–9136.

Rashid, A.J., Morales, E., Turner, R.W., and Dunn, R.J. (2001). The contribution of dendritic Kv3 K+ channels to burst threshold in a sensory neuron. J. Neurosci. Off. J. Soc. Neurosci. *21*, 125–135.

Remillard, C.V., Tigno, D.D., Platoshyn, O., Burg, E.D., Brevnova, E.E., Conger, D., Nicholson, A., Rana, B.K., Channick, R.N., Rubin, L.J., et al. (2007). Function of Kv1.5 channels and genetic variations of KCNA5 in patients with idiopathic pulmonary arterial hypertension. Am. J. Physiol. Cell Physiol. *292*, C1837-1853.

Rezazadeh, S., Kurata, H.T., Claydon, T.W., Kehl, S.J., and Fedida, D. (2007). An activation gating switch in Kv1.2 is localized to a threonine residue in the S2-S3 linker. Biophys. J. *93*, 4173–4186.

Rich, T.C., and Snyders, D.J. (1998). Evidence for multiple open and inactivated states of the hKv1.5 delayed rectifier. Biophys. J. *75*, 183–195.

Roeper, J., Sewing, S., Zhang, Y., Sommer, T., Wanner, S.G., and Pongs, O. (1998). NIP domain prevents N-type inactivation in voltage-gated potassium channels. Nature *391*, 390–393.

Rudy, B., Chow, A., Lau, D., Amarillo, Y., Ozaita, A., Saganich, M., Moreno, H., Nadal, M.S., Hernandez-Pineda, R., Hernandez-Cruz, A., et al. (1999). Contributions of Kv3 channels to neuronal excitability. Ann. N. Y. Acad. Sci. *868*, 304–343.

Russell, S.N., Publicover, N.G., Hart, P.J., Carl, A., Hume, J.R., Sanders, K.M., and Horowitz, B. (1994). Block by 4-aminopyridine of a Kv1.2 delayed rectifier K+ current expressed in Xenopus oocytes. J. Physiol. *481 (Pt 3)*, 571–584.

Sankaranarayanan, K., Varshney, A., and Mathew, M.K. (2005). N type rapid inactivation in human Kv1.4 channels: functional role of a putative C-terminal helix. Mol. Membr. Biol. *22*, 389–400.

Schönherr, R., Gessner, G., Löber, K., and Heinemann, S.H. (2002). Functional distinction of human EAG1 and EAG2 potassium channels. FEBS Lett. *514*, 204–208.

Schrøder, R.L., Jespersen, T., Christophersen, P., Strøbaek, D., Jensen, B.S., and Olesen, S.P. (2001). KCNQ4 channel activation by BMS-204352 and retigabine. Neuropharmacology *40*, 888–898.

Smith, J.S., Iannotti, C.A., Dargis, P., Christian, E.P., and Aiyar, J. (2001). Differential expression of kcnq2 splice variants: implications to m current function during neuronal development. J. Neurosci. Off. J. Soc. Neurosci. *21*, 1096–1103.

Sprunger, L.K., Stewig, N.J., and O'Grady, S.M. (1996). Effects of charybdotoxin on K+ channel (KV1.2) deactivation and inactivation kinetics. Eur. J. Pharmacol. *314*, 357–364.

Stühmer, W., Ruppersberg, J.P., Schröter, K.H., Sakmann, B., Stocker, M., Giese, K.P., Perschke, A., Baumann, A., and Pongs, O. (1989). Molecular basis of functional diversity of voltage-gated potassium channels in mammalian brain. EMBO J. *8*, 3235–3244.

Sung, M.J., Hahn, S.J., and Choi, B.H. (2009). Effect of psoralen on the cloned Kv3.1 currents. Arch. Pharm. Res. *32*, 407–412.

Syrbe, S., Hedrich, U.B.S., Riesch, E., Djémié, T., Müller, S., Møller, R.S., Maher, B., Hernandez-Hernandez, L., Synofzik, M., Caglayan, H.S., et al. (2015). De novo loss- or gain-of-function mutations in KCNA2 cause epileptic encephalopathy. Nat. Genet. *47*, 393–399.

Tian, S., Liu, W., Wu, Y., Rafi, H., Segal, A.S., and Desir, G.V. (2002). Regulation of the voltage-gated K+ channel KCNA10 by KCNA4B, a novel beta-subunit. Am. J. Physiol. Renal Physiol. *283*, F142-149.

Tinel, N., Diochot, S., Borsotto, M., Lazdunski, M., and Barhanin, J. (2000). KCNE2 confers background current characteristics to the cardiac KCNQ1 potassium channel. EMBO J. *19*, 6326–6330.

Tipparaju, S.M., Li, X.-P., Kilfoil, P.J., Xue, B., Uversky, V.N., Bhatnagar, A., and Barski, O.A. (2012). Interactions between the C-terminus of Kv1.5 and Kvβ regulate pyridine nucleotide-dependent changes in channel gating. Pflugers Arch. *463*, 799–818.

Trudeau, M.C., Titus, S.A., Branchaw, J.L., Ganetzky, B., and Robertson, G.A. (1999). Functional analysis of a mouse brain Elk-type K+ channel. J. Neurosci. Off. J. Soc. Neurosci. *19*, 2906–2918.

Unsöld, B., Kerst, G., Brousos, H., Hübner, M., Schreiber, R., Nitschke, R., Greger, R., and Bleich, M. (2000). KCNE1 reverses the response of the human K+ channel KCNQ1 to cytosolic pH changes and alters its pharmacology and sensitivity to temperature. Pflugers Arch. *441*, 368–378.

Wang, H.S., Pan, Z., Shi, W., Brown, B.S., Wymore, R.S., Cohen, I.S., Dixon, J.E., and McKinnon, D. (1998). KCNQ2 and KCNQ3 potassium channel subunits: molecular correlates of the M-channel. Science *282*, 1890–1893.

Wang, S., Ding, W.-G., Bai, J.-Y., Toyoda, F., Wei, M.-J., and Matsuura, H. (2016). Regulation of human cardiac Kv1.5 channels by extracellular acidification. Pflugers Arch. *468*, 1885–1894.

Weiser, M., Vega-Saenz de Miera, E., Kentros, C., Moreno, H., Franzen, L., Hillman, D., Baker, H., and Rudy, B. (1994). Differential expression of Shaw-related K+ channels in the rat central nervous system. J. Neurosci. Off. J. Soc. Neurosci. *14*, 949–972.

Wettwer, E., and Terlau, H. (2014). Pharmacology of voltage-gated potassium channel Kv1.5--impact on cardiac excitability. Curr. Opin. Pharmacol. *15*, 115–121.

Wible, B., Murawsky, M.K., Crumb, W.J., and Rampe, D. (1997). Stable expression and characterization of the human brain potassium channel Kv2.1: blockade by antipsychotic agents. Brain Res. *761*, 42–50.

Wible, B.A., Yang, Q., Kuryshev, Y.A., Accili, E.A., and Brown, A.M. (1998). Cloning and expression of a novel K+ channel regulatory protein, KChAP. J. Biol. Chem. *273*, 11745–11751.

Xie, C., Su, H., Guo, T., Yan, Y., Peng, X., Cao, R., Wang, Y., Chen, P., Wang, X., and Liang, S. (2014). Synaptotagmin I delays the fast inactivation of Kv1.4 channel through interaction with its N-terminus. Mol. Brain *7*, 4.

Xie, G., Harrison, J., Clapcote, S.J., Huang, Y., Zhang, J.-Y., Wang, L.-Y., and Roder, J.C. (2010). A new Kv1.2 channelopathy underlying cerebellar ataxia. J. Biol. Chem. *285*, 32160–32173.

Yan, L., Liu, X., Liu, W.-X., Tan, X.-Q., Xiong, F., Gu, N., Hao, W., Gao, X., and Cao, J.-M. (2015). Fe2O3 nanoparticles suppress Kv1.3 channels via affecting the redox activity of Kvβ2 subunit in Jurkat T cells. Nanotechnology *26*, 505103.

Yang, F., and Zheng, J. (2014). High temperature sensitivity is intrinsic to voltage-gated potassium channels. ELife *3*.

Yang, T., Snyders, D.J., and Roden, D.M. (1997). Inhibition of cardiac potassium currents by the vesnarinone analog OPC-18790: comparison with quinidine and dofetilide. J. Pharmacol. Exp. Ther. *280*, 1170–1175.

Yang, Y., Xia, M., Jin, Q., Bendahhou, S., Shi, J., Chen, Y., Liang, B., Lin, J., Liu, Y., Liu, B., et al. (2004). Identification of a KCNE2 gain-of-function mutation in patients with familial atrial fibrillation. Am. J. Hum. Genet. *75*, 899–905.

Yang, Y., Vasylyev, D.V., Dib-Hajj, F., Veeramah, K.R., Hammer, M.F., Dib-Hajj, S.D., and Waxman, S.G. (2013). Multistate structural modeling and voltage-clamp analysis of epilepsy/autism mutation Kv10.2-R327H demonstrate the role of this residue in stabilizing the channel closed state. J. Neurosci. Off. J. Soc. Neurosci. *33*, 16586–16593.

Yeung, S.Y.M., Thompson, D., Wang, Z., Fedida, D., and Robertson, B. (2005). Modulation of Kv3 subfamily potassium currents by the sea anemone toxin BDS: significance for CNS and biophysical studies. J. Neurosci. Off. J. Soc. Neurosci. *25*, 8735–8745.

Yunoki, T., Takimoto, K., Kita, K., Funahashi, Y., Takahashi, R., Matsuyoshi, H., Naito, S., and Yoshimura, N. (2014). Differential contribution of Kv4-containing channels to A-type, voltage-gated potassium currents in somatic and visceral dorsal root ganglion neurons. J. Neurophysiol. *112*, 2492–2504.

Zaika, O., Hernandez, C.C., Bal, M., Tolstykh, G.P., and Shapiro, M.S. (2008). Determinants within the turret and pore-loop domains of KCNQ3 K+ channels governing functional activity. Biophys. J. *95*, 5121–5137.

Zhang, X., Bursulaya, B., Lee, C.C., Chen, B., Pivaroff, K., and Jegla, T. (2009). Divalent cations slow activation of EAG family K+ channels through direct binding to S4. Biophys. J. *97*, 110–120.

Zhang, Y., Zhang, X.-F., Fleming, M.R., Amiri, A., El-Hassar, L., Surguchev, A.A., Hyland, C., Jenkins, D.P., Desai, R., Brown, M.R., et al. (2016). Kv3.3 Channels Bind Hax-1 and Arp2/3 to Assemble a Stable Local Actin Network that Regulates Channel Gating. Cell *165*, 434–448.

Zhong, X.Z., Abd-Elrahman, K.S., Liao, C.-H., El-Yazbi, A.F., Walsh, E.J., Walsh, M.P., and Cole, W.C. (2010). Stromatoxin-sensitive, heteromultimeric Kv2.1/Kv9.3 channels contribute to myogenic control of cerebral arterial diameter. J. Physiol. *588*, 4519–4537.

Zhou, W., Qian, Y., Kunjilwar, K., Pfaffinger, P.J., and Choe, S. (2004). Structural insights into the functional interaction of KChIP1 with Shal-type K(+) channels. Neuron *41*, 573–586.

Zhu, S., Peigneur, S., Gao, B., Luo, L., Jin, D., Zhao, Y., and Tytgat, J. (2011). Molecular diversity and functional evolution of scorpion potassium channel toxins. Mol. Cell. Proteomics MCP *10*, M110.002832.

Zou, A., Lin, Z., Humble, M., Creech, C.D., Wagoner, P.K., Krafte, D., Jegla, T.J., and Wickenden, A.D. (2003). Distribution and functional properties of human KCNH8 (Elk1) potassium channels. Am. J. Physiol. Cell Physiol. *285*, C1356-1366.
